# Free water elimination tractometry for aging brains

**DOI:** 10.1162/IMAG.a.991

**Published:** 2025-11-07

**Authors:** Kelly Chang, Luke Burke, Nina LaPiana, Bradley Howlett, David Hunt, Margaret Dezelar, Jalal B. Andre, Patti Curl, James D. Ralston, Ariel Rokem, Christine L. Mac Donald

**Affiliations:** Department of Psychology, University of Washington, Seattle, WA, United States; Kaiser Permanente Washington Health Research Institute, Seattle, WA, United States; Department of Neurological Surgery, University of Washington, Seattle, WA, United States; Department of Radiology, University of Washington, Seattle, WA, United States

**Keywords:** free water elimination, diffusion imaging, tractography, tractometry, aging

## Abstract

Tractometry of diffusion-weighted magnetic resonance imaging (dMRI) non-invasively quantifies tissue properties of brain connections. It is widely used in aging studies but could be less reliable in aging brains due to increased white matter free water. We demonstrate that computational free water elimination (FWE) and multi-shell multi-tissue (MSMT) modeling both increase the reliability and accuracy of tractometry in a large (n = 396) cohort of older adults (65–103 y.o.). We found substantial improvements in reliability in a split-half comparison at every stage of the pipeline: estimation of voxel-level fiber orientation distribution functions, delineation of major pathway trajectories, and assessment of tissue properties along the pathways. FWE in particular provided better tractography yield, that included more coverage of areas of leukoaraiosis. However, tractometry was strongly predictive of Fazekas scores, which assess the extent of white matter hyperintensity (WMH) burden, regardless of method. This indicates that increased WMH burden is associated with global changes to white matter. By sub-sampling a multi-shell dataset, we demonstrated that these findings generalize to single-shell data, which is important for many datasets where only one *b*-value may be available. Overall, the results highlight the importance of accounting for free water in tractometry studies, especially in aging brains. We provide open-source software for free-water elimination that can be applied to a wide range of clinical and research datasets (https://github.com/nrdg/fwe).

## 1 Introduction

Tractometry based on diffusion MRI (dMRI) measurements provides accurate and reliable assessments of white matter tissue properties along the length of major white matter pathways, which can be used to make inferences about brain connections (Kruper et al., 2021; Takemura et al., 2024). Tractometry processing pipelines include several steps: modeling of individual-voxel fiber orientation distribution functions (fODF), computational tractography (Jeurissen et al., 2019), delineation of major white matter pathways (Yeatman et al., 2012), modeling of individual voxel tissue properties, and the projection of these tissue properties onto the length of each of the tracts. These so-called “tract profiles” are then used as the input to statistical analysis and inference.

Tractometry is particularly useful in assessing brain aging as brain connectivity is profoundly affected by aging, and the cognitive effects of brain disconnection in aging may be mediated by changes to brain white matter microstructure (Cox et al., 2016). Areas of leukoaraiosis, apparent as white matter hyperintensities (WMH) in fluid attenuated inversion recovery (FLAIR) imaging, may be particularly interesting to investigate with tractometry in aging subjects, because they are thought to be indicative of ischemic brain injury and related to its impact on cognition (Wardlaw et al., 2015). However, these are also regions in which the directional specificity of the diffusion signal along the trajectory of white matter fiber bundles is potentially confounded by the effects of excess cerebrospinal fluid (CSF) that may have infiltrated the white matter (Chang et al., 2023).

Computational free water elimination (FWE) provides a potential solution because it removes the confounding effect of free water on the diffusion signal (Hoy et al., 2014; Pasternak et al., 2009; Pierpaoli & Jones, 2004). Previous work has shown the benefits of this approach in improving tractography estimates of major white matter pathways (Hoy et al., 2015) and in tractography around regions of brain white matter edema (e.g., around tumors; Ould Ismail et al., 2019).

An alternative approach to addressing free water contamination is multi-shell multi-tissue (MSMT) constrained spherical deconvolution. MSMT accounts for free water contamination by modeling separate response functions for three tissue compartments, white matter (WM), gray matter (GM), and CSF (Jeurissen et al., 2019). By doing so, MSMT isolates the WM signal for fODF modeling while explicitly controlling for partial volume effects from GM and CSF. This separation allows MSMT to account for intruding free water, such as in WMH areas, where partial volume contamination is severe.

Here, we studied the impact of FWE on tractometry in multi-shell high angular resolution dMRI measurements in a large (n = 396) group of aging (ages 65–103 y.o.) participants from the Adult Changes in Thought (ACT) Study, a well-characterized, community-based, longitudinal, prospective cohort study (Kukull et al., 2002). Two methods to mitigate free water contamination were assessed: (1) free water elimination based on the free water diffusion tensor imaging (FWDTI) model (Golub et al., 2021; Henriques et al., 2017; Hoy et al., 2014; Pasternak et al., 2009), and (2) modeling using MSMT-constrained spherical deconvolution (Jeurissen et al., 2019). A split-half approach showed improvements in reliability at every step of the tractometry pipeline with both methods. The improvements from FWE and MSMT tractometry translated to single-shell dMRI measurements, which are more common in clinical research settings, even while improvements in reliability are more modest. However, we also observed that MSMT is susceptible to fODF misestimation errors in aging brains, particularly in regions with extensive WMH. Taken together, these results demonstrate that free water correction, through either FWE or MSMT, can provide benefit in a wide range of studies that use dMRI to understand brain connections in aging individuals.

## 2 Methods

### 2.1 Participants

A total of 396 individuals underwent a research-grade MRI scan which was leveraged for this analysis. Participants were a part of the Adult Changes in Thought (ACT) study, a longitudinal cohort study embedded in an integrated healthcare delivery system. The overall goal of the ACT study is to understand factors that contribute to Alzheimer’s Disease and related dementia. The ACT study prospectively and retrospectively collects data on participants through biannual assessments, electronic medical research abstraction, and through ancillary activities such as research-grade MRI scans, optical coherence tomography scans, and collection of biofluids for genetic and biomarker analysis (Kukull et al., 2002). The institutional review board at the Kaiser Permanente Research Institute approved the study protocol.

Participants were excluded from the analysis if their average neighboring dMRI correlation (NDC; Yeh et al., 2019)—a data quality metric, which we have previously shown to be closely related to expert observers (Richie-Halford et al., 2022) —fell 2 standard deviations below the sample average. This resulted in 369 participants (ages 65 - 101, mean age = 78.93; 194 females) remaining in the sample.

### 2.2 MRI acquisitions

Data were acquired at the University of Washington at the Diagnostic Imaging Sciences Center (DISC) on a 3T Philips Ingenia Elition MRI scanner with a 32-channel head coil. The current analysis leveraged the T1-weighted (T1w) and fluid attenuated inversion recovery (FLAIR) whole-brain structural images and two diffusion-weighted acquisitions of opposite phase encoding directions from each participant.

#### 2.2.1 T1w

3D MPRAGE T1w images were acquired at 1 mm^3^ isotropic resolution (TR = 6.5 ms, TE = 2.9 ms, flip angle = 9^o^, FOV = 256  × 256 mm, matrix size = 256  × 256 , 211 sagittal slices).

#### 2.2.2 FLAIR

3D FLAIR images were acquired with an inversion recovery sequence at 1 mm^3^ isotropic (TR = 5000, TE = 291 ms, TI = 1800 ms, flip angle = 90^o^, FOV = 256 ​​× 256 mm, matrix size = 256  × 256 , 176 sagittal slices).

#### 2.2.3 Diffusion-weighted imaging

Diffusion-weighted images were acquired with a spin-echo echo-planar imaging sequence at an in-plane spatial resolution of 2.2  × 2.2 mm^2^ (TR = 3500 ms, TE = 89 ms, FOV = 246.4  ×  246.4 mm, matrix size = 112  × 112 , slice thickness = 2.2 mm, 57 axial slices). The dMRI data were collected at 3 b-values, 500, 1000, and 2500 s/mm^2^, with 32, 64, and 128 directions, respectively. Twenty-six non-diffusion-weighted (b = 0) measurements were interleaved with the diffusion-weighted measurements. Two diffusion-weighted images with opposite phase-encoding at all directions were acquired for each participant.

### 2.3 Preprocessing

#### 2.3.1 T1w

T1w images were preprocessed using the QSIprep-1.0.0rc1 (Cieslak et al., 2021) anatomical pipeline. In brief, the T1w image was reoriented into AC-PC alignment via a 6-DOF transform extracted from a full affine registration to the MNI152NLin2009cAsym template (Fonov et al., 2009). A nonlinear registration to the T1w image from AC-PC space was estimated via symmetric nonlinear registration (SyN) using antsRegistration (ANTs-2.4.3; Avants et al., 2008). Brain extraction was performed on the T1w image using SynthStrip (Hoopes et al., 2022), and automated segmentation was performed using SynthSeg (Billot, Greve, et al., 2023; Billot, Magdamo, et al., 2023) from FreeSurfer-7.3.1.

#### 2.3.2 FLAIR

First, FLAIR images were skull-stripped with mri_synthstrip (Hoopes et al., 2022) and corrected for intensity non-uniformity with the N4 algorithm (Tustison et al., 2010) implemented in ANTsPy. Images were then coregistered to the participant’s preprocessed T1w image from Section 2.3.1 through a rigid body registration. FLAIR images were standardized by participants’ white matter voxel intensities based on the individual white matter masks created in Section 2.3.1. Lastly, nonlinear spatial normalization to the MNI152NLin2009cAsym template was performed on the FLAIR images.

#### 2.3.3 Diffusion MRI

Diffusion MRI preprocessing was performed using the QSIprep-1.0.0rc1 (Cieslak et al., 2021) diffusion pipeline, which is based on Nipype-1.9.1 (Gorgolewski et al., 2011). The following steps were adapted from the QSIprep’s boilerplate. Diffusion images were processed with MP-PCA denoising (MRtrix3’s dwidenoise, auto-voxel window; Veraart et al., 2016) and Gibbs unringing (MRtrix3’s mrdegibbs; Kellner et al., 2016). The mean intensity of the dMRI series was adjusted so all the mean intensity of the b = 0 images matched across each separate dMRI scanning sequence. Diffusion images were grouped by their phase-encoded polarity and merged into a single file.

FSL’s eddy was used for head motion correction and Eddy current correction (q-space smoothing factor = 10, 5 iterations, 1000 voxels; Andersson & Sotiropoulos, 2016). Linear first- and second-level models were used to characterize Eddy current related spatial distortion. Q-space coordinates were forcefully assigned to shells, and field offset was attempted to be separated from participant movement. Shells were aligned post-eddy correction. Eddy’s outlier replacement was run by grouping data by slice, only including values from slices determined to contain at least 250 intracerebral voxels. Groups deviating by more than 4 standard deviations from the prediction had their data replaced with imputed values.

Data were collected with reversed phase-encode blips, resulting in pairs of images with distortions going in opposite directions, so b = 0 images were extracted from each to be used for diffeomorphic registration (Irfanoglu et al., 2015). FSL’s TOPUP (Andersson et al., 2003) was used to estimate a susceptibility-induced off-resonance field based on b = 0 images extracted from multiple dMRI series with reversed phase encoding directions. The TOPUP-estimated fieldmap was incorporated into the Eddy current and head motion correction interpolation. Final interpolation was performed using the jac method. dMRI time series were resampled to ACPC, generating a preprocessed dMRI run in ACPC space with 2.2×2.2×2.2 mm^3^ isotropic voxels. Lastly, B1 field inhomogeneity correction was applied to the resampled image (MRtrix3’s dwibiascorrect with N4 algorithm; Tustison et al., 2010).

### 2.4 Fazekas scores

Fazekas scores are a visual assessment score of WMH burden in the brain (Fazekas et al., 1987). Subscores range from 0 (absence of WMH) to 3 (abundant WMH) and are assessed for periventricular and deep white matter hyperintensity separately. A final Fazekas score is assessed as the total of the periventricular and deep Fazekas scores (range of 0–6). Fazekas scores were rated for each participant’s FLAIR image by one of two board-certified radiologists (authors J.B.A. and P.C.).

### 2.5 FLAIR white matter hyperintensity segmentation and categorization

We used a convolutional neural network (Hypermapper; Mojiri Forooshani et al., 2022) to automatically segment WMH voxels from FLAIR images. HyperMapper generated probabilistic voxel-wise maps of WMH from the spatially normalized T1w, T2w, and FLAIR images. Voxels with probabilities ≥0.5 were defined as WMH voxels. Separate WMH regions of interest (ROIs) were defined as contiguous voxels with scikit-image (Pedregosa et al., 2011). The WMH ROIs were transformed from the MNI152NLin2009cAsym template space to the space of the individuals and used for the remaining analyses.

Next, we classified the WMH ROIs based on their location in the white matter. WMH ROIs were categorized as periventricular if the ROI was adjacent to the lateral ventricles—excluding voxels immediately adjacent (within 1 mm) to the ventricles to avoid partial volume effects—or as deep otherwise. The remaining white matter volume was categorized as normal-appearing white matter (NAWM).

### 2.6 Creating a single-shell diffusion dataset

We created a single-shell diffusion-weighted dataset by subsampling the preprocessed multi-shell diffusion-weighted measurements to only include non-diffusion (b = 0, 26 measurements) and b-value of 1000 s/mm^2^ (64 directions) images. We define the complete multi-shell dataset as the “full multi-shell” data. Correspondingly, we define the b = 1000 subsampled dataset as the “full single-shell” dataset.

### 2.7 Free water elimination

Free water is modeled in each voxel in the white matter using a mixture model (Pierpaoli & Jones, 2004):

$$S\left(\theta ,b\right)=S_{0}\left(1-f\right)e^{-b\theta Q\theta^{T}}+S_{0}fe^{-bD_{iso}}$$

where S(θ,b) is the signal measured when a gradient is applied in direction θ (encoded as a unit vector) with diffusion-weighting b, $S_{0}$ is the signal measured when no diffusion-weighting is applied, Q is a symmetric matrix representing a diffusion tensor, which has six free parameters, $D_{iso}$ = 3 mm^2^/s is the diffusivity of free water at body temperature, and f is a free parameter for the free water fraction in the voxel. This is the free water DTI model (FWDTI).

FWDTI is well-posed for multi-shell acquisitions and can be fit accurately using non-linear least squares optimization (Hoy et al., 2014). We used the implementation previously described in Henriques et al. (2017), which is included in the open-source DIPY software library (Garyfallidis et al., 2014). In single-shell acquisitions, the model is ill-posed, and a spatially-regularized gradient descent algorithm is used to fit the model (Pasternak et al., 2009). We used the software implementation from Golub et al. (2021). After the FWDTI model is fit, the free water component can be eliminated:

$$S_{fwe}\left(\theta ,b\right)=S\left(\theta ,b\right)-S_{0}fe^{-bD_{iso}}$$

where FWE stands for “free water eliminated” and $S_{fwe}$ is the signal with the free water component removed. While the DTI model itself does not accurately represent the orientations of crossing fibers, it does accurately represent the diffusion signal (Rokem et al., 2015). However, the FWE signal is the residual from the removal of a DTI-based model, not a DTI-based signal itself, so it can retain accurate information about orientations of crossing fibers.

### 2.8 Multi-shell multi-tissue (MSMT) constrained spherical deconvolution (CSD)

Multi-shell multi-tissue (MSMT) techniques reconstruct fiber orientation distribution functions (fODFs) based on signal contributions from three tissue types: cerebrospinal fluid (CSF), gray matter (GM), and white matter (WM). By estimating the fractional contributions of each tissue type within a voxel, MSMT reduces signal contamination from CSF and GM and separates WM-specific fODFs (Jeurissen et al., 2019).

We used the MSMT constrained spherical deconvolution (CSD) implementation provided in the DIPY software library (Garyfallidis et al., 2014). First, tissue-specific response functions for CSF, GM, and WM were estimated for each b-value shell using data from the lowest shell. This step produced a coarse mask (mask_for_response_msmt) used to estimate tissue-specific response functions. The response functions for CSF and GM were restricted to be isotropic, while the WM response function was modeled using spherical harmonics of order lmax = 8. Using these estimated response functions, whole-brain MSMT CSD was applied to the full multi-shell diffusion acquisition. We also applied DIPY’s MSMT CSD pipeline to a single-shell dataset, following the same procedure described above. In the single-shell setting, MSMT is ill-posed, and it only estimates response functions for CSF and WM.

### 2.9 Within-participant split-half datasets

To examine the impact of free water elimination on reliability, we created within-participant split-half datasets from the full multi-shell and single-shell datasets. The dMRI images were randomly and evenly split into two at each b-value.

For the multi-shell dataset, this resulted in two dMRI images with 3 b-values, 500, 1000, 2500 s/mm^2^, with 16, 32, and 64 directions, respectively, and 13 non-diffusion-weighted (b = 0) measurements for each participant. We define this dataset as the “split-half multi-shell” dataset.

For the single-shell dataset, this resulted in two dMRI images with 32 directions at b-value of 1000 s/mm^2^ and 13 non-diffusion-weighted (b = 0) measurements for each participant. We define this dataset as the “split-half single-shell” dataset. Subsequent analyses were performed on the full and split-half, multi-shell and single-shell datasets.

### 2.10 Tractometry

We used pyAFQ-1.3.6 (Kruper et al., 2021, 2025), an open-source automated tractometry pipeline. The methods were previously described in detail in Kruper et al.; briefly, we used methods from DIPY (Garyfallidis et al., 2014) to perform CSD (Tournier et al., 2007) using all of the b-value shells in the dMRI acquisition, and we used the fODFs in every voxel as cues for probabilistic tractography, with 8 streamlines seeded in every voxel in the white matter. Every individual’s brain was aligned to the MNI152NLin2009cAsym template using non-linear registration, implemented in DIPY. For every one of 28 major white matter pathways, inclusion and exclusion ROIs defined in the space of the template were back-transformed into the space of the individual and used to select streamlines that passed through inclusion and did not pass through exclusion ROIs. Each streamline was resampled to 100 points. Streamlines that deviated significantly (>5 standard deviations) from the median trajectory were removed from each tract.

To characterize microstructural tissue properties within each voxel of the white matter, we used the diffusional kurtosis imaging model (DKI; Jensen et al., 2005). DKI was fit on multi-shell datasets using the Diffusion Imaging in Python (DIPY, version 1.9.0) software library (Garyfallidis et al., 2014; Henriques et al., 2017). We derived fractional anisotropy (DKI-FA), mean diffusivity (DKI-MD), and mean diffusional kurtosis (DKI-MK). In addition, the parameters of the DKI model were used to fit the biophysically-motivated White Matter Tract Integrity (WMTI) model (Fieremans et al., 2011), from which we derived a metric of axonal water fraction (DKI-AWF). In single-shell data, we fit the diffusion tensor imaging (DTI) model (Basser et al., 1994) using DIPY, and we derived fractional anisotropy (DTI-FA) and mean diffusivity (DTI-MD) metrics. Importantly, based on recent research that demonstrates the pitfalls of using metrics based on the FWDTI model (Correia et al., 2024; Golub et al., 2021), tract tissue properties were always derived from the Original, and not from the FWE signal.

Tract profiles of tissue properties were derived by weighting the contribution of each streamline at the voxel that corresponds with each node, inversely weighted by that node’s distance from the median node in that position.

### 2.11 Reliability estimates

**Fig. 1.**
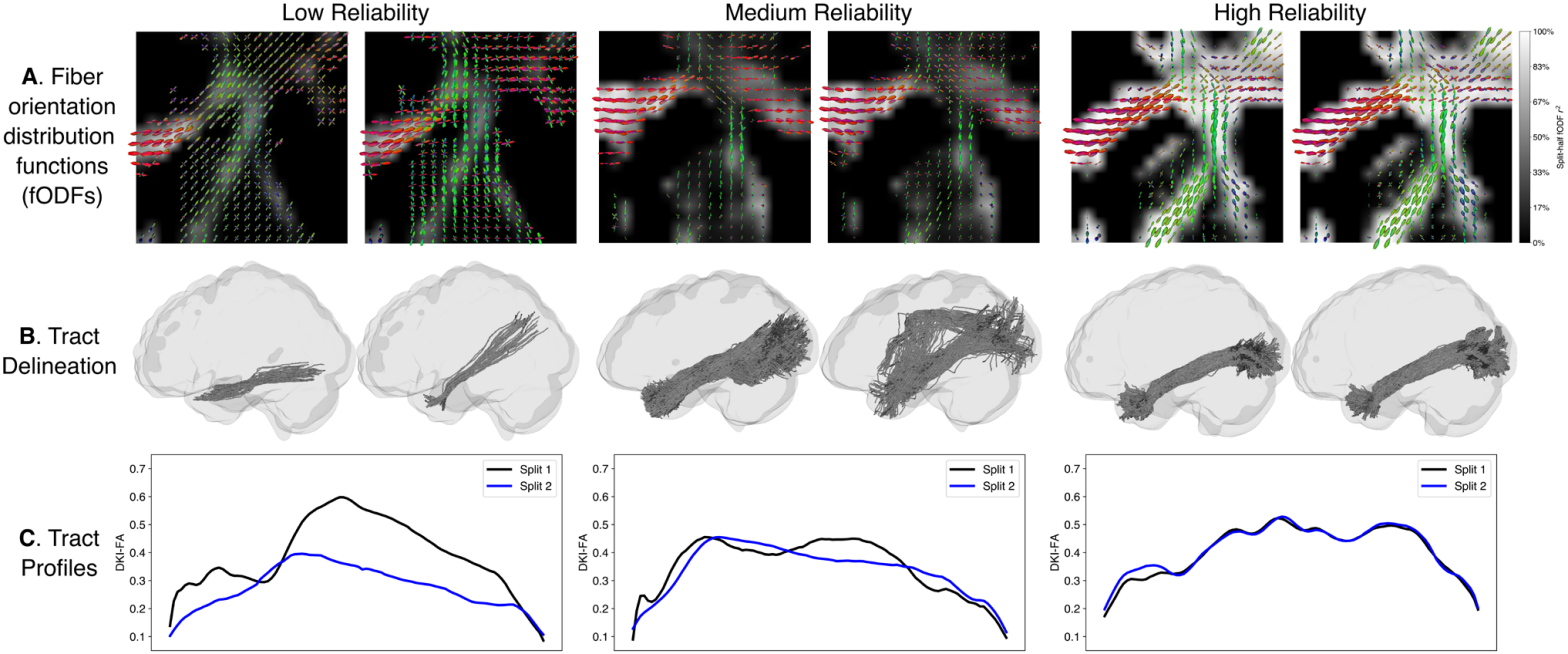
Reliability assessment of the tractometry pipeline. Split-half reliability increases from left to right (low, medium, high) for example participants. (A) Fiber orientation distribution functions (fODFs) reliability was assessed with $r^{2}$ across splits, (B) tract delineations were assessed with weighted dice coefficients, and (C) tract profiles (of DKI-FA) were assessed with intraclass correlation coefficient (ICC).

The tractometry pipeline is composed of several different steps. To assess the effects of FWE on reliability at each one of these steps, both the split-half multi-shell and single-shell datasets were submitted to the same steps, without modification, with MSMT-derived WM fODFs, or with the preceding FWE procedure, and reliability was assessed across the two halves at each step, in each one of these cases. To illustrate differences in reliability across methods, we visualized the fiber orientation distribution functions (fODFs; Fig. 1A), tract delineations (Fig. 1B), and tract profiles (Fig. 1C) for representative cases with low, medium, and high split-half reliability.

#### 2.11.1 Fiber orientation distribution functions (fODF)

We used the CSD model (Tournier et al., 2007), implemented in DIPY, to estimate fODFs. To assess reliability, each fODF was discretized to 362 uniformly-distributed positions on the sphere. Pearson’s correlation coefficient was obtained by correlating the discretized fODF for each voxel across split-halves. FODF reliability was assessed in terms of explained variance, quantified as the squared Pearson’s correlation coefficient, denoted as $r^{2}$. Percent change in fODF reliability, relative to the Original data, was calculated as

$$\frac{r^{2}-r_{Original}^{2}}{0.5\;(r^{2}+r_{Original}^{2})}\times 100\%$$

where $r^{2}$ is from either the FWE or MSMT method.

#### 2.11.2 Tract delineation

Tract density maps were created by counting the number of streamlines that pass through each voxel for each participant, tract, dataset, and tractography method. The Dice coefficient, weighted by the streamline visitation map (Cousineau et al., 2017), was computed across the tract density maps of each split-half.

#### 2.11.3 Tract profiles

The intraclass correlation coefficient (ICC) was calculated across split-halves for each tract, dMRI metric, dataset, and tractography method. We treated each split-half as an independent rater and used the ICC(2,1) implementation in pingouin-0.5.4 (Vallat, 2018).

### 2.12 Inferences from tractometry

The tract profiles for each participant were used as the features for Fazekas scores prediction. We used DKI-AWF, DKI-FA, DKI-MD, and DKI-MK for the full multi-shell dataset, and we used DTI-FA and DTI-MD for the full single-shell dataset. The tract profiles were z-score standardized and PCA transformed prior to model fitting (separately for the test and training data). Both models used to predict Fazekas scores were implemented in scikit-learn (version 1.4.2; Pedregosa et al., 2011).

#### 2.12.1 Fazekas score prediction

We implemented a 5-fold logistic regression to predict Fazekas scores using participant tract profiles, stratified into folds to include Fazekas scores that are proportional to the sample as a whole. The logistic regression model was trained using the scikit-learn LogisticRegressionCV (Pedregosa et al., 2011), which used L2 regularization and performed a 3-fold internal cross-validation to tune the regularization parameter. The logistic regression was configured for multiclass classification and adjusted imbalanced Fazekas scores frequencies by adjusting class weights inversely proportional to their frequencies in the dataset. The cross-validation process was repeated 1,000 times to account for variability from unrepresentative splits due to the uneven Fazekas scores distribution.

## 3 Results

### 3.1 Free water elimination improves reliability of tractometry

Reliability was assessed across both split-half multi-shell and single-shell datasets to evaluate how FWE and MSMT impact reliability at each step in the pipeline.

#### 3.1.1 Fiber orientation distribution functions (fODFs)

**Fig. 2.**
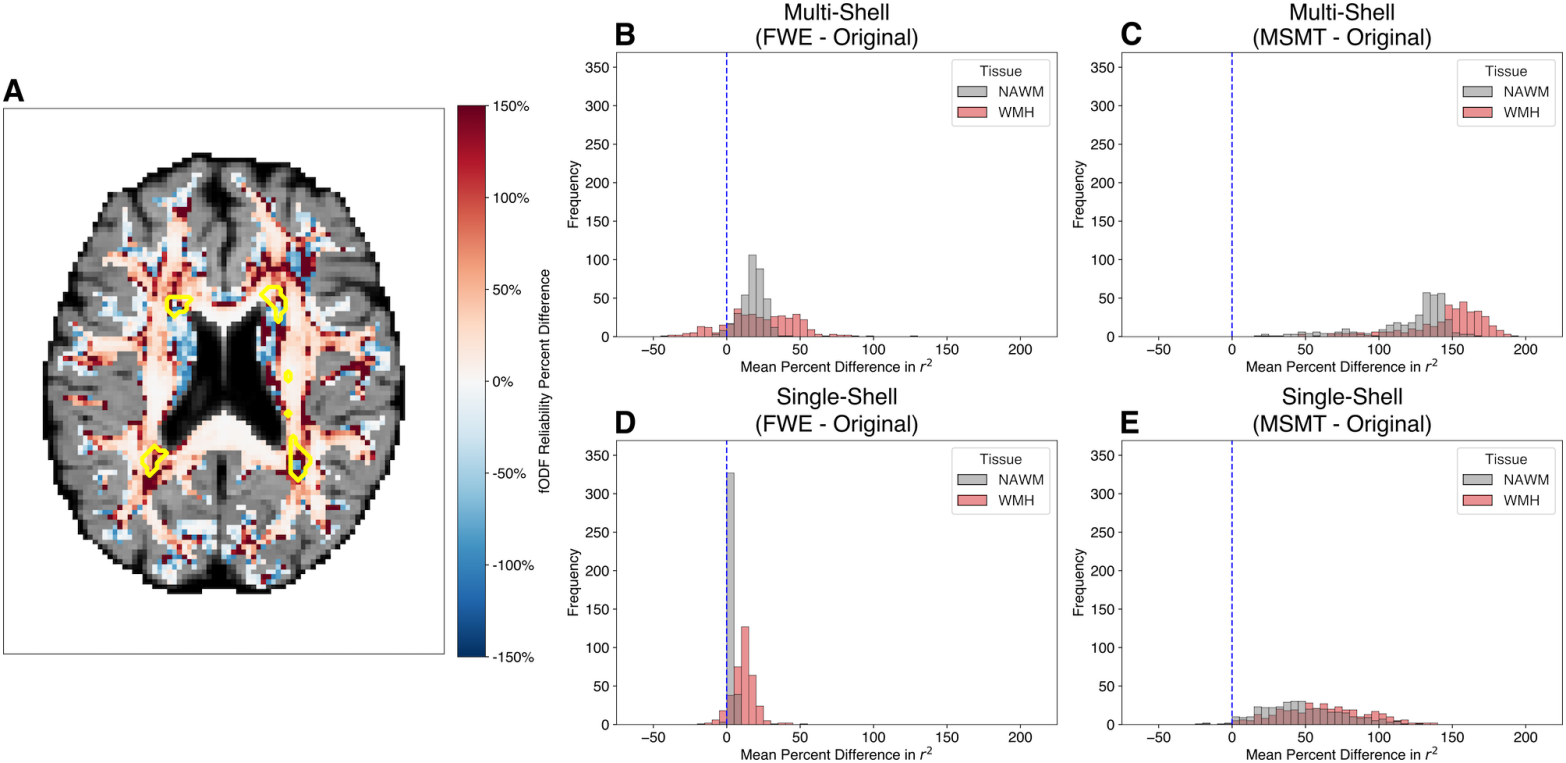
Fiber orientation distribution functions (fODFs) split-half reliability. (A) Percent difference (FWE - Original) / Original in fODF reliability of an example participant. The yellow outlines represent regions of white matter hyperintensities. Histogram of (B) FWE and (C) MSMT multi-shell split-half fODF reliability percent difference in normal appearing white matter (NAWM) and white matter hyperintensities (WMH). Histogram of (D) FWE and (E) MSMT single-shell split-half fODF reliability percent difference in NAWM and WMH.

FODF reliability was quantified as the explained variance (Pearson correlation squared) between the fODF in the two split-halves, discretized to 362 points on the sphere, calculated in each voxel. Because we hypothesized that regions of leukoaraiosis would be particularly susceptible to confounds because of CSF intrusion, we split our analysis to voxels in WMH and voxels in NAWM (Fig. 2A shows one participant’s data).

FWE fODF reliability was 17.79% (±0.42% SEM; Wilcoxon signed-rank test: z = 16.54, p < 0.0001) higher in NAWM and 23.38% (±1.28% SEM; Wilcoxon signed-rank test: z = 13.55, p < 0.0001) higher in WMH in multi-shell data (Fig. 2B). Similarly, FWE fODF reliability was 3.41% (±11.37% SEM; Wilcoxon signed-rank test: z = 16.64, p < 0.0001) higher in NAWM and 11.37% (±0.42% SEM; Wilcoxon signed-rank test: z = 15.80, p < 0.0001) higher in WMH in the single-shell data (Fig. 2D).

MSMT fODF reliability was 120.08% (±1.55% SEM; Wilcoxon signed-rank test: z = 16.65, p < 0.0001) higher in NAWM and 144.46% (±1.56% SEM; Wilcoxon signed-rank test: z = 16.58, p < 0.0001) higher in WMH in multi-shell data (Fig. 2C). Similarly, MSMT fODF reliability was 47.95% (±1.41% SEM; Wilcoxon signed-rank test: z = 16.60, p < 0.0001) higher in NAWM and 61.60% (±1.62% SEM; Wilcoxon signed-rank test: z = 16.42, p < 0.0001) higher in WMH in the single-shell data (Fig. 2E).

**Fig. 3.**
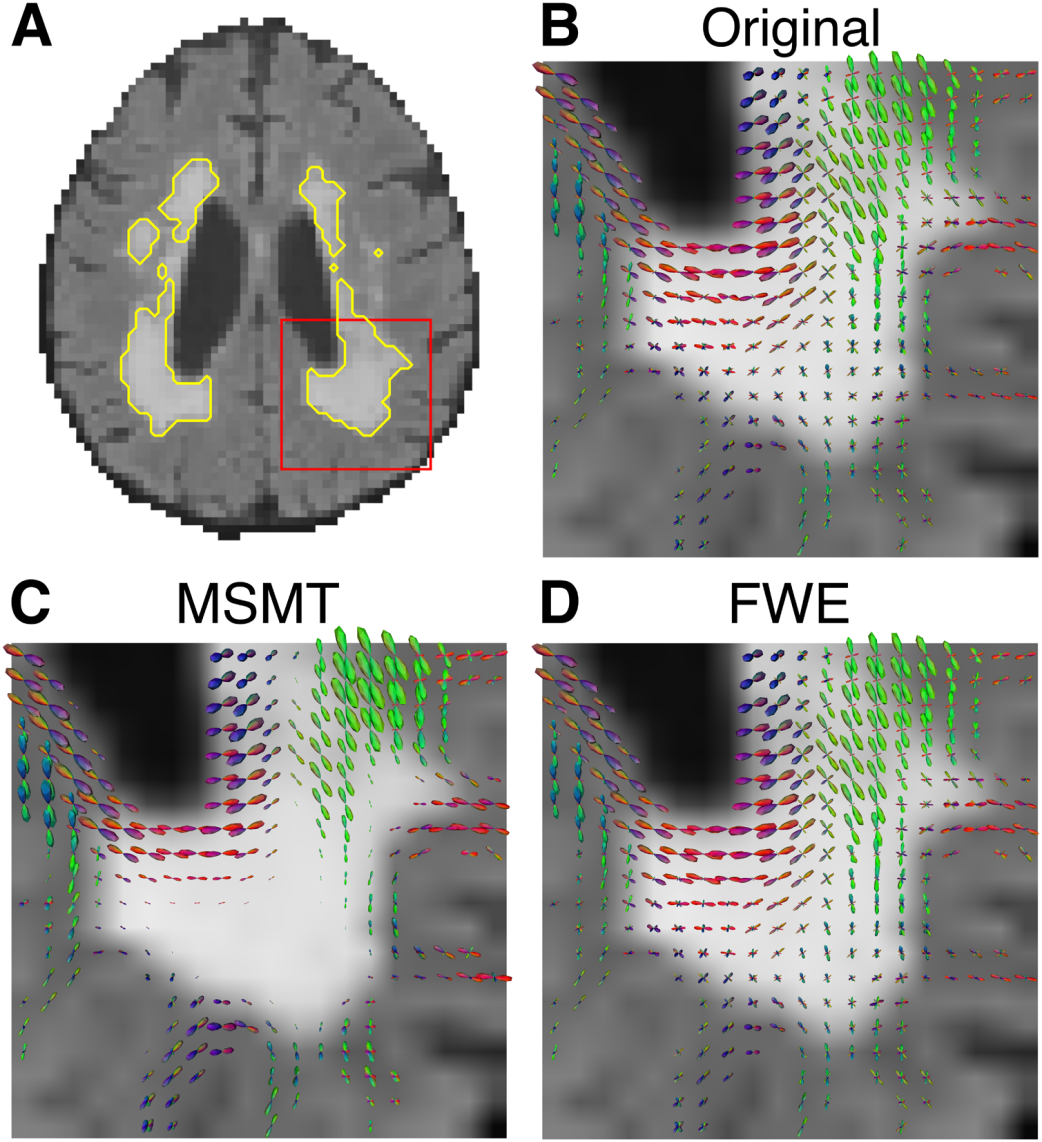
Fiber orientation distribution functions (fODFs) in white matter hyperintensities (WMH) for an example participant. (A) Axial FLAIR image with WMH regions outlined in yellow. The red box indicates the region enlarged in panels (B–D). (B) fODFs derived from the Original diffusion data, (C) fODFs after MSMT processing, and (D) fODFs after FWE processing are shown for the zoomed-in region.

Overall, while MSMT demonstrated increased fODF reliability throughout the white matter, it also showed reduced directional signal within WMH regions, particularly those adjacent to the ventricles (Fig. 3C). In these regions, the MSMT fODFs appear attenuated, indicating that the model attributed a larger portion of the diffusion signal to isotropic components. Whereas the FWE model retained anisotropic fODFs within WMH regions, even in the presence of elevated free water (Fig. 3D). Corresponding maps of tissue compartment fractions for the same participant from the MSMT and FWE pipelines are provided in Supplemental Figure 1.

#### 3.1.2 Tract delineation

Tract delineation reliability was quantified as the difference in FWE or MSMT from the Original weighted Dice coefficient between tract density maps from each split-half of the data. On average, the weighted Dice coefficients across tracts were 0.56 (±0.23 s.d.) for the multi-shell dataset with FWE and 0.85 (±0.17 s.d.) for MSMT processing, compared to an average 0.54 (±0.23 s.d.) for the Original tracts. For the single-shell dataset, FWE tracts had an average weighted Dice coefficient of 0.81 (±0.17 s.d.), MSMT tracts had an average of 0.81 (±0.18 s.d.), while the Original tracts had an average of 0.78 (±0.20 s.d.).

**Fig. 4.**
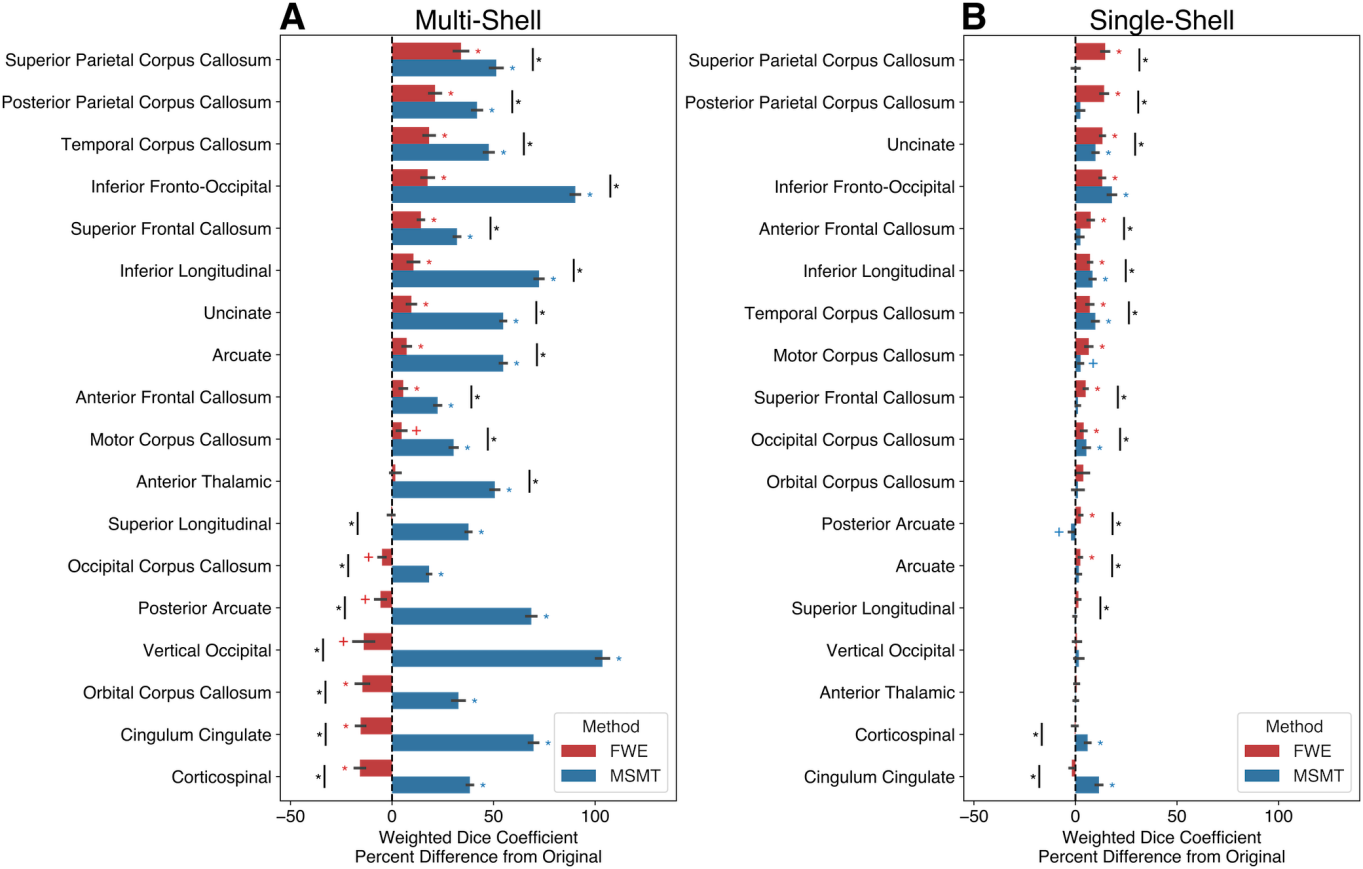
Tract reliability weighted dice coefficient differences. Weighted dice coefficient differences are shown for the split-half (A) multi-shell and (B) single-shell datasets collapsed across hemispheres. The difference was calculated as FWE (red) or MSMT (blue) - Original. Error bars represent ±1 SEM. Asterisks (Bonferroni corrected) and crosses (without Bonferroni correction) represent tracts with weighted Dice coefficient differences that were significantly different from 0 or across methods.

For the split-half multi-shell dataset, FWE tracts had larger weighted Dice coefficients for all tracts except for the occipital corpus callosum, posterior arcuate, vertical occipital fasciculus, orbital corpus callosum, cingulate section of the cingulum bundles, and corticospinal tract, as compared to the Original tracts. MSMT tracts had larger weighted dice coefficients for all tracts as compared to the Original tracts (Fig. 4A). A smaller, yet consistent increase in reliability with FWE and MSMT was assessed in the split-half single-shell data (Fig. 4B).

#### 3.1.3 Tract profiles

Tract profile reliability was quantified as the intraclass correlation coefficient (ICC) for each tract across split-halves. For the multi-shell dataset, profile ICC across tracts and dMRI metrics ranged from 0.38 to 0.85 for the FWE processed tracts, from 0.81 to 0.95 for the MSMT tracts, and from 0.40 to 0.86 for the Original tracts. For the single-shell dataset, profile ICC across tracts and metrics ranged from 0.81 to 0.95 with FWE, from 0.79 to 0.94 with MSMT, and from 0.77 to 0.93 with Original tract processing. These ICC values are consistent with previously reported tract profile reliability estimates (Kruper et al., 2021).

**Fig. 5.**
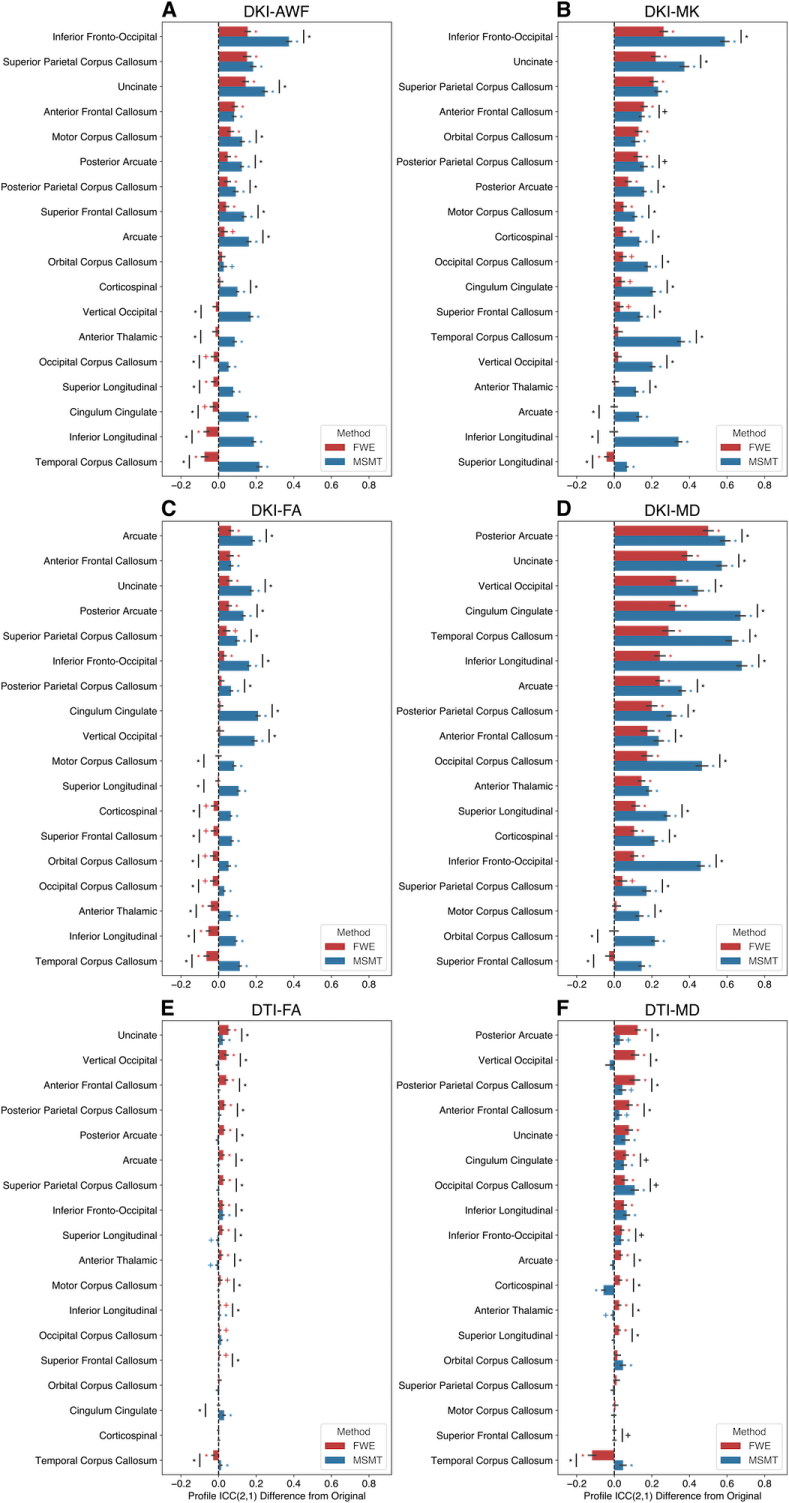
Differences in tract profile ICC(2,1). Tract profile ICC(2,1) differences are shown for split-half multi-shell (A) DKI-AWF, (B) DKI-MD, (C) DKI-FA, and (D) DKI-MD. Tract profile ICC(2,1) differences are shown split-half single-shell (E) DTI-FA and (F) DTI-MD. The difference was calculated as FWE (red) or MSMT (blue) - Original. Error bars represent ±1 SEM. Asterisks (Bonferroni corrected) and crosses (without Bonferroni correction) represent tracts with profile ICC(2,1) differences that were significantly different from 0 or across methods.

The difference in tract profile ICC was used to compare tract profile reliability with FWE or MSMT from processing without either. For the multi-shell dataset, FWE and MSMT tract profile ICCs for all DKI measures were greater than or the same as the Original tract profile ICC values (Fig. 5 A, B, C, D). A similar, but substantially smaller effect was observed in the single-shell dataset (Fig. 5 E, F).

### 3.2 FWE increases tract yield

To assess tract reconstruction performance across preprocessing methods, we evaluated tract yield. A successfully segmented tract was defined as a tract with at least 10 streamlines.

**Fig. 6.**
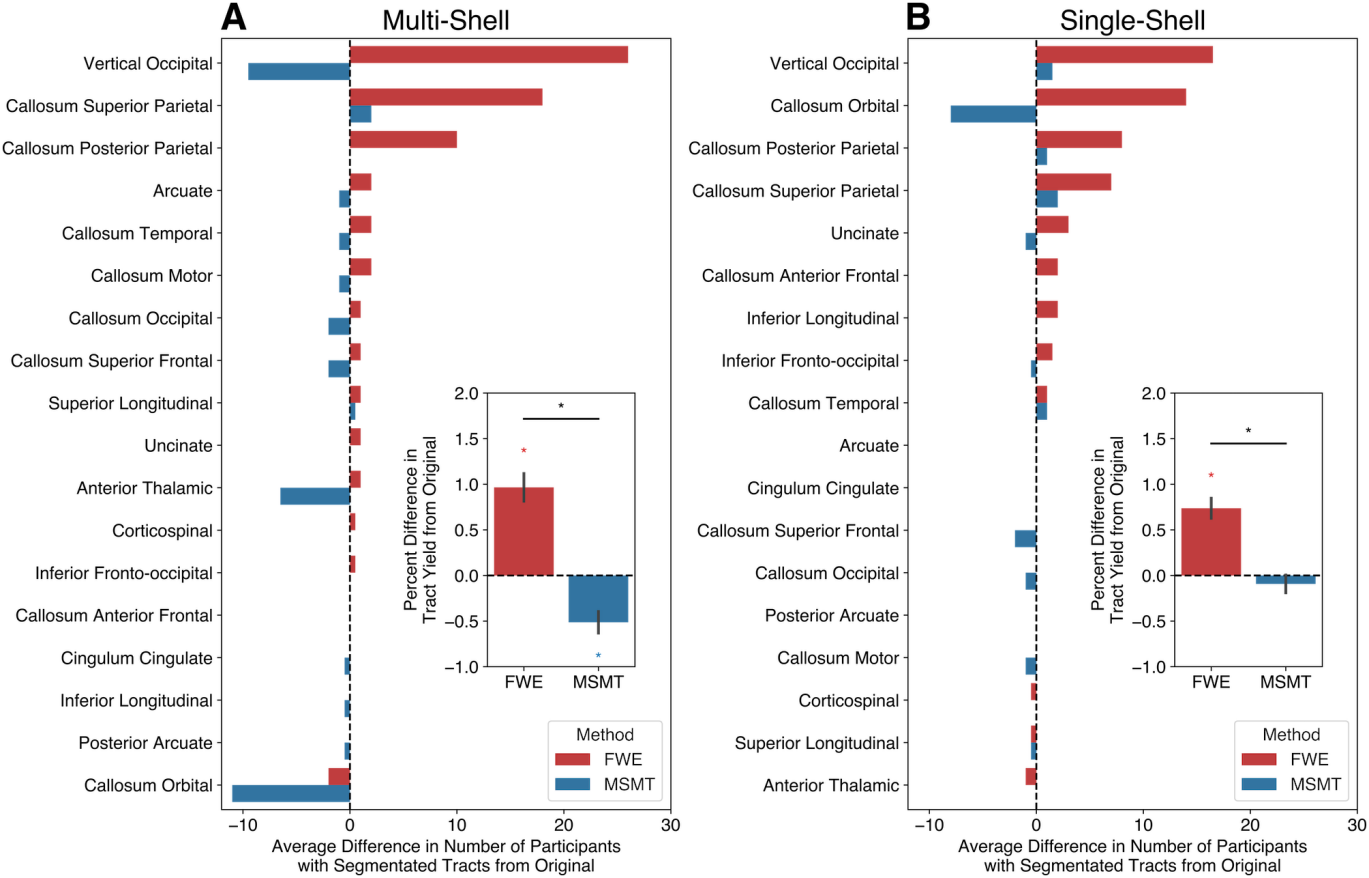
Tract yield differences. Tract yield difference in the number of participants with successfully segmented tracts between FWE (red) or MSMT (blue) from the Original processing for (A) multi-shell and (B) single-shell datasets. Insets show overall percent differences. Asterisks represent tract yield differences that were significantly different (Bonferroni corrected) from 0 or across methods.

We quantified tract yield by calculating the number of tracts successfully segmented for each participant. We then compared the number of segmented tracts following FWE and MSMT processing to the Original processing method. In both the multi-shell and single-shell datasets, FWE processing resulted in a higher tract yield across most tracts, and MSMT processing generally produced either a comparable or lower tract yield relative to the Original processing (Fig. 6A, B).

### 3.3 FWE tracts overlap more with white matter hyperintensities

To better understand the spatial relationship between processing methods and WMH, we calculated the weighted Dice coefficients between each tract and WMH regions. Tract overlap with WMH was quantified as the weighted Dice coefficient between tract streamlines and any WMH ROI. On average, the weighted Dice coefficients across tracts were 0.020 (±0.037 s.d.) for the multi-shell dataset with FWE and 0.015 (±0.031 s.d.) for MSMT processing, compared to an average 0.015 (±0.031 s.d.) for the Original tracts. For the single-shell dataset, FWE tracts had an average weighted Dice coefficient of 0.021 (±0.040 s.d.), MSMT tracts had an average of 0.017 (±0.033 s.d.), while the Original tracts had an average of 0.017 (±0.033 s.d.).

**Fig. 7.**
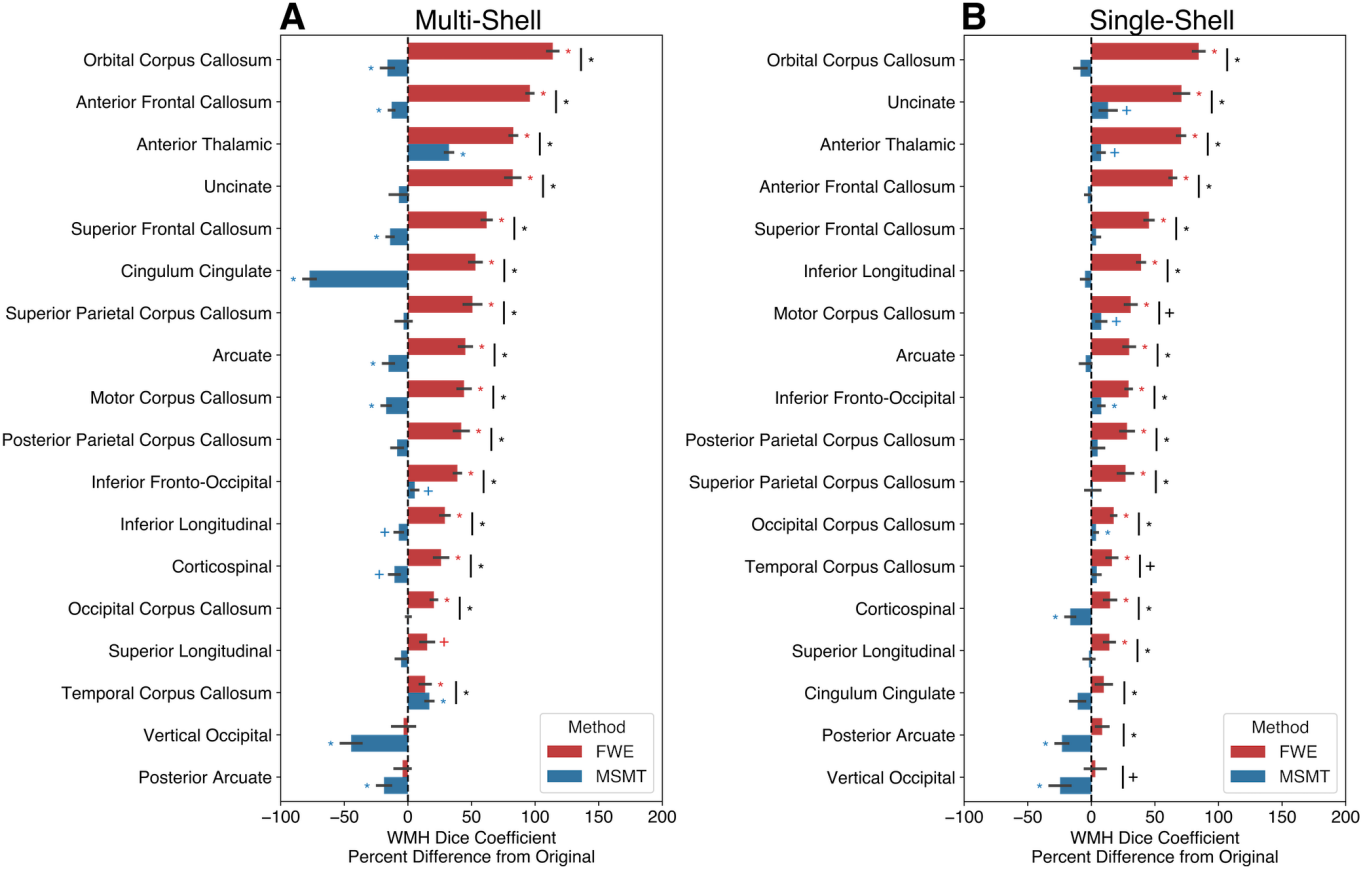
Tract and white matter hyperintensity weighted dice coefficient differences. Weighted dice coefficient differences are shown for the (A) multi-shell and (B) single-shell datasets collapsed across hemispheres. The difference was calculated as FWE (red) or MSMT (blue) - Original. Error bars represent ±1 SEM. Asterisks (Bonferroni corrected), and crosses (without Bonferroni correction) represent tracts with WMH Dice coefficient differences that were significantly different from 0 or across methods.

We calculated the percent difference in tract overlap with WMH ROIs after FWE and MSMT processing, relative to the Original preprocessing. In the multi-shell dataset, FWE-processed tracts exhibited greater overlap with WMH regions for all tracts except the vertical occipital fasciculus and the posterior arcuate; in contrast, MSMT-processed tracts generally showed similar or less overlap as compared to the Original processing, except for the anterior thalamic radiation and the temporal segment of the corpus callosum (Fig. 7A). In the single-shell dataset, FWE processed tracts consistently showed increased overlap with WMH regions across all tracts, whereas MSMT processed tracts displayed similar levels of overlap to those observed in the Original processing (Fig. 7B).

**Fig. 8.**
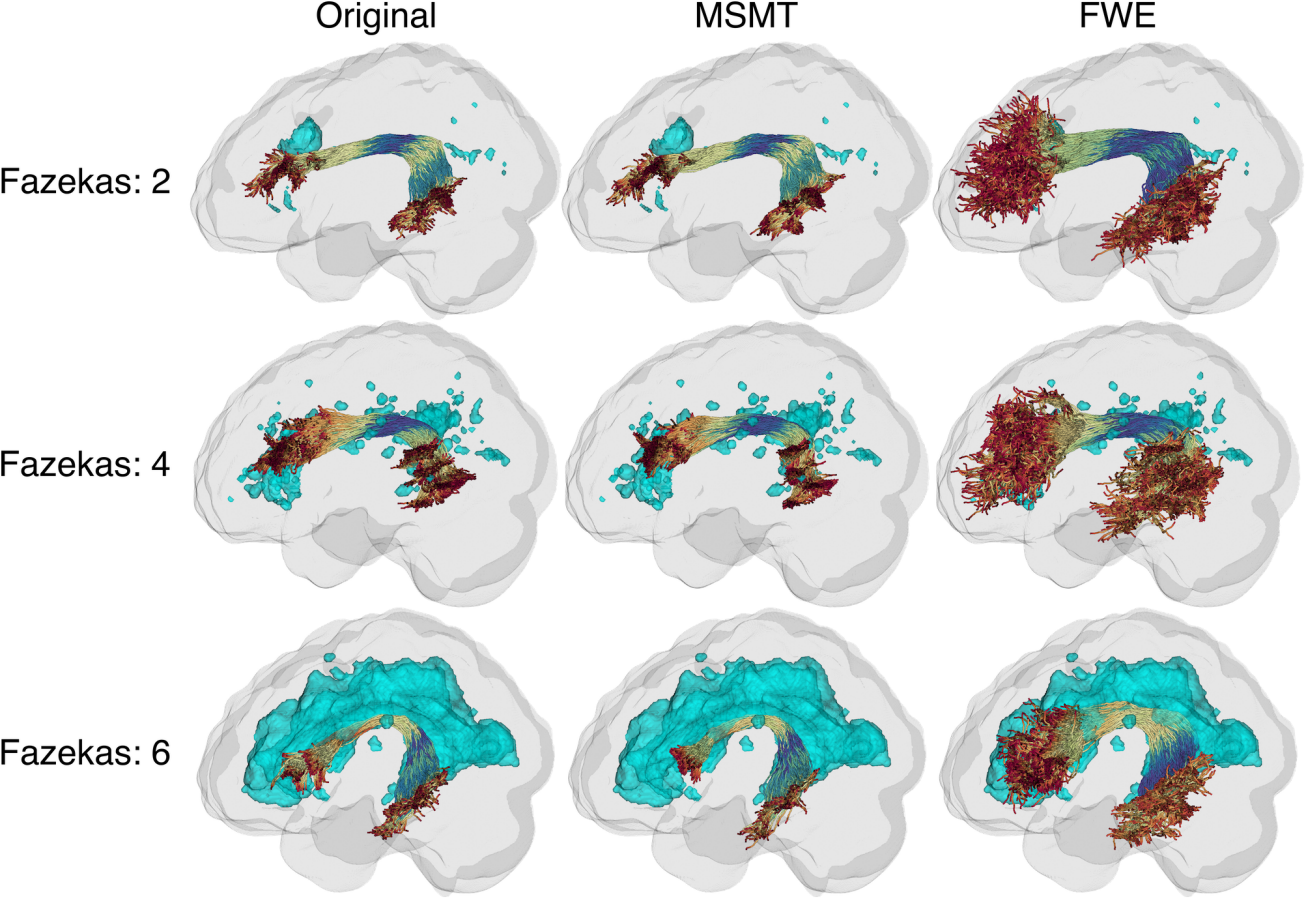
Example of the left arcuate fasciculus by the Fazekas score and processing methods. Cyan regions represent WMH areas. Fazekas scores increase by row, and processing methods differ by column.

We examined the qualitative differences in example tracts produced by each processing pipeline. Across mild (Fazekas 2), moderate (Fazekas 4), and severe (Fazekas 6) WMH burden, we observed the impact on tractography of the left arcuate fasciculus in three participants (Fig. 8; see Supplemental Figs. 16–20 for several more tracts from these individuals). Original (left column) and MSMT (middle column) reconstructions demonstrated progressively lower streamline yield with increasing Fazekas scores. In contrast, FWE (right column) substantially preserved streamline continuity and yield around WMH regions.

**Fig. 9.**
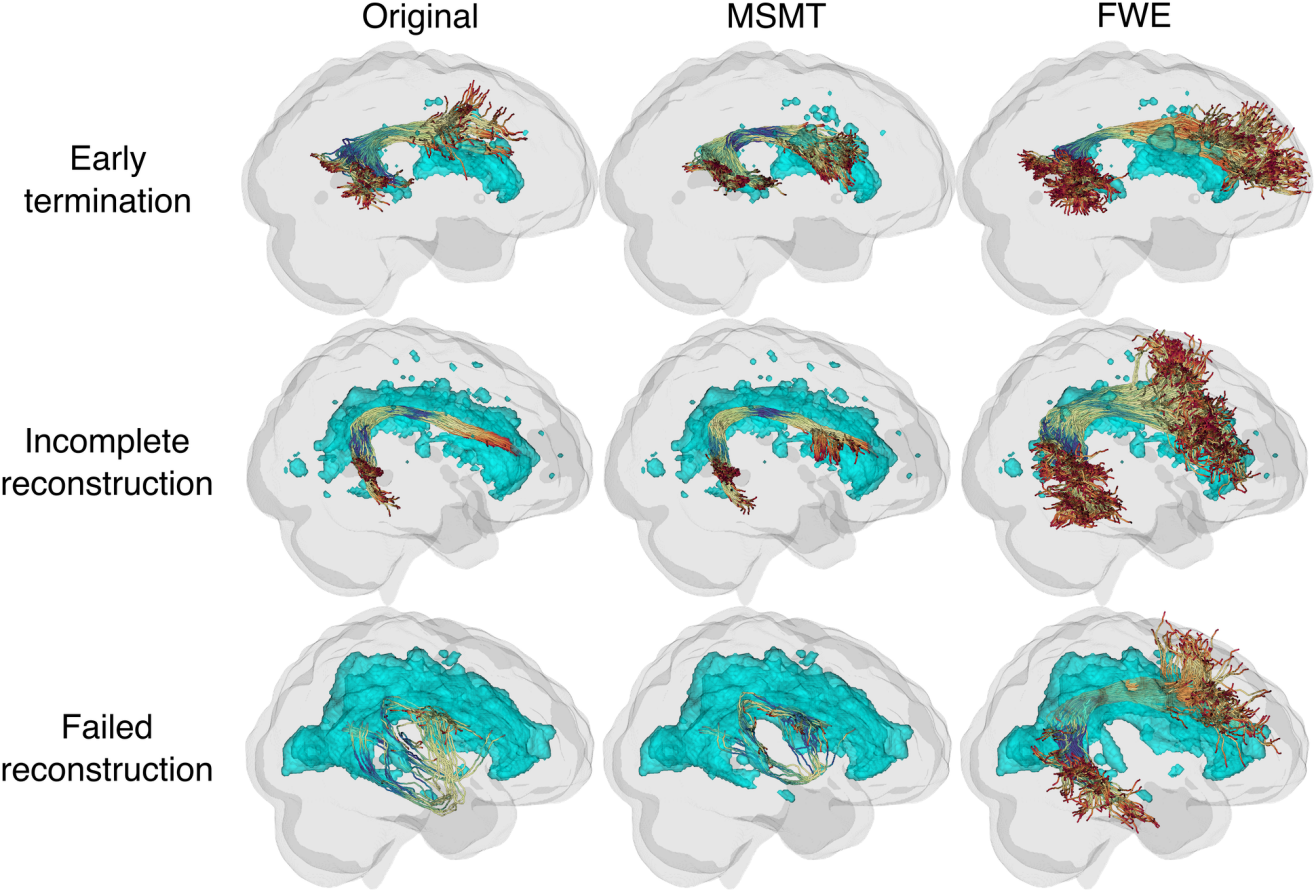
Common tractography issues by processing method. Examples of participants’ right arcuate fasciculus are shown. Cyan regions represent WMH areas.

We also examined the tracts to determine common tractography issues associated with WMH burden (Fig. 9). In cases with early termination (Fig. 9, top row), the Original and MSMT reconstructions prematurely terminated near WMH regions, whereas FWE maintained tract continuity through these regions. For incomplete reconstructions (Fig. 9, middle row), both the Original and MSMT methods showed reduced streamline yield, particularly in WMH adjacent areas, while FWE restored fuller tract coverage. In the most severe case of failed reconstructions (Fig. 9, bottom row), the Original and MSMT methods produced anatomically implausible tracts. In contrast, FWE achieved a plausible and anatomically consistent reconstruction despite extensive WMH burden.

**Fig. 10.**
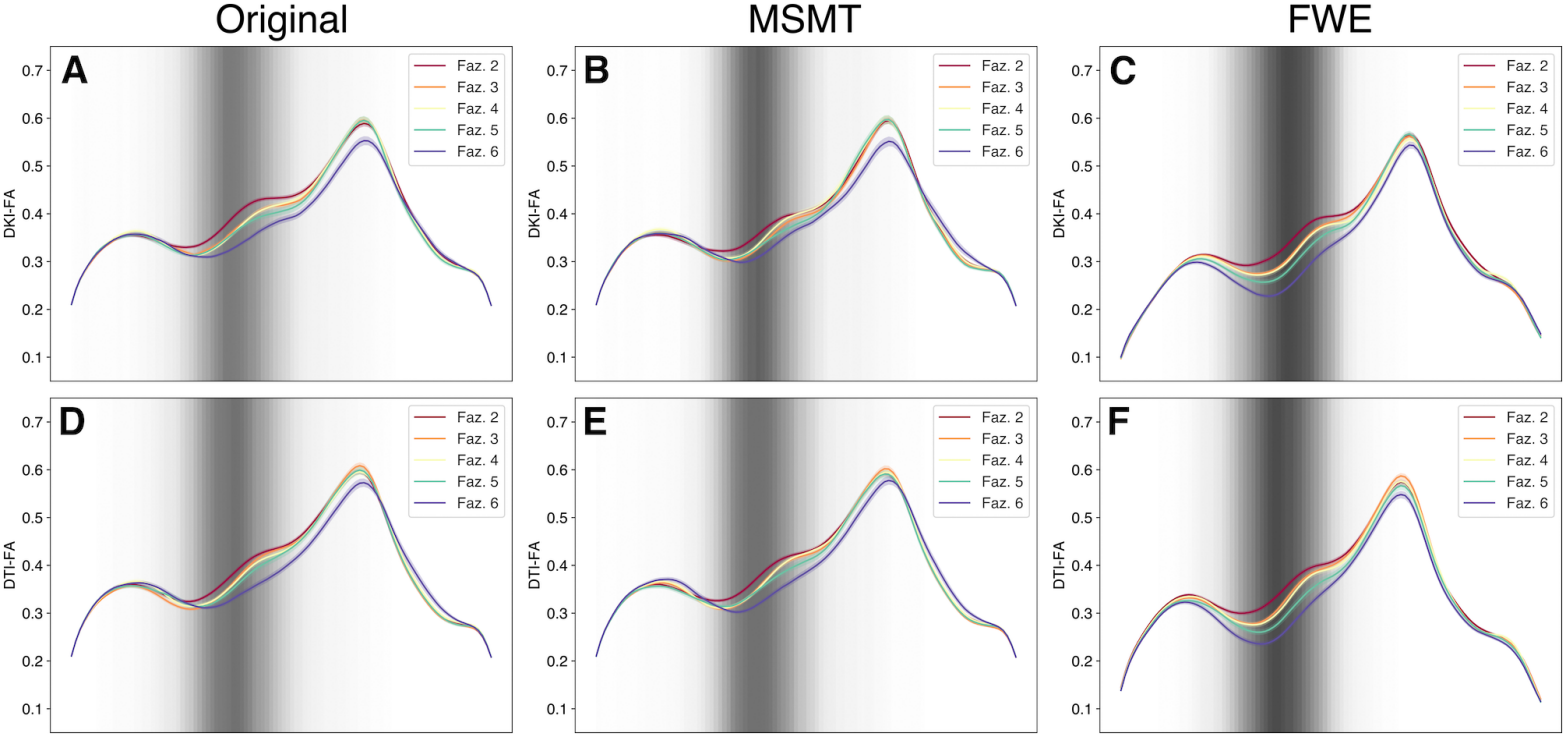
Left anterior thalamic radiation profiles by Fazekas scores. Tract profiles of multi-shell DKI-FA of the (A) Original, (B) MSMT, and (C) FWE are shown in the top row. Tract profiles of single-shell DTI-FA of the (D) Original, (E) MSMT, and (F) FWE are shown in the second row. The line colors correspond to Fazekas scores, and the ribbon width represents ±1 SEM. The background intensity corresponds to the spatial location of WMH overlap across the sample.

The differences in tracts by processing methods are also reflected in the tract profiles of the FA in the anterior thalamic radiation, divided by the clinician’s WMH Fazekas scores. While tract profiles of different Fazekas score groups are hard to distinguish in the Original data (Fig. 10, column 1) and MSMT (Fig. 10, column 2), the profiles diverge in FWE data, both in multi-shell data (Fig. 10, upper right) and in single-shell data (Fig. 10, lower right). Figures showing qualitative observations in every tract and metric are provided as Supplemental Materials.

### 3.4 Fazekas score predictions do not differ across tractometry methods

To quantify the qualitative observation from Figure 10, we used a machine-learning approach to assess increase in the information that can be read out from the tract profiles regarding the extent and severity of leukoaraiosis. In this approach, a logistic regression model was fit to the tract profile data, classifying the individual-level Fazekas score. Predictions on held-out data were assessed in a repeated 5-fold cross-validation procedure.

**Fig. 11.**
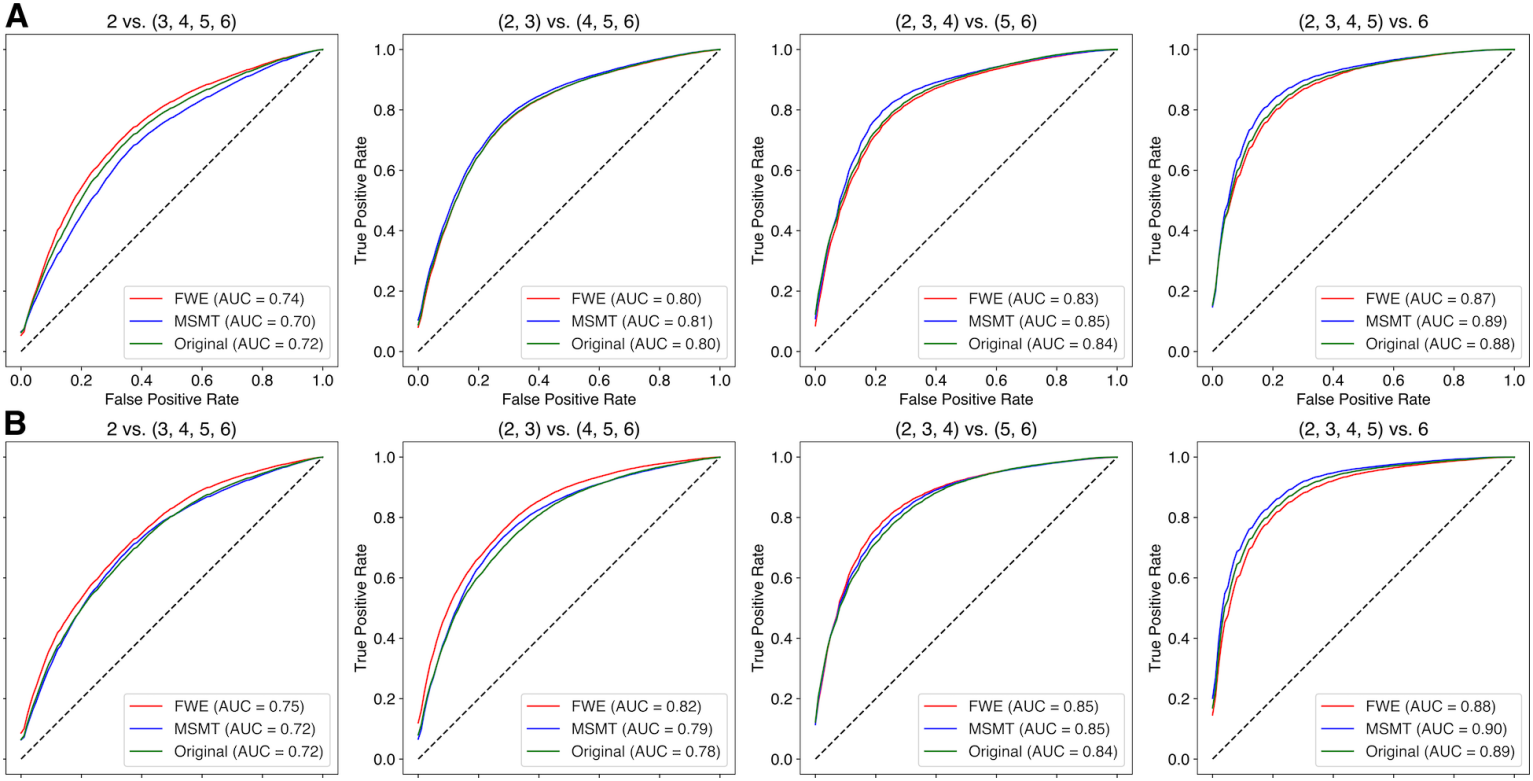
Fazekas score receiver operating characteristic (ROC) curves and area under the curve (AUC) values. (A) The first row shows the ROC curves and AUC for the multi-shell data. (B) The second row shows the ROC curves and AUC for the single-shell data. The line colors correspond to each method, and the ribbon width represents ±1 SEM.

Overall, Fazekas score prediction was accurate across all datasets and methods. For the multi-shell dataset, prediction accuracy for the Original (MAE = 0.93, $R^{2}$ = 0.20), FWE (MAE = 0.92, $R^{2}$ = 0.22), and MSMT (MAE = 0.93, $R^{2}$ = 0.21) was high and was not statistically different from each other. For the single-shell dataset, Fazekas score prediction performed similarly well with the Original (MAE = 0.96, $R^{2}$ = 0.16), FWE (MAE = 0.90, $R^{2}$ = 0.25), and MSMT (MAE = 0.92, $R^{2}$ = 0.21) methods. This was also reflected in high values of area under the curve (AUC) from receiver operating characteristic (ROC) curves. We performed a series of ROC analyses to evaluate the model’s ability to discriminate between progressively higher Fazekas scores (Fig. 11). The analysis began with a binary classification between the lowest Fazekas score and all higher scores. Specifically, we first examined the classification of Fazekas scores of 2 versus scores of 3, 4, 5, and 6. We then incrementally incorporated additional lower Fazekas scores into the lower bin (e.g., {2, 3} vs. {4, 5, 6}). This stepwise approach continued until we compared all lower Fazekas scores {2, 3, 4, 5} against the highest score of 6. AUC performance ranged from 0.7 to 0.9 AUC.

## 4 Control Analyses

Detailed results and figures for all control analyses are provided in the Supplemental Materials.

One potential interpretation of the results is that FWE improves tractography simply by increasing the number of streamlines, effectively lowering the FA threshold at which tractography terminates. To test this, we conducted a control analysis using the Original pipeline with a 50% lowered FA stop threshold. This did not replicate the improvements in reliability observed with FWE and MSMT, suggesting that the observed benefits are not solely due to more permissive streamline propagation.

We also tested whether differences in WM stop masks across methods might account for differences in tractography outcomes. In a second control analysis, we standardized the stop mask across all methods by using a shared WM mask derived from each participant’s QSIPrep output. This controlled for variation in tissue mask definitions that could arise from changes in FA after free water removal. We found that the overall pattern of reliability differences persisted, with FWE and MSMT continuing to outperform the Original pipeline in terms of reliability, albeit with slightly weaker effect sizes.

Finally, we asked whether the advantages of FWE and MSMT tractography generalize to younger populations. Using the Human Connectome Project Young Adult Test-Retest dataset (Glasser et al., 2016), we found that the impact of FWE and MSMT on tractography was minimal in this younger, healthy cohort. This suggests that the benefits of free water modeling are more pronounced in aging populations where extracellular water content and white matter damage are more prevalent.

## 5 Discussion

Tractometry is a powerful tool for studying human brain white matter connections across a range of different research questions, and with potential applications in clinical settings. Here, we compared two approaches for mitigating the effects of free water in aging brains, FWE and MSMT, and demonstrated that both improve the reliability of tractography results in the brains of aging individuals. The most notable improvements were in fODF reliability throughout the white matter, with particularly large effects in WMH regions. These regions, and particularly periventricular WMH regions, are known to have high MD, low FA, and a lower magnetization transfer ratio (Bastin et al., 2009; Maniega et al., 2015), reflecting pathological processes of tissue degeneration, axonal loss, and demyelination. Nevertheless, even while tissue biophysics is significantly altered, there is a residual directional signal in these regions that was highly reproducible across splits of the data once free water effects were accounted for. The increased reliability in fODFs translates into more reliably delineated tracts. Particularly strong effects are observed in callosal bundles and the left uncinate. No effects, or even small detrimental effects are observed in the corticospinal tract and cingulum cingulate bundles. Interestingly, the small detrimental effects observed were no longer present when we standardized the WM stop mask across preprocessing methods. This suggests that these apparent decreases in reliability may have been driven by inconsistencies in tissue segmentation, particularly in how FA-based masks are influenced by free water modeling, rather than by true degradation of tract quality.

While improving reliability is beneficial on its own, it is also important to assess whether such improvements translate into anatomically plausible insights. To this end, we examined tractography outcomes beyond split-half reliability metrics. MSMT produced the highest fODF reproducibility overall. However, MSMT also estimated isotropic fODFs within WMH regions more often than FWE. Due to this, FWE yielded greater streamline counts and more extensive tract overlap with WMH tissue, suggesting it is more effective at preserving tract continuity through areas affected by free water. Qualitative inspection of tracts further supported this pattern. Importantly, the high reliability of these tracts suggests that these are not generated by noise in these regions, but by a reliably measured signal. When MSMT tractography failed, it tended to produce truncated or failed tracts resembling those generated by the Original preprocessing. In contrast, FWE tractography more consistently recovered tracts that traversed WMH regions. These findings suggest that while MSMT enhances fODF modeling, FWE may be better suited for robust tract delineation in the presence of free water contamination.

Our findings highlight that different free water correction strategies offer complementary benefits. MSMT provides highly reliable modeling of the diffusion signal by controlling for partial volume effects. This increased reliability propagates throughout the MSMT tractometry pipeline. FWE, while slightly less reliable than MSMT, offers advantages in reconstructing continuous tracts through regions affected by pathology. This is because FWE retains residual directional diffusion information in damaged white matter, such as WMH, where MSMT often estimates isotropic fODFs. This difference is significant in studies of aging and disease, where the ability to model or traverse damaged white matter directly affects the reliability and anatomical validity of tract-based analyses.

We demonstrated that tractometry provides strong information about individual differences in the degree of white matter hyperintensities. Regardless of the processing pipeline, a logistic regression model was able to achieve cross-validated AUC values of between 0.75 and 0.9 in distinguishing between different levels of clinician-classified Fazekas scores. We conclude that while information about the properties of the WMH is apparent in FWE tract profiles (Fig. 10), information about Fazekas is sufficiently represented in the overall properties of white matter, consistent with previous results that have linked the tissue properties of NAWM with the volume of WMH (Schmidt et al., 2010). While several other studies have demonstrated links between dMRI tissue properties and WMH (Raja et al., 2019), this, to our knowledge, is the first study that has demonstrated that tract profiles provide high accuracy in distinguishing between individuals.

The FWDTI model has come under recent renewed scrutiny, particularly in the single-shell case, where it has been demonstrated that the single-shell FWDTI model biases FWE-corrected metrics, such as FA and MD, due to non-Gaussian diffusion effects (Correia et al., 2024). We acknowledge these issues, but we emphasize that these issues are unrelated to the benefits we demonstrate here. In fact, our study avoids this pitfall because, in our approach, tractography is derived from the FWE-corrected signal, while tractometry metrics are derived from the Original, non-FWE-corrected diffusion data. By doing so, we mitigate the risk of introducing bias into the diffusion metrics and preserve the benefits of FWE tractography, ensuring a more reliable and accurate analysis of white matter tracts and tissue properties. Nevertheless, our study also demonstrates that FWE provides much larger benefits in multi-shell data. This is likely because of the ill-posed nature of the free-water model in single-shell data, which requires additional assumptions (e.g., spatial contiguity).

### 5.1 Conclusion

Our study demonstrates the benefits of applying free water modeling to improve the reliability and accuracy of tractography in aging brains, particularly in regions affected by WMH. By mitigating the effects of free water contamination, both MSMT and FWE increased the reliability of all stages of tractography: fODF estimation, tract delineation, and tract profiling. While MSMT was highly reliable overall, FWE produced greater streamline yield, more extensive overlap with WMH regions, and more anatomically complete tracts in qualitative assessments. These results suggest that while MSMT improves diffusion modeling, FWE may be better suited for robust tract delineation in the presence of free water contamination. Our findings support the use of free water modeling in tractometry pipelines to study white matter changes and its relationship to aging and disease.

## Supplementary Material

Supplementary Materials

## Data Availability

Code to perform free water elimination is available on GitHub: https://github.com/nrdg/fwe. Code to replicate all stages of the analysis is available on GitHub: https://github.com/kellychang4/fwe-manuscript. Data can be accessed through https://www.actagingresearch.org/.
