# Supplementary material for: Free water elimination tractometry for aging brains
Source: Imaging Neurosci (Camb). 2025 Nov 7;3:IMAG.a.991. doi: 10.1162/IMAG.a.991 (PMC12603662; doi:10.1162/IMAG.a.991)
Supplement: Supplementary Materials [file IMAG.a.991_supp.pdf]

Supplemental Material to “Free-water elimination tractometry for aging brains“

Kelly Chang[1*], Luke Burke[4], Nina LaPiana[2], Bradley Howlett[2],
David Hunt[2], Margaret Dezelar[4], Jalal B. Andre[3], Patti Curl[3]
James D. Ralston[4], Ariel Rokem[1,5], & Christine L. Mac Donald[2,5]

[1]Department of Psychology, University of Washington

[2]Department of Neurological Surgery, University of Washington

[3]Department of Radiology, University of Washington

[4]Kaiser Permanente Washington Health Research Institute

[5]These authors contributed equally

[*]Correspondence: kchang4@uw.edu

October 16, 2025

1 Supplemental Analyses

1.1 MSMT and FWE Compartment Fractions

The multi-shell multi-tissue (MSMT) and free-water elimination (FWE; via the free-water diffusion tensor model) methods both estimate separate diffusion compartments. MSMT decomposes the diffusion signal into three compartments: cerebrospinal fluid (CSF), gray matter (GM), and white matter (WM). In contrast, FWE models two compartments: a free water (isotropic) compartment and a white matter (anisotropic) compartment without assigning specific tissue types.

In regions affected by white matter hyperintensities (WMH), the MSMT model can overweigh the isotropic signal from WMH voxels as belonging to CSF or GM compartments due to elevated free water content in these tissues. This results in attenuated or isotropic fiber orientation distributions (fODFs)

within WMH regions. In contrast, the FWE model does not assign the free water component to specific tissue types; instead, it represents the isotropic signal as an additional compartment within the white matter voxel. This weaker assumption allows FWE to better preserve the anisotropic signal even in regions with substantial free water contamination.

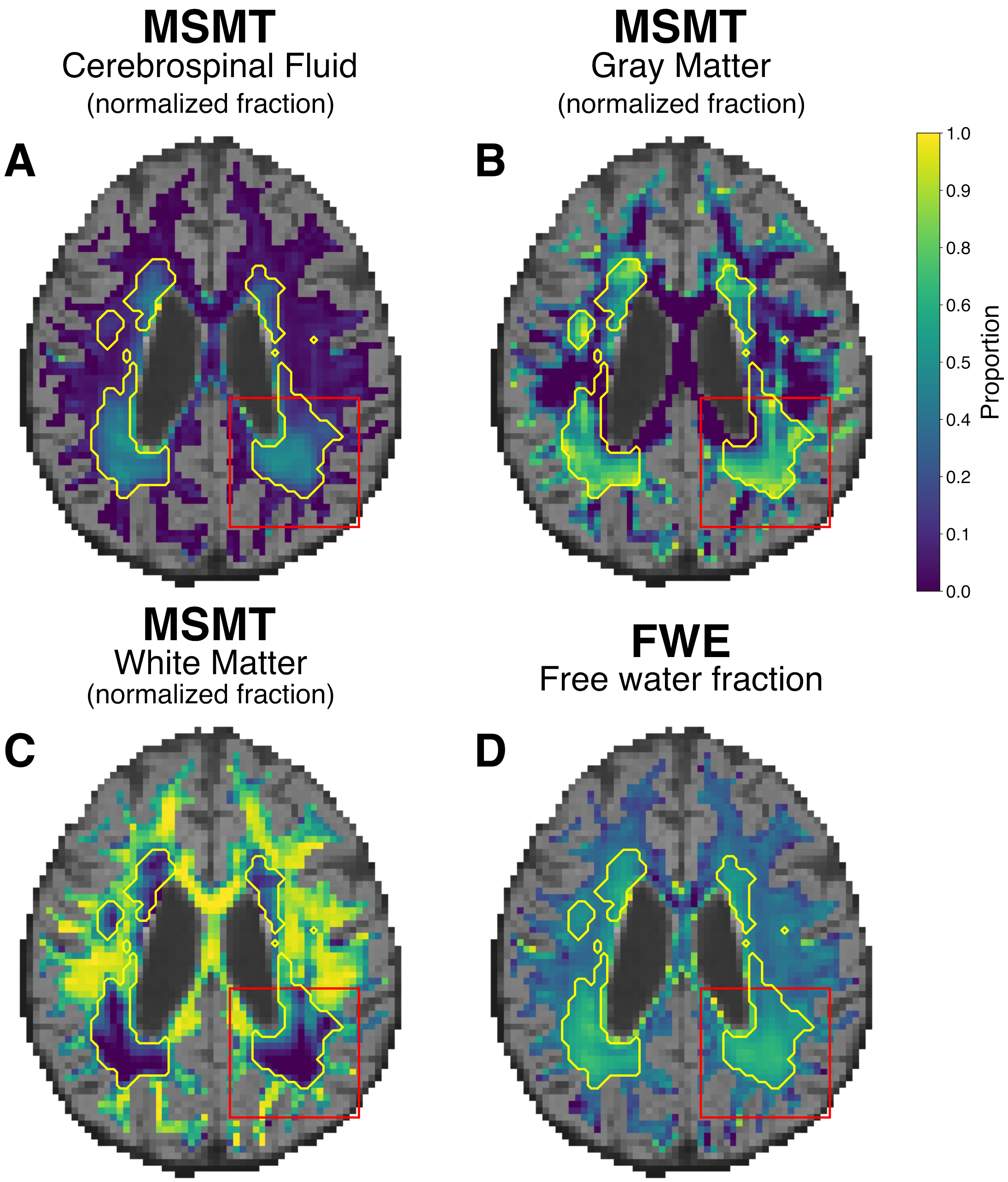

Supplemental Figure 1: Multi-shell multi-tissue (MSMT) and free water elimination (FWE) compartment fraction maps for an example participant. The participant shown here is also depicted in Figure 3 of the main text. WMH regions are outlined in yellow and the red box indicates the region enlarged in Figure 3B–D. MSMT estimates compartments for three tissue types: **(A)** cerebrospinal fluid, **(B)** gray matter, and **(C)** white matter. Whereas, FWE estimates one **(D)** free water fraction map.

1.2 Original with Lowered FA Stop Threshold

We performed the same analysis on the Original dataset using a lowered FA stop threshold for tractography (50% decrease) to determine whether the results observed with FWE and MSMT tractography could be attributed to reduced FA following free water removal. We found that lowering the FA stop threshold produced tractography results that were overall similar to the Original pipeline and did not replicate the improvements seen with FWE and MSMT tractography. These findings suggest that the benefits of FWE and MSMT are not simply due to thresholding effects but instead reflect more fundamental changes in how the diffusion signal is modeled.

1.2.1 Tract delineation reliability

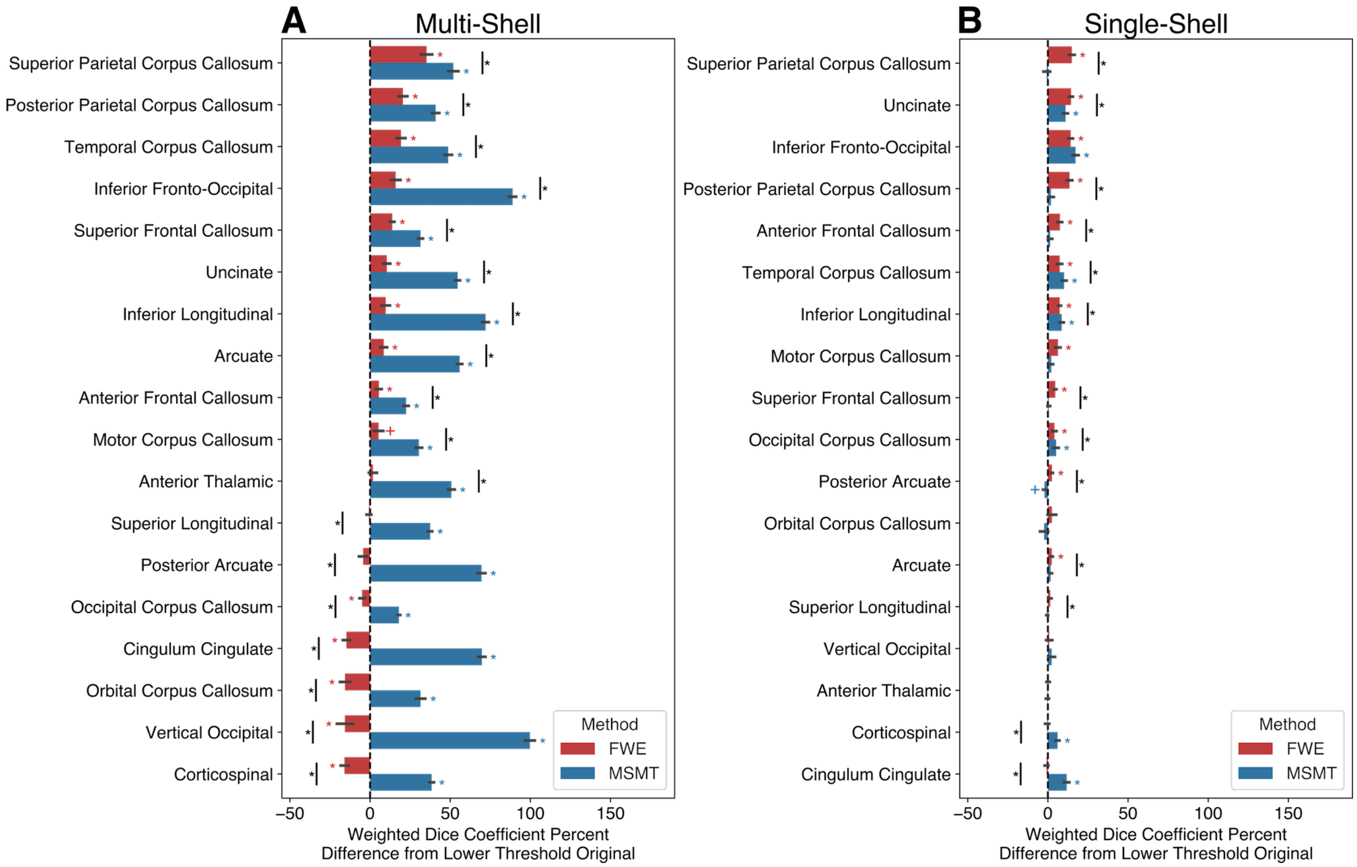

Supplemental Figure 2: Tract reliability weighted dice coefficient differences. Weighted dice coefficient differences shown for the split-half **(A)** multi-shell and **(B)** single-shell datasets collapsed across hemispheres. The difference was calculated as FWE (red) or MSMT (blue) - Original with lowered FA stop threshold. Error bars represent ± 1 SEM. Asterisks represent tracts with weighted Dice coefficient differences significantly different (Bonferonni-corrected) from 0. Crosses represent tracts with weighted Dice coefficient difference (without Bonferonni correction) from 0.

1.2.2 Tract profile reliability

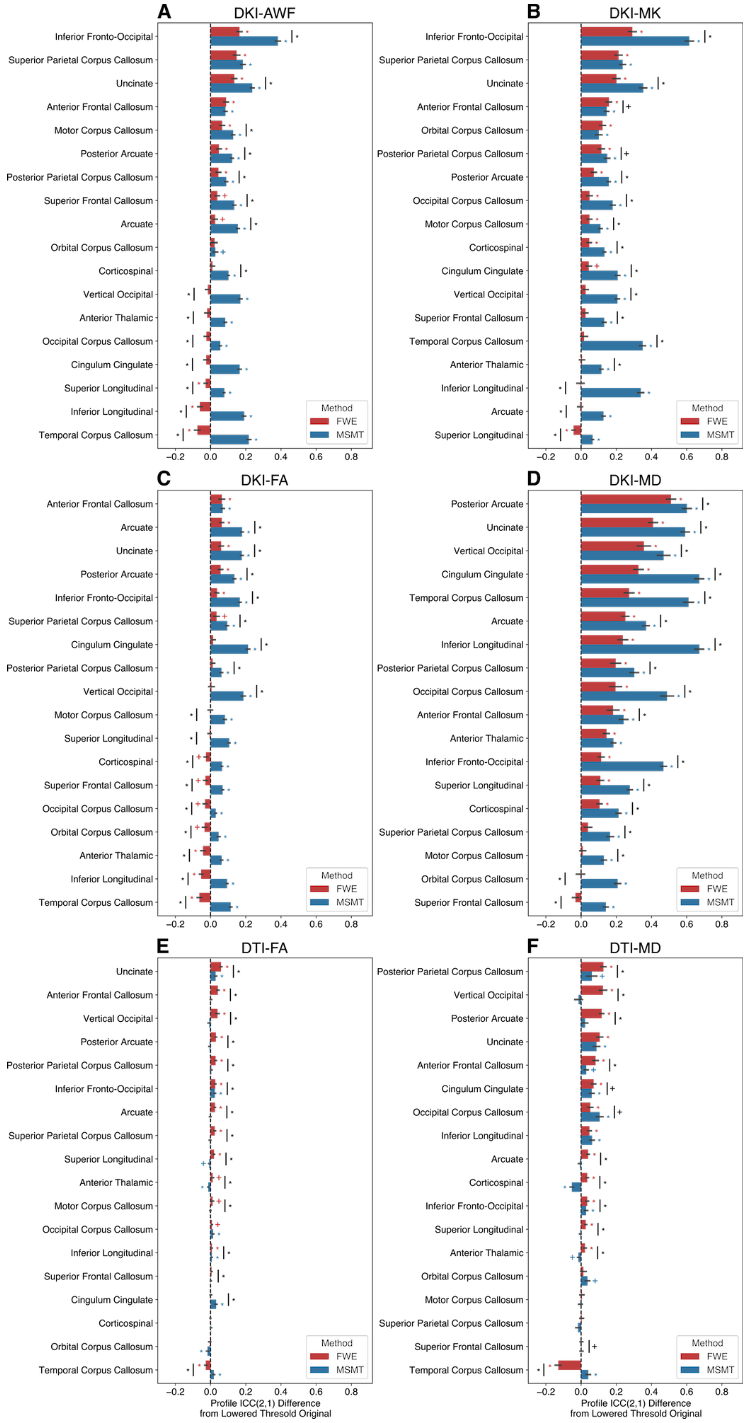

Supplemental Figure 3: Differences in tract profile ICC(2,1). Tract profile ICC(2,1) differences are shown for split-half multi-shell **(A)** DKI-AWF, **(B)** DKI-MD, **(C)** DKI-FA, and **(D)** DKI-MD. Tract profile ICC(2,1) differences are shown split-half single-shell **(E)** DTI-FA and **(F)** DTI-MD. The difference was calculated as FWE (red) or MSMT (blue) - Original with lowered FA stop threshold. Error bars represent ± 1 SEM.

1.2.3 Tract yield

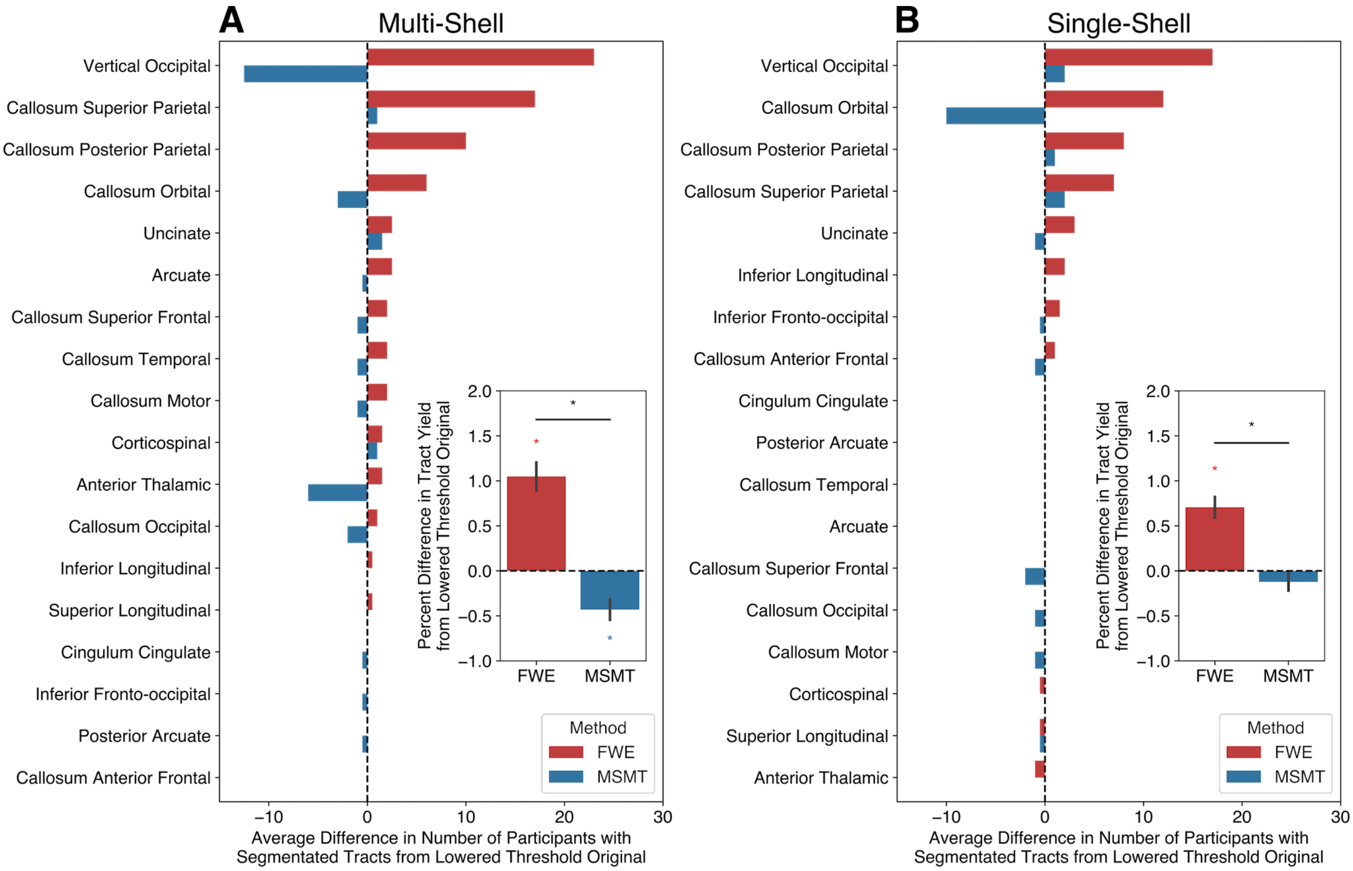

Supplemental Figure 4: Tract yield differences. Tract yield difference in the number of participants with successfully segmented tracts between FWE (red) or MSMT (blue) from the Original with lowered FA stop threshold processing for **(A)** multi-shell and **(B)** single-shell datasets. Insets show overall percent differences.

1.2.4 WMH overlap

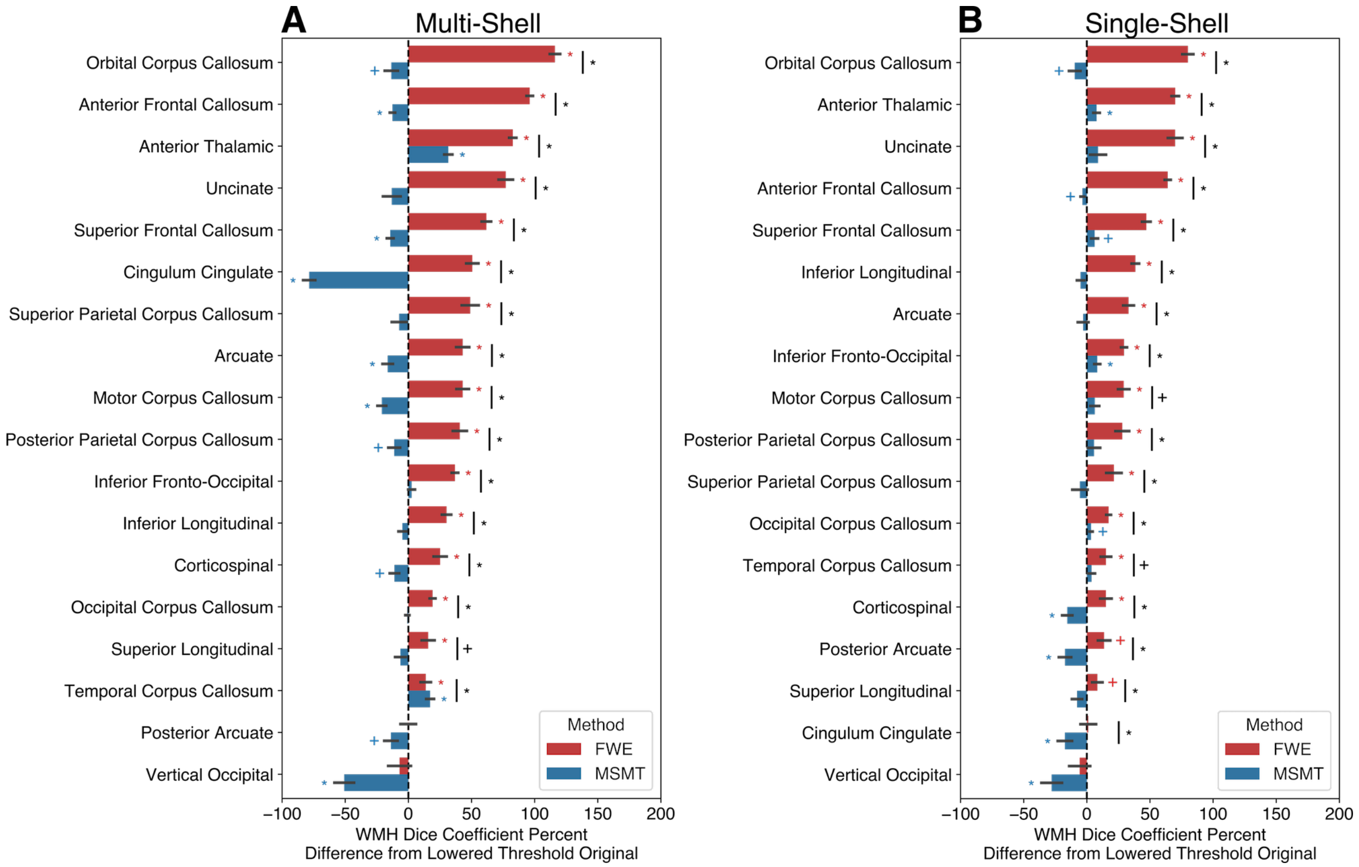

Supplemental Figure 5: Tract and white matter hyperintensity weighted dice coefficient differences. Weighted dice coefficient differences shown for the **(A)** multi-shell and **(B)** single-shell datasets collapsed across hemispheres. The difference was calculated as FWE (red) or MSMT (blue) - Original with lowered FA stop threshold. Error bars represent ± 1 SEM. Asterisks represent tracts with weighted Dice coefficient differences significantly different (Bonferonni-corrected) from 0. Crosses represent tracts with weighted Dice coefficient difference (without Bonferonni correction) from 0.

1.2.5 Fazekas score predictions

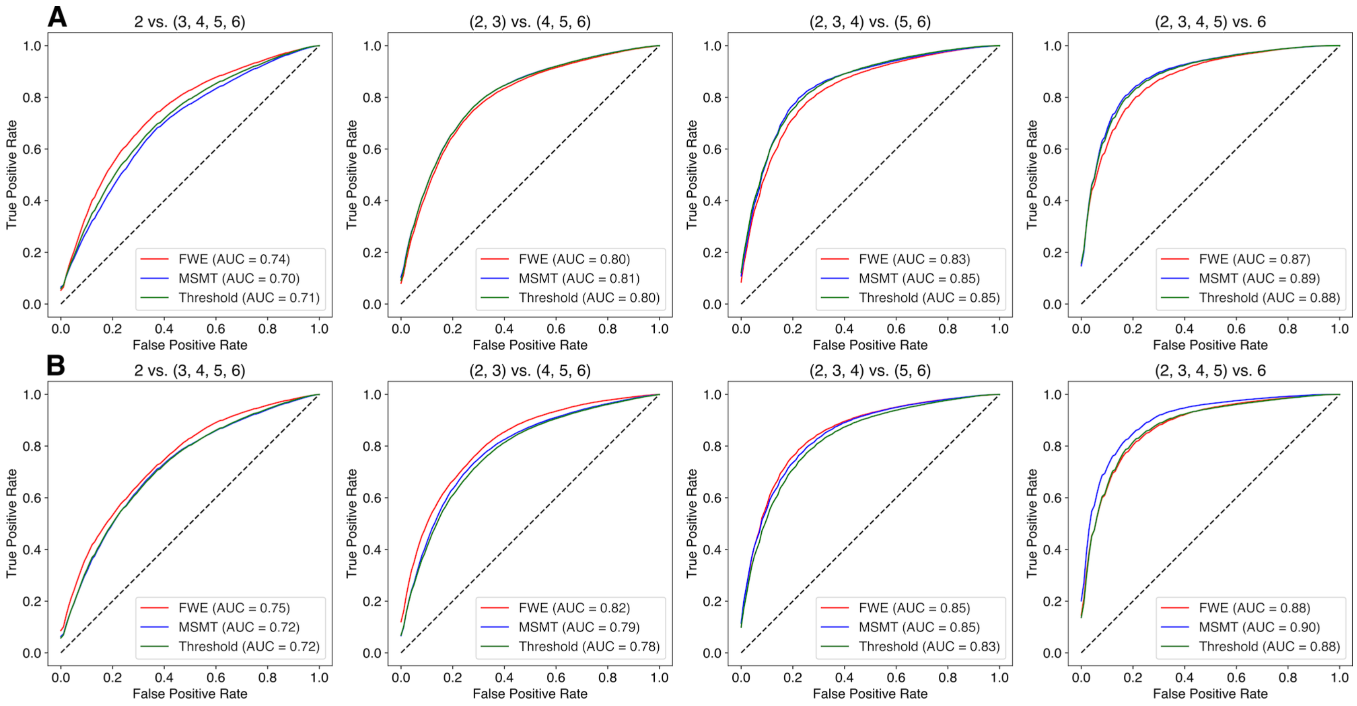

Supplemental Figure 6: Fazekas score receiver operating characteristic (ROC) curves and area under the curve (AUC) values. **(A)** The first row shows the ROC curves and AUC for the multi-shell data. **(B)** The second row shows the ROC curves and AUC for the single-shell data. The line colors correspond to each method, FWE (red), MSMT (green), and Original with lowered FA stop threshold (blue).

1.3 Shared White Matter Stop Mask

As a second control analysis, we modified the tractography pipeline to use a shared white matter (WM) stop mask, derived from each participant's QSIPrep output, across all three methods (Original, FWE, and MSMT). This was done to standardize streamline termination across methods and isolate the effects of differences in the stop mask, which may otherwise vary due to method-specific changes in FA (e.g., lower FA following free water removal). We found that the reliability differences between methods remained under this analysis, with FWE and MSMT still demonstrating greater reliability, although slightly weaker than in the main analysis. These findings suggest that the observed improvements with FWE and MSMT are not solely due to differences in tissue mask definitions, but instead reflect differences in the underlying diffusion modeling.

1.3.1 Tract delineation reliability

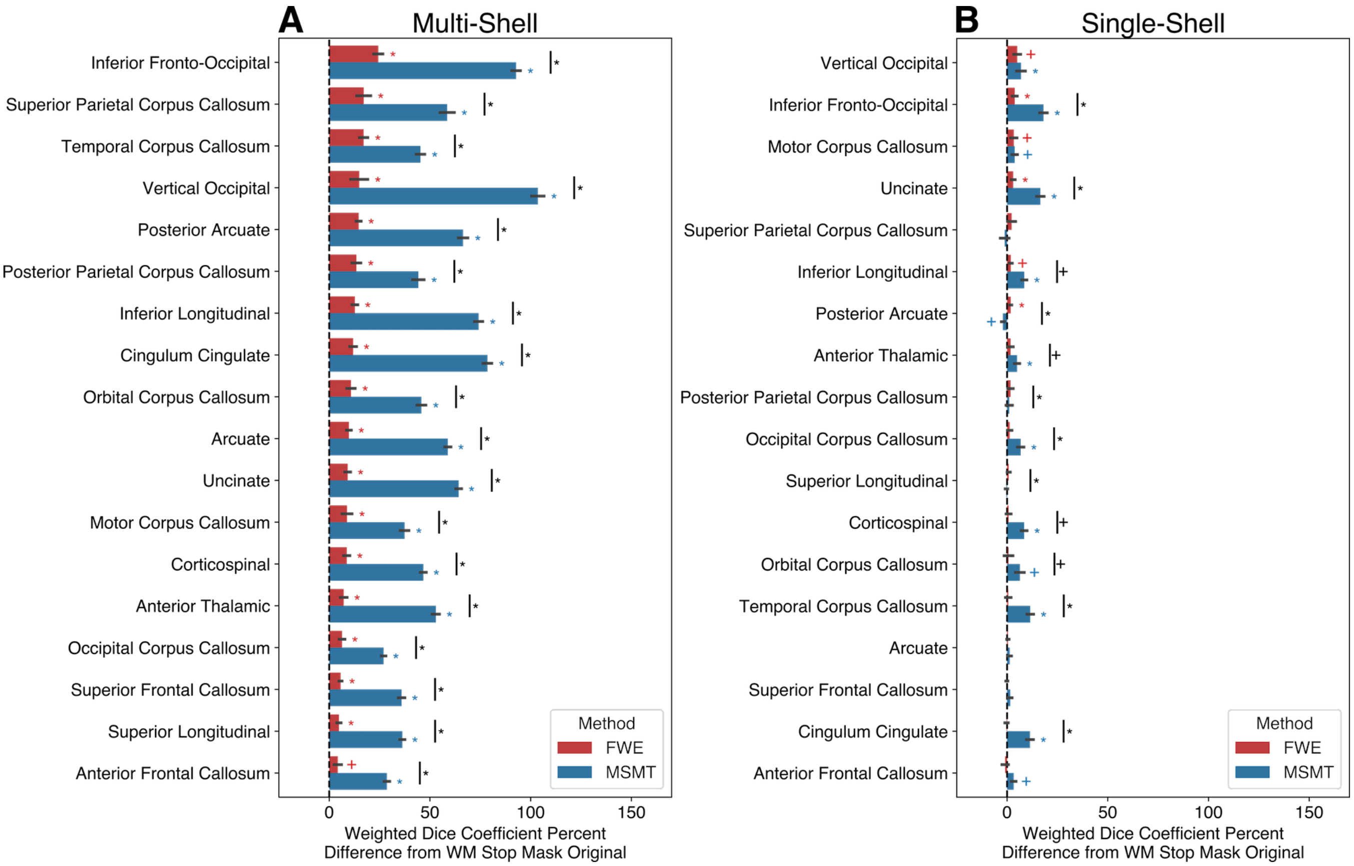

Supplemental Figure 7: Tract reliability weighted dice coefficient differences. Weighted dice coefficient differences shown for the split-half **(A)** multi-shell and **(B)** single-shell datasets collapsed across hemispheres. The difference was calculated as FWE (red) or MSMT (blue) - Original with shared white matter stop masks. Error bars represent ± 1 SEM. Asterisks represent tracts with weighted Dice coefficient differences significantly different (Bonferonni-corrected) from 0. Crosses represent tracts with weighted Dice coefficient difference (without Bonferonni correction) from 0.

1.3.2 Tract profile reliability

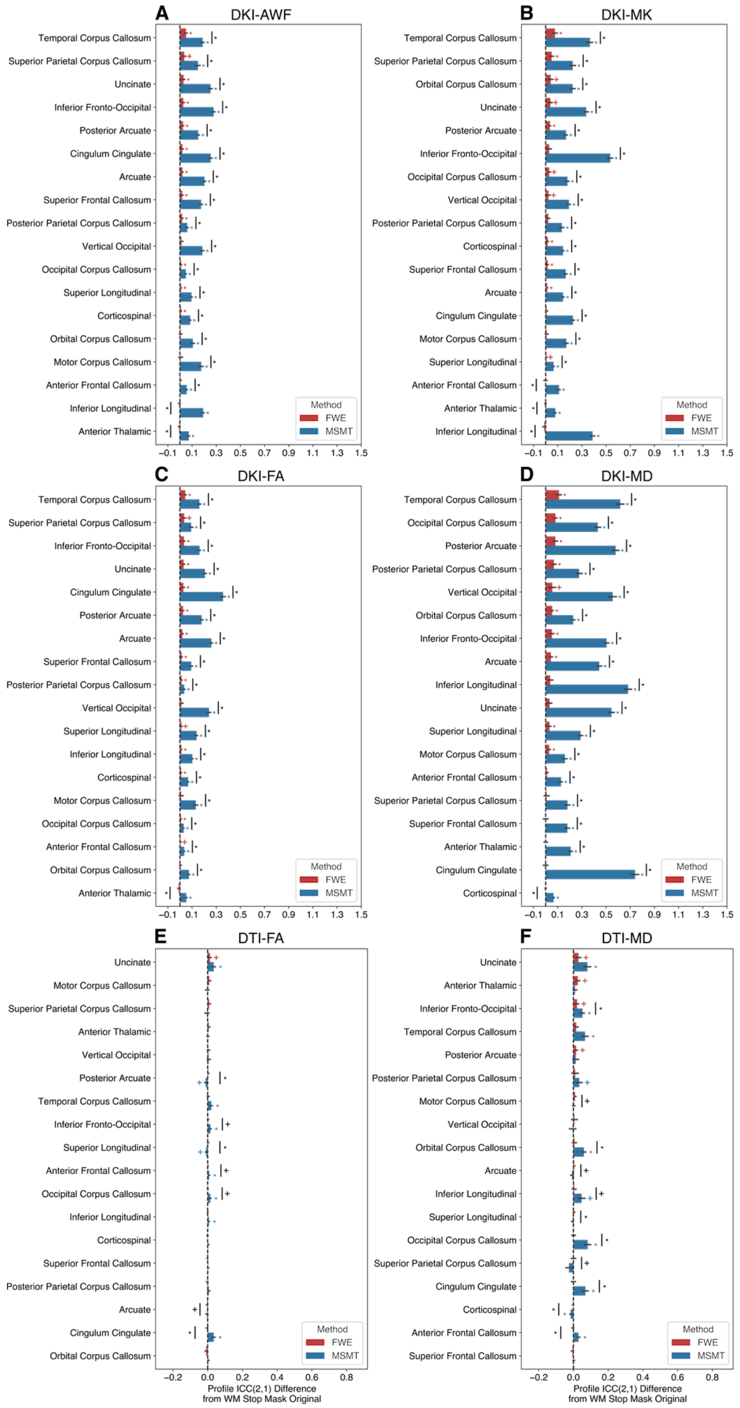

Supplemental Figure 8: Differences in tract profile ICC(2,1). Tract profile ICC(2,1) differences are shown for split-half multi-shell **(A)** DKI-AWF, **(B)** DKI-MD, **(C)** DKI-FA, and **(D)** DKI-MD. Tract profile ICC(2,1) differences are shown split-half single-shell **(E)** DTI-FA and **(F)** DTI-MD. The difference was calculated as FWE (red) or MSMT (blue) - Original with shared white matter stop masks. Error bars represent ± 1 SEM.

1.3.3 Tract yield

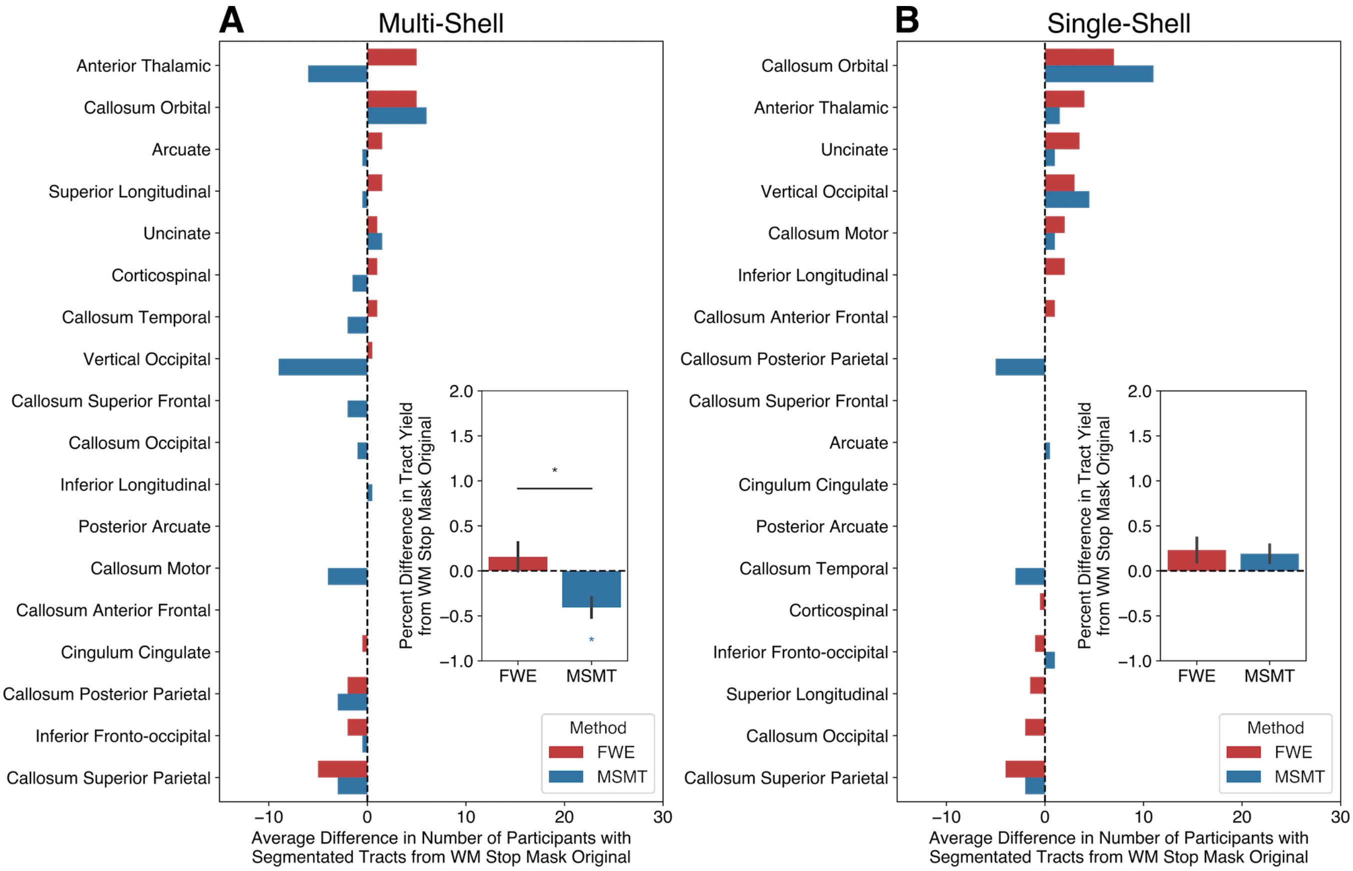

Supplemental Figure 9: Tract yield differences. Tract yield difference in the number of participants with successfully segmented tracts between FWE (red) or MSMT (blue) from the Original with shared white matter stop masks processing for **(A)** multi-shell and **(B)** single-shell datasets. Insets show overall percent differences.

1.3.4 WMH overlap

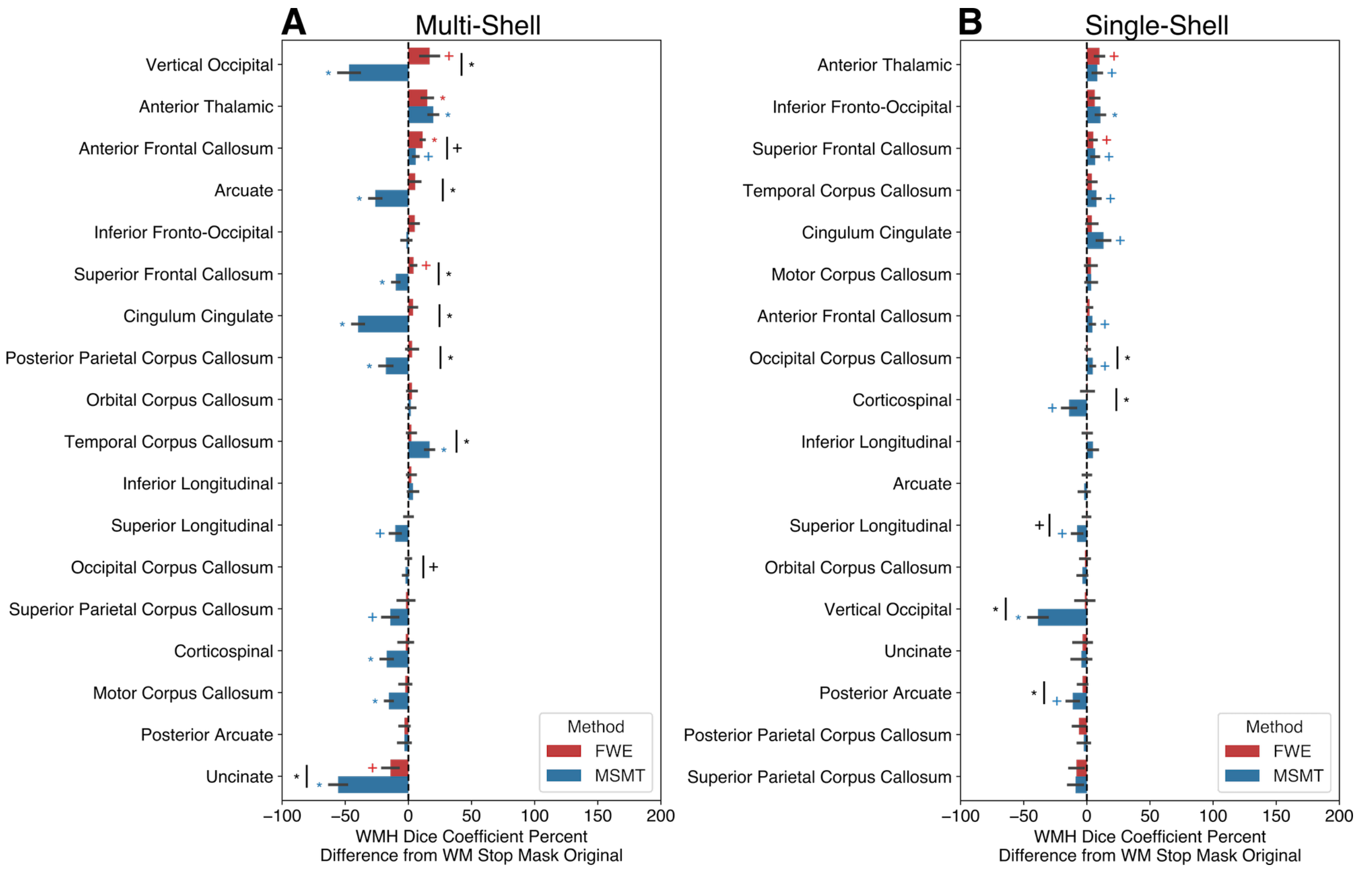

Supplemental Figure 10: Tract and white matter hyperintensity weighted dice coefficient differences. Weighted dice coefficient differences shown for the **(A)** multi-shell and **(B)** single-shell datasets collapsed across hemispheres. The difference was calculated as FWE (red) or MSMT (blue) - Original with shared white matter stop masks. Error bars represent ± 1 SEM. Asterisks represent tracts with weighted Dice coefficient differences significantly different (Bonferonni-corrected) from 0. Crosses represent tracts with weighted Dice coefficient difference (without Bonferonni correction) from 0.

1.3.5 Fazekas score predictions

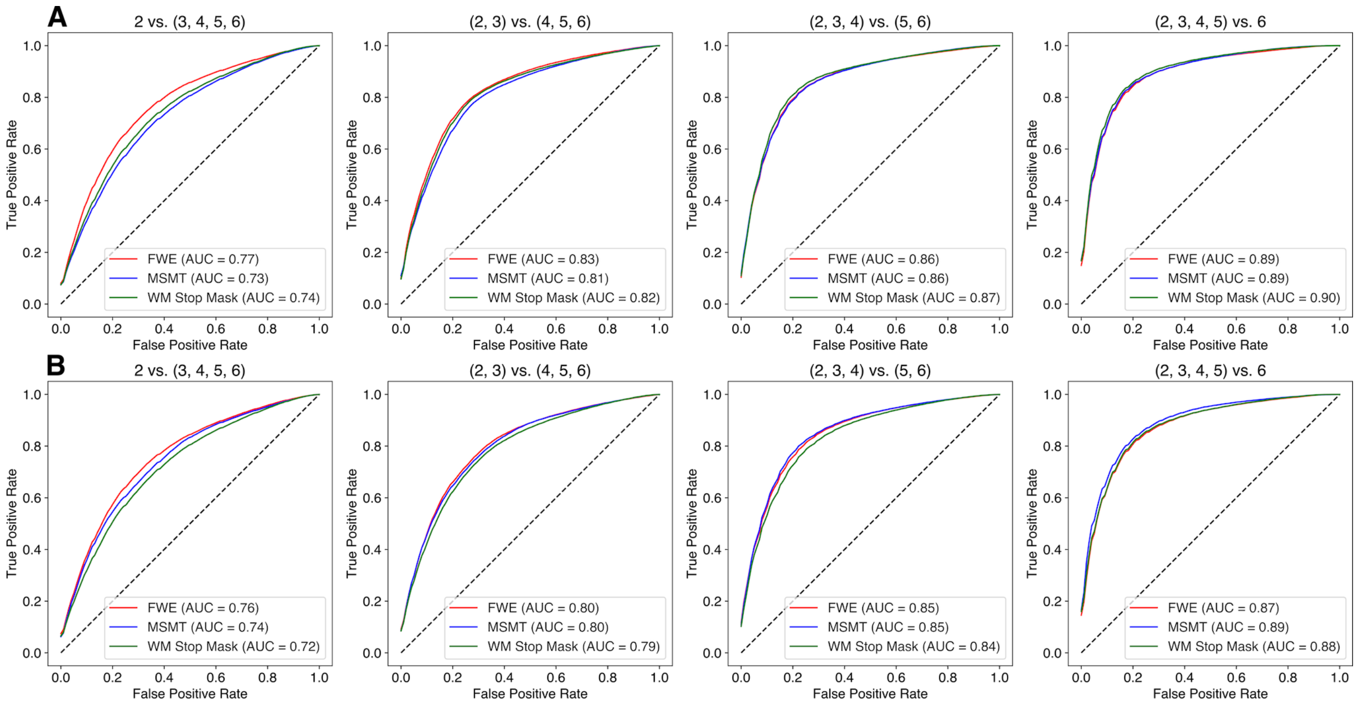

Supplemental Figure 11: Fazekas score receiver operating characteristic (ROC) curves and area under the curve (AUC) values. **(A)** The first row shows the ROC curves and AUC for the multi-shell data. **(B)** The second row shows the ROC curves and AUC for the single-shell data. The line colors correspond to each method, FWE (red), MSMT (green), and Original (blue) with shared white matter stop mask.

1.4 HCP Test-Retest dataset

We performed the same analysis using the HCP Test-Retest dataset to evaluate whether the differences observed with FWE and MSMT tractography were specific to aging populations. In this younger, healthy cohort, we found that the impact of FWE and MSMT on tractography was minimal, suggesting that the advantages of free water elimination and multi-shell modeling are more pronounced in datasets with age-related white matter changes.

1.4.1 fODF reliability

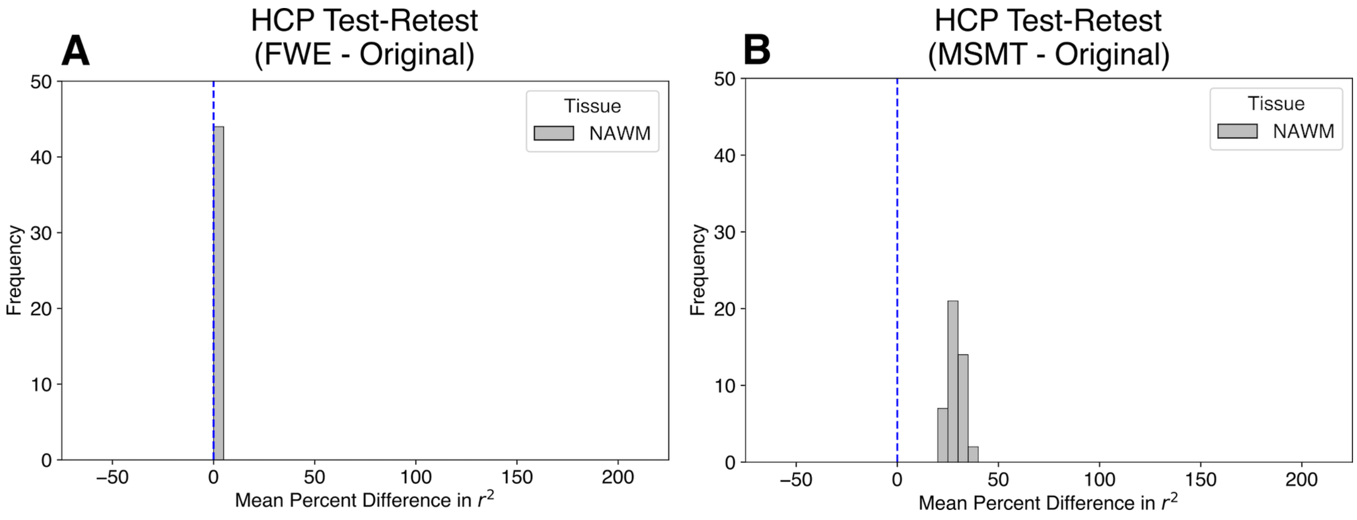

Supplemental Figure 12: Fiber orientation distribution functions (fODFs) intersession reliability. Histogram of **(A)** FWE and **(B)** MSMT HCP Test-Retest intersession fODF reliability percent difference in normal appearing white matter (NAWM).

1.4.2 Tract delineation reliability

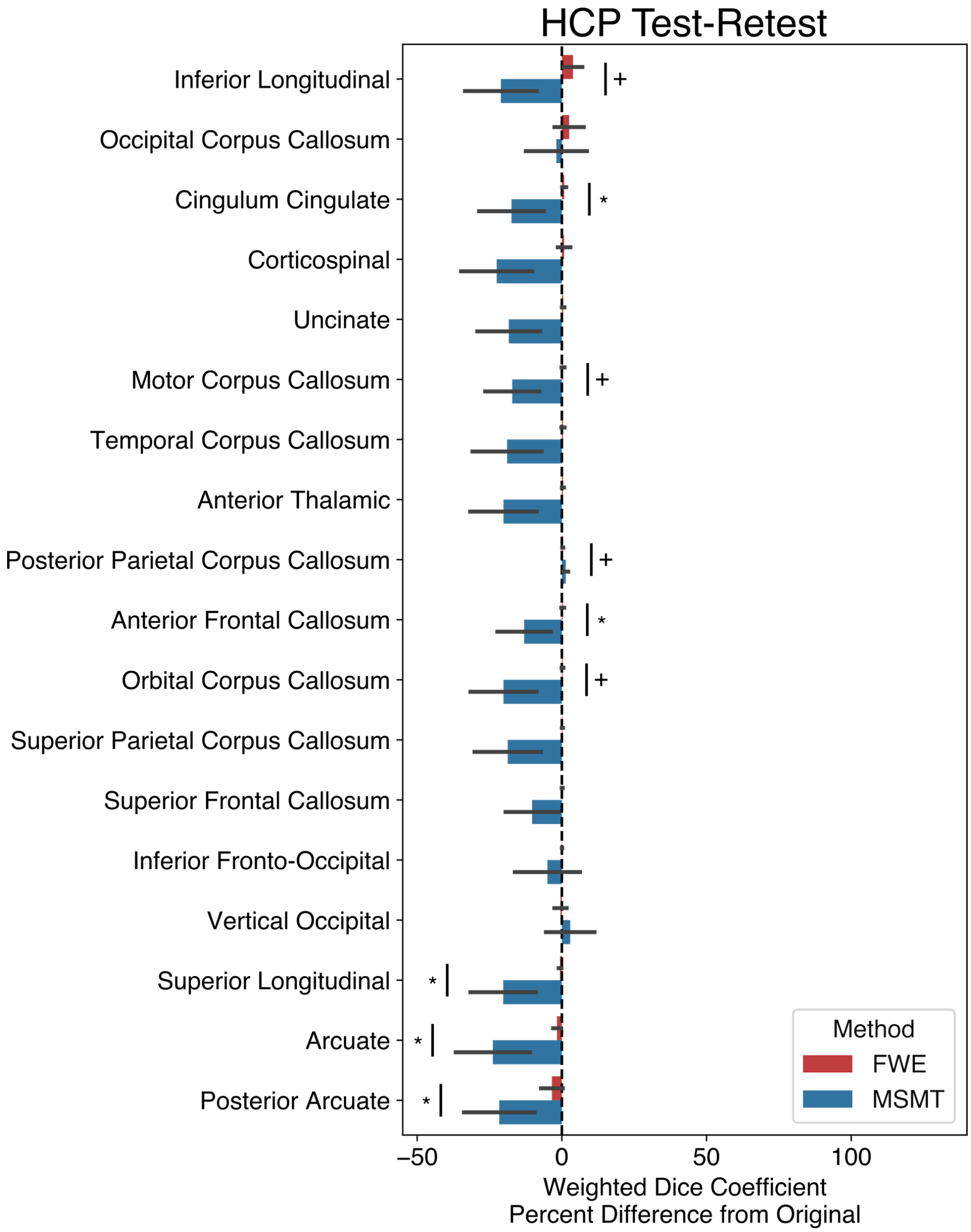

Supplemental Figure 13: Tract reliability weighted dice coefficient differences. Weighted dice coefficient differences shown for the intersession HCP Test-Retest dataset collapsed across hemispheres. The difference was calculated as FWE (red) or MSMT (blue) - Original. Error bars represent ± 1 SEM. Asterisks represent tracts with weighted Dice coefficient differences significantly different (Bonferonni-corrected) from 0. Crosses represent tracts with weighted Dice coefficient difference (without Bonferonni correction) from 0.

1.4.3 Tract profile reliability

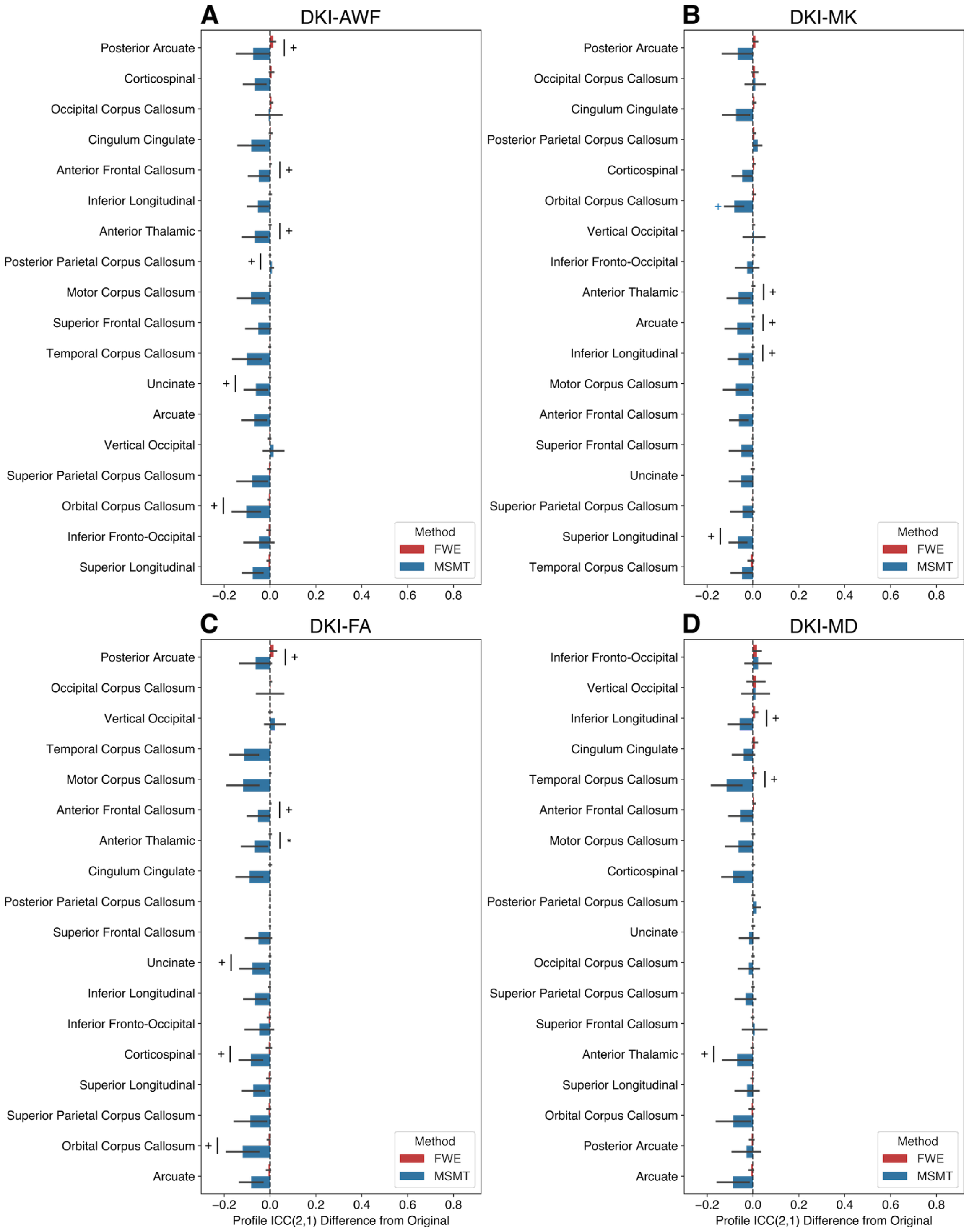

Supplemental Figure 14: Differences in tract profile ICC(2,1). Tract profile ICC(2,1) differences are shown for intersession HCP Test-Retest **(A)** DKI-AWF, **(B)** DKI-MD, **(C)** DKI-FA, and **(D)** DKI-MD. The difference was calculated as FWE (red) or MSMT (blue) - Original. Error bars represent ± 1 SEM.

1.4.4 Tract yield

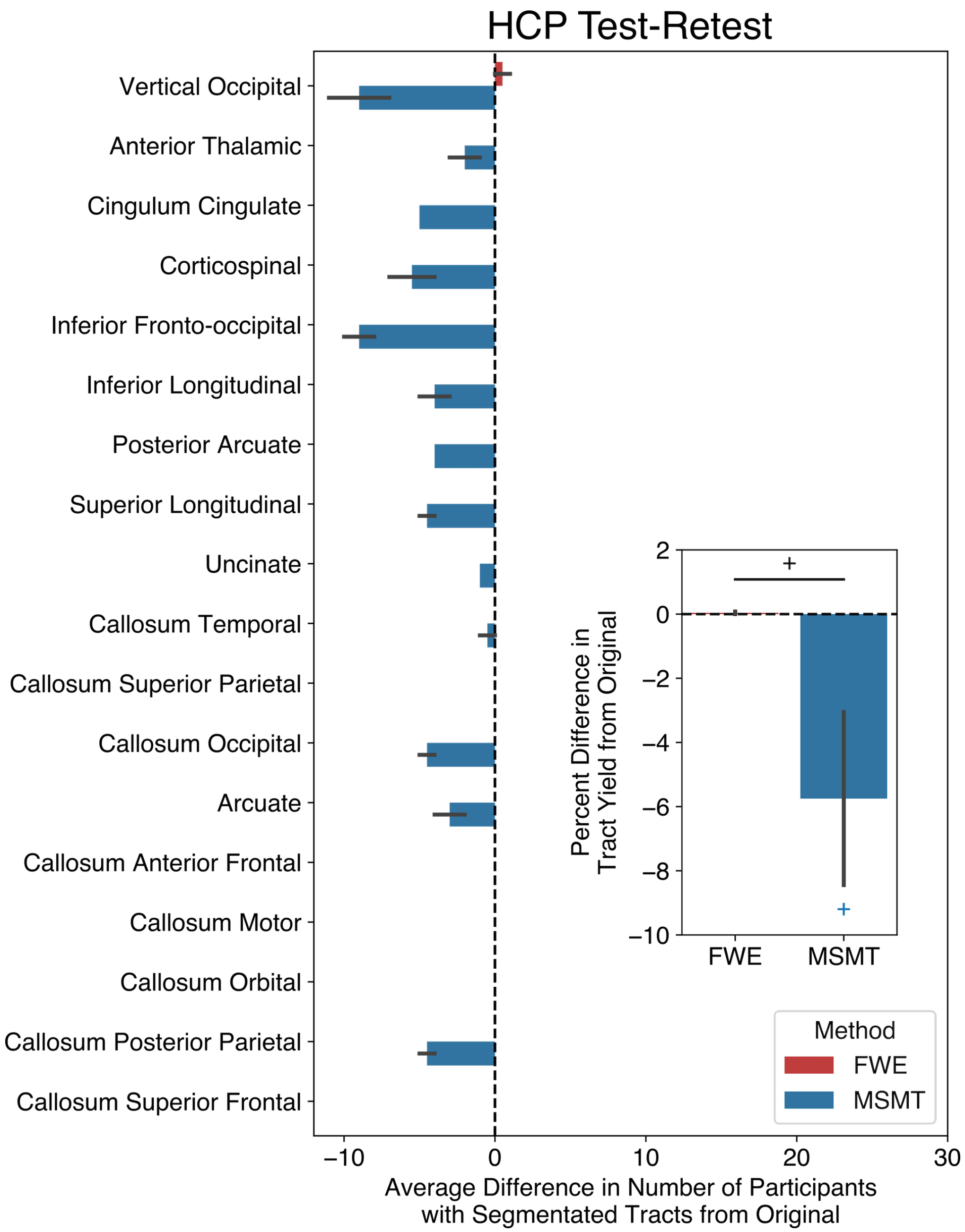

Supplemental Figure 15: Tract yield differences. Tract yield difference in the number of participants with successfully segmented tracts between FWE (red) or MSMT (blue) from the Original processing for the HCP Test-Retest dataset. Insets show overall percent differences.

1.5 Tract Examples

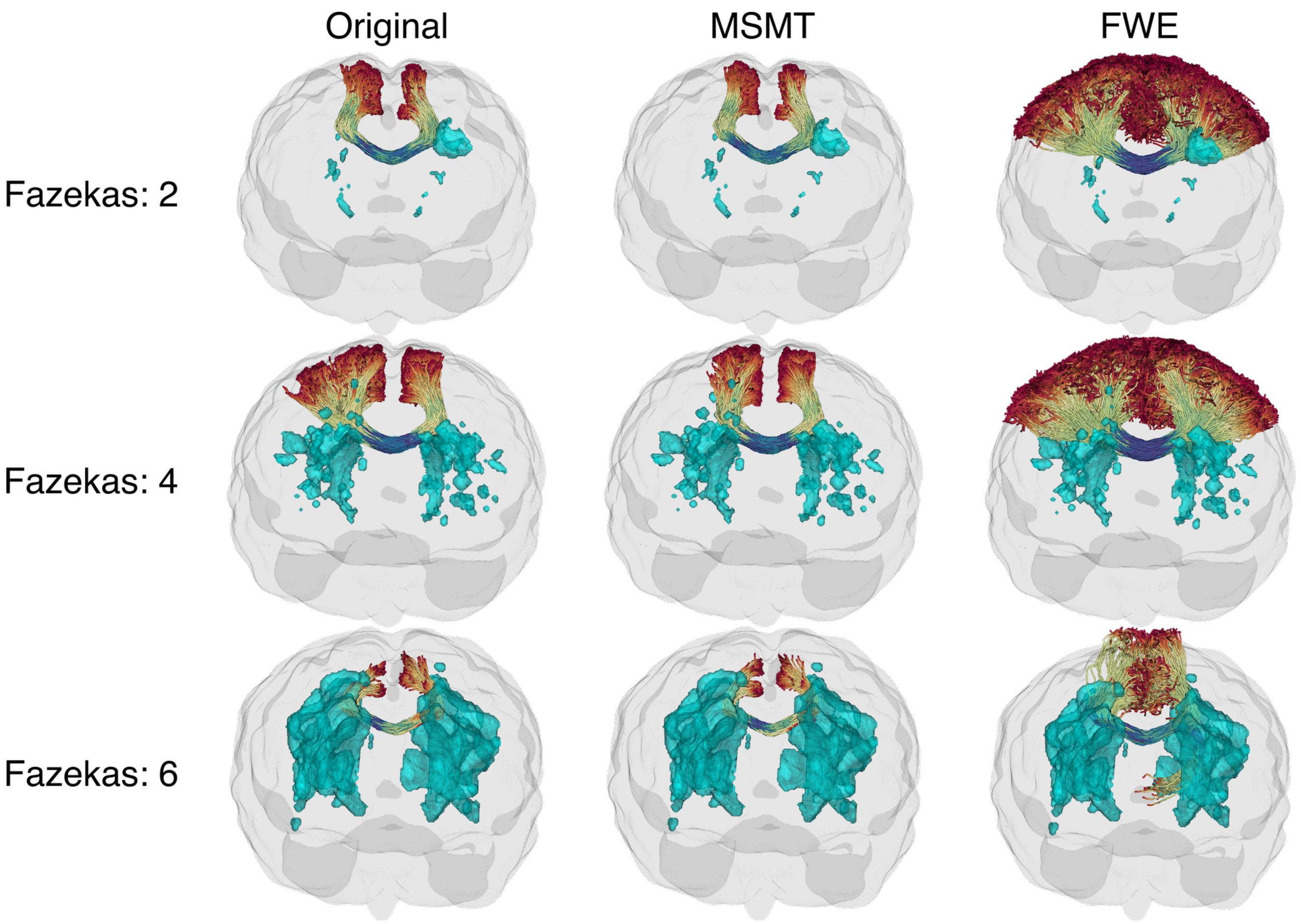

Supplemental Figure 16: Example corpus callosum motor region section by Fazekas score and processing methods. Cyan regions represent WMH areas. Fazekas scores increase by row, and processing methods differ by column.

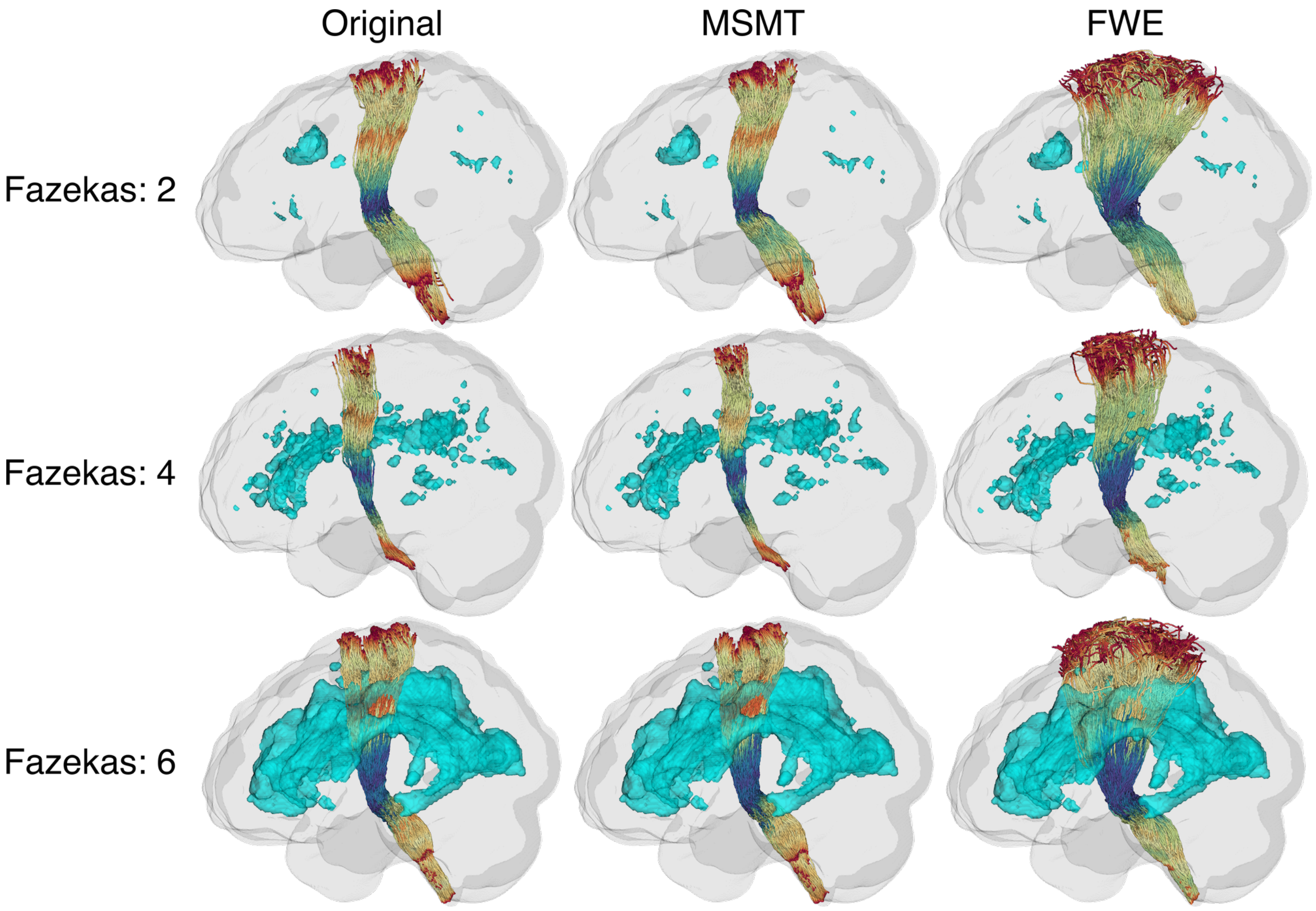

Supplemental Figure 17: Example left corticospinal tract by Fazekas score and processing methods. Cyan regions represent WMH areas. Fazekas scores increase by row, and processing methods differ by column.

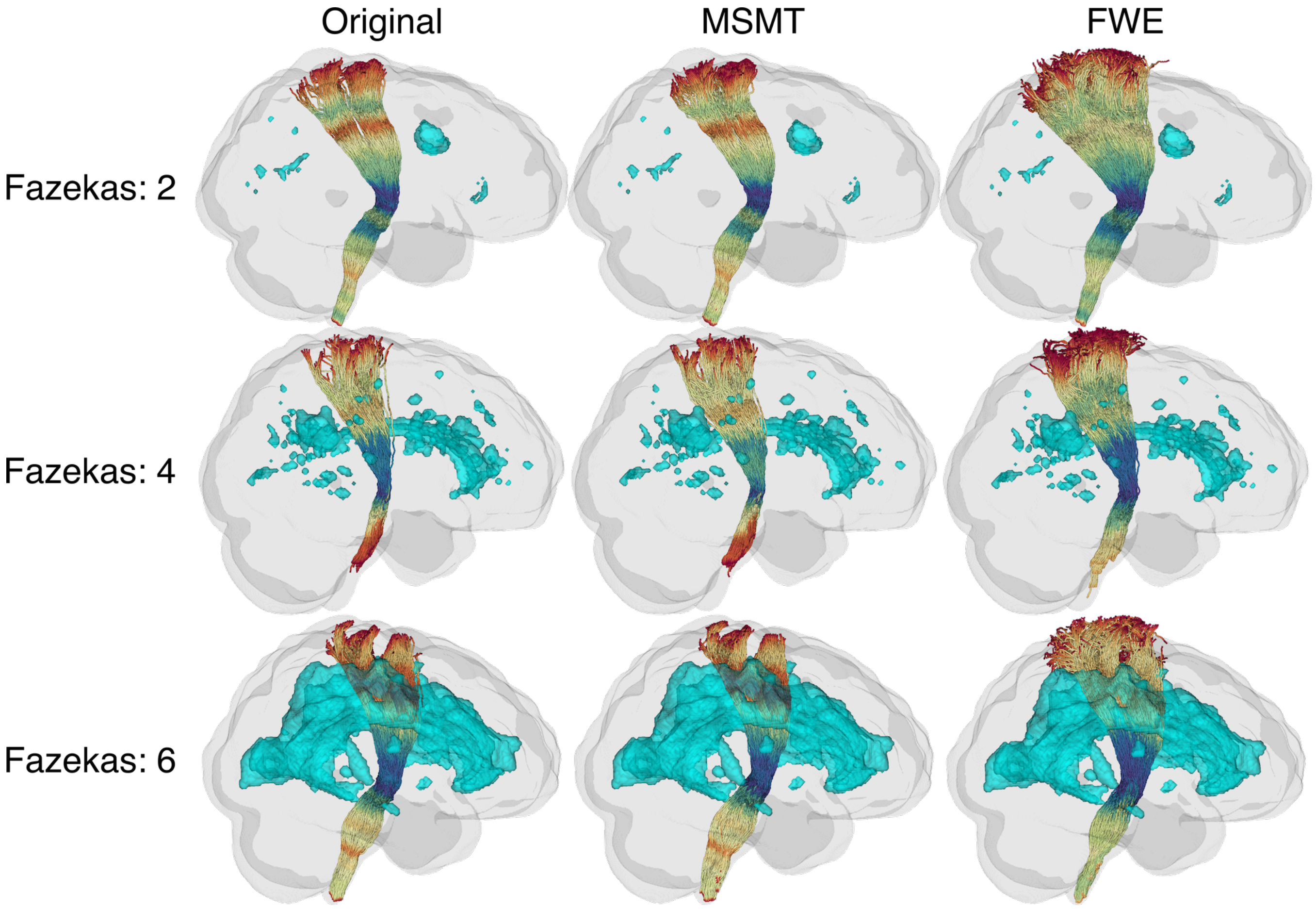

Supplemental Figure 18: Example right corticospinal tract by Fazekas score and processing methods. Cyan regions represent WMH areas. Fazekas scores increase by row, and processing methods differ by column.

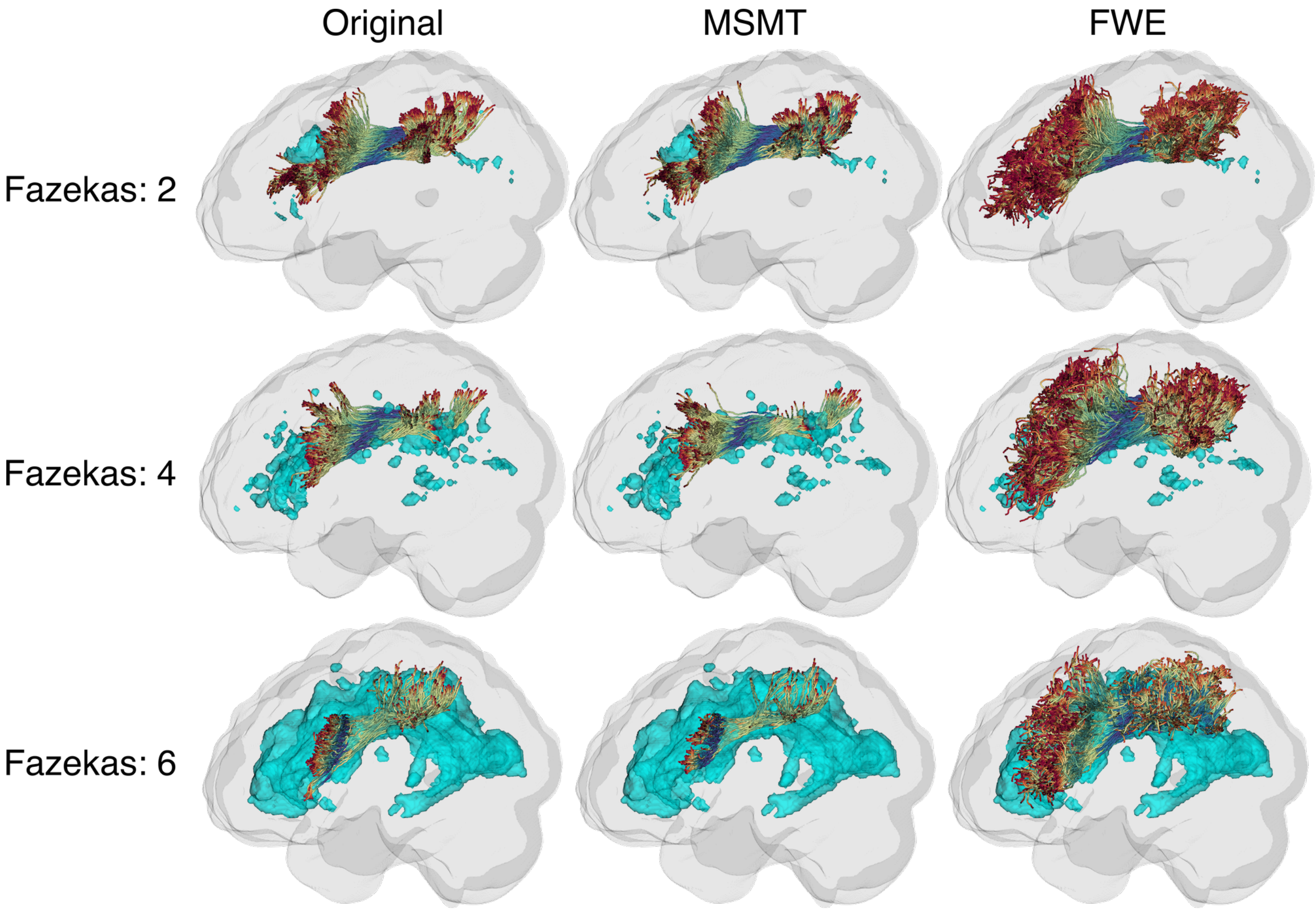

Supplemental Figure 19: Example left superior longitudinal fasciculus by Fazekas score and processing methods. Cyan regions represent WMH areas. Fazekas scores increase by row, and processing methods differ by column.

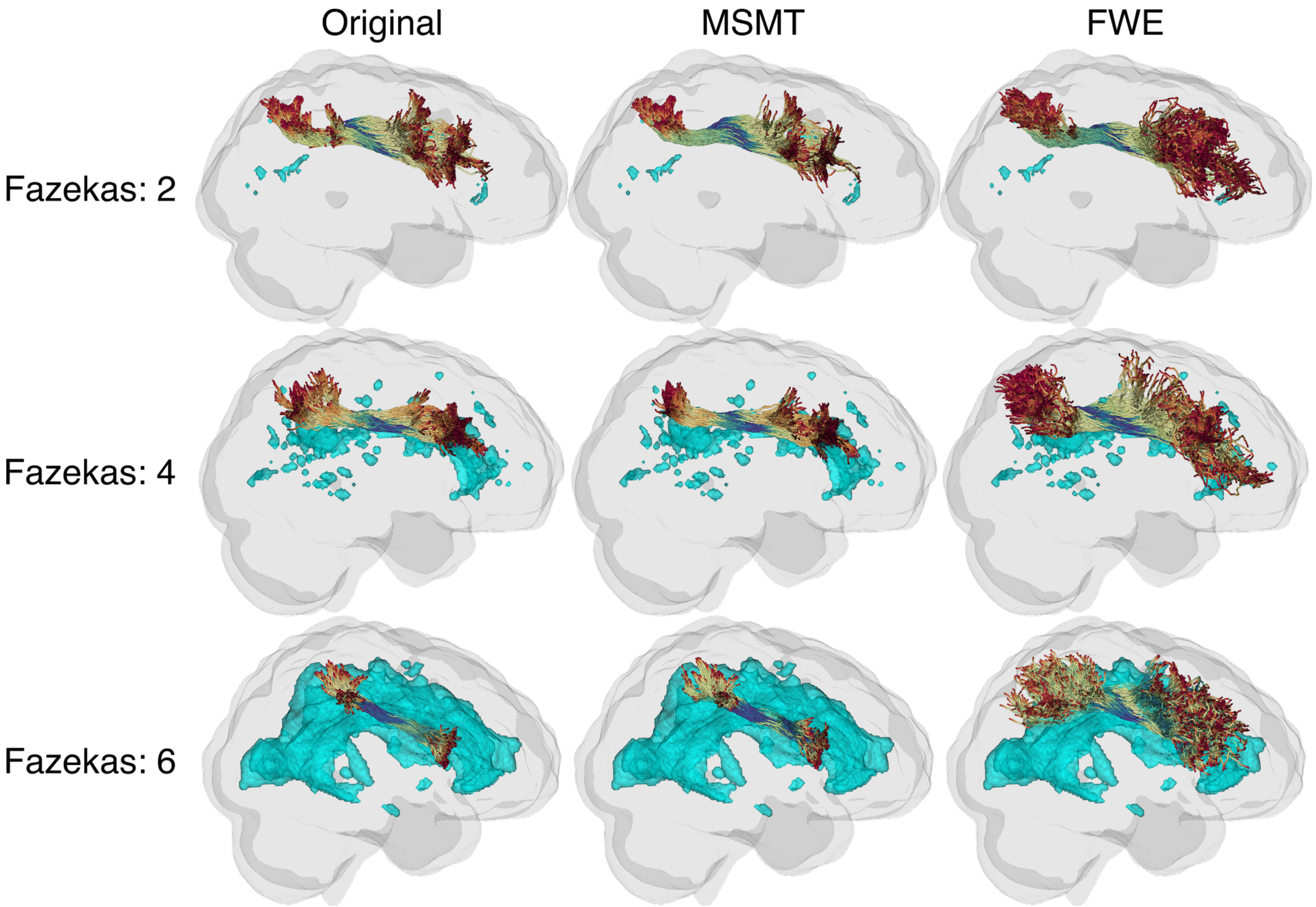

Supplemental Figure 20: Example right superior longitudinal fasciculus by Fazekas score and processing methods. Cyan regions represent WMH areas. Fazekas scores increase by row, and processing methods differ by column.

1.6 Tract Profiles

We provide the tract profiles for each tract, method, and metric from the multi-shell and single-shell datasets.

1.6.1 Multi-shell tract profiles

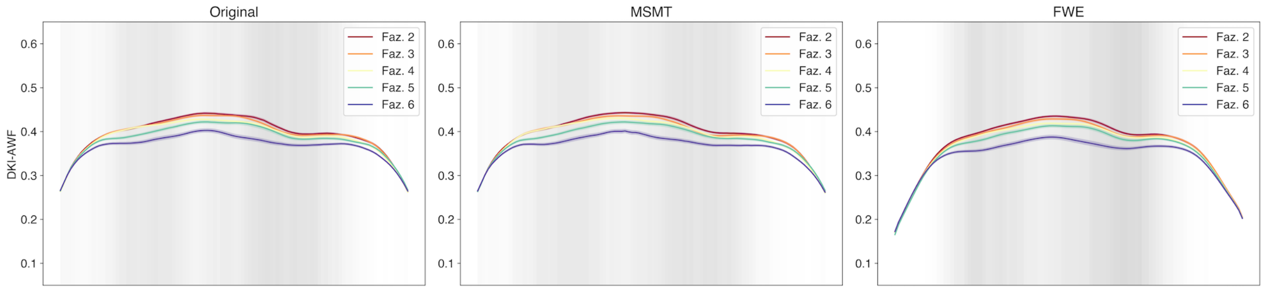

Supplemental Figure 21: Multi-shell Left Arcuate Fasiculus DKI-AWF profiles.

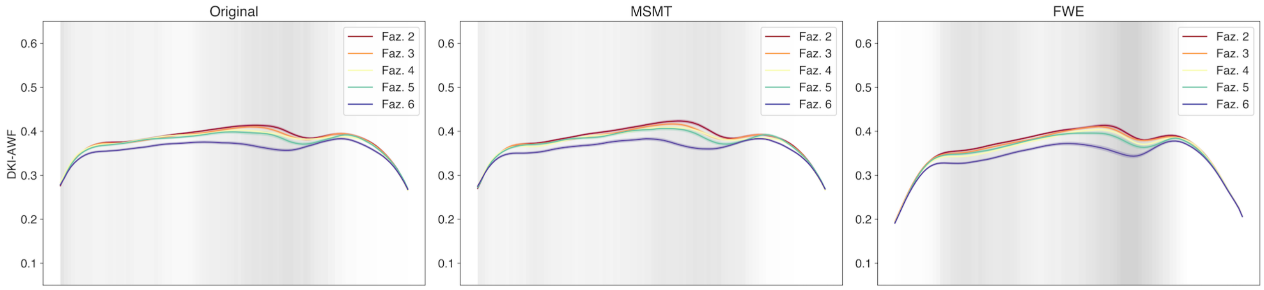

Supplemental Figure 22: Multi-shell Right Arcuate Fasiculus DKI-AWF profiles.

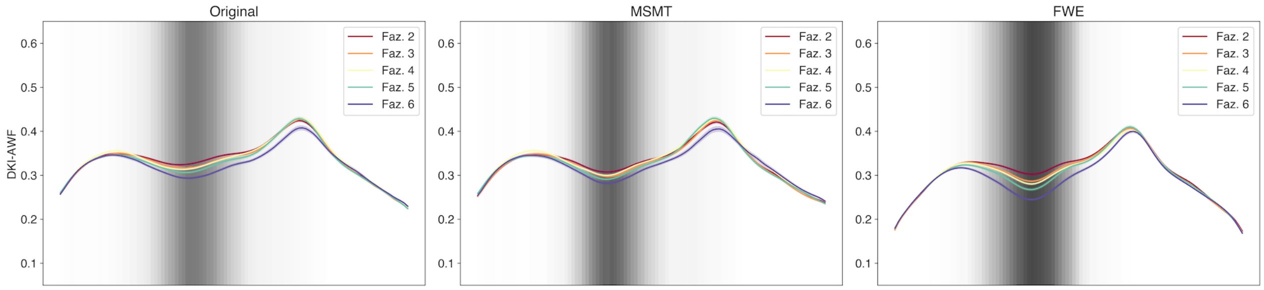

Supplemental Figure 23: Multi-shell Left Anterior Thalamic Radiation DKI-AWF profiles.

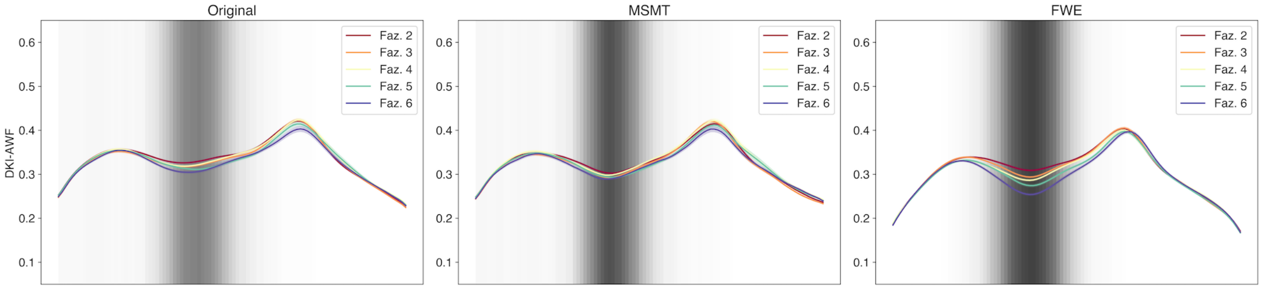

Supplemental Figure 24: Multi-shell Right Anterior Thalamic Radiation DKI-AWF profiles.

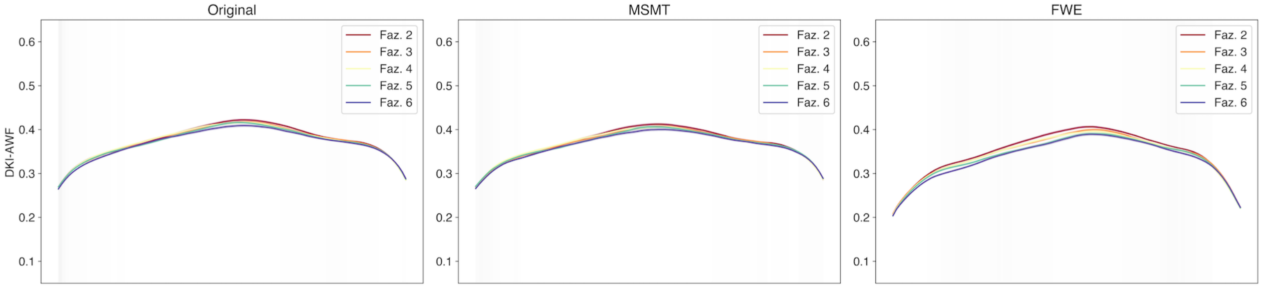

Supplemental Figure 25: Multi-shell Left Cingulum Cingulate DKI-AWF profiles.

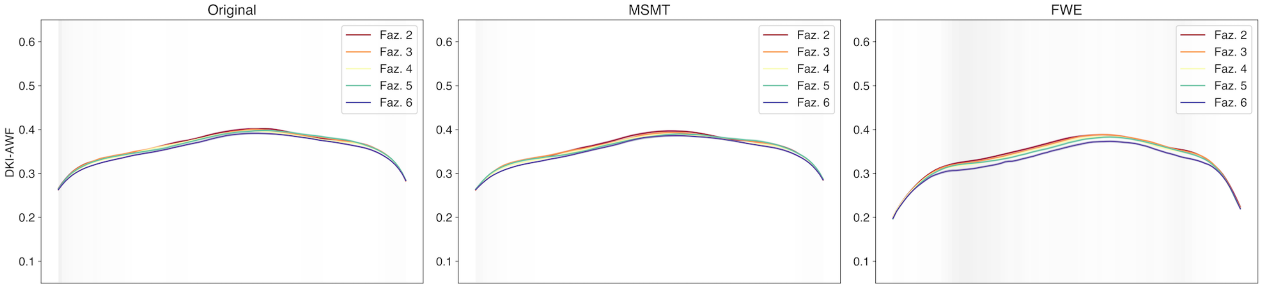

Supplemental Figure 26: Multi-shell Right Cingulum Cingulate DKI-AWF profiles.

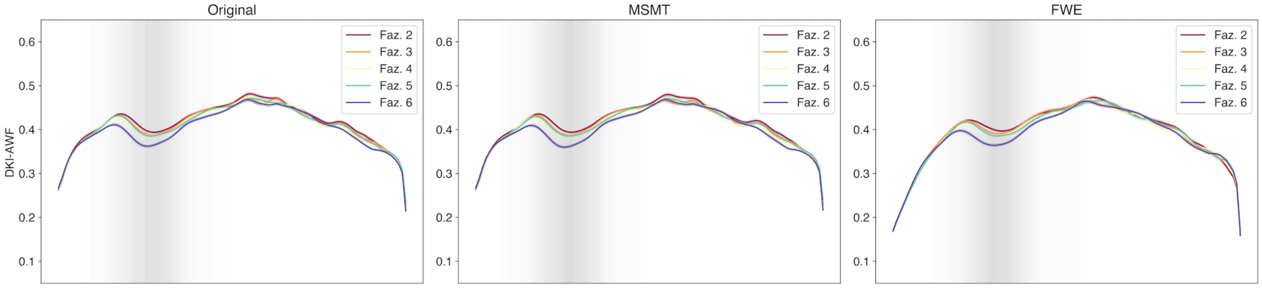

Supplemental Figure 27: Multi-shell Left Corticospinal Tract DKI-AWF profiles.

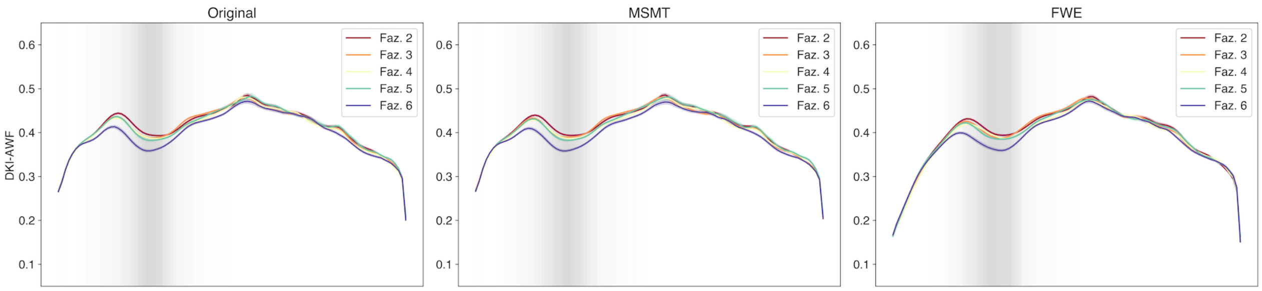

Supplemental Figure 28: Multi-shell Right Corticospinal Tract DKI-AWF profiles.

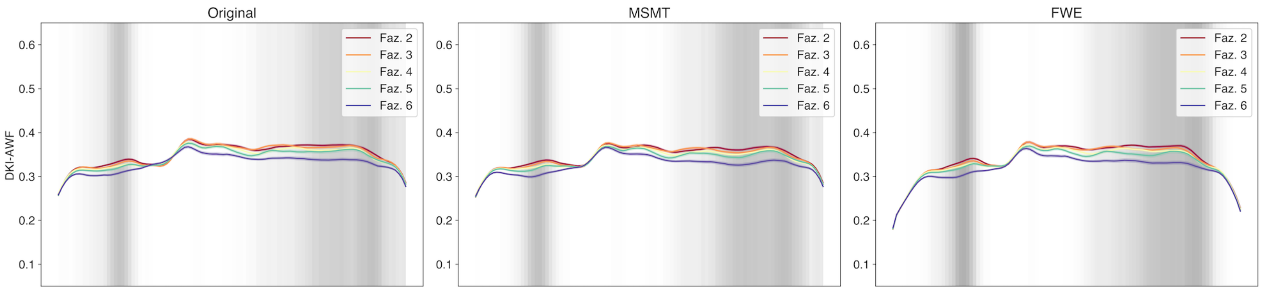

Supplemental Figure 29: Multi-shell Left Inferior Fronto-Occipital Fasiculus DKI-AWF profiles.

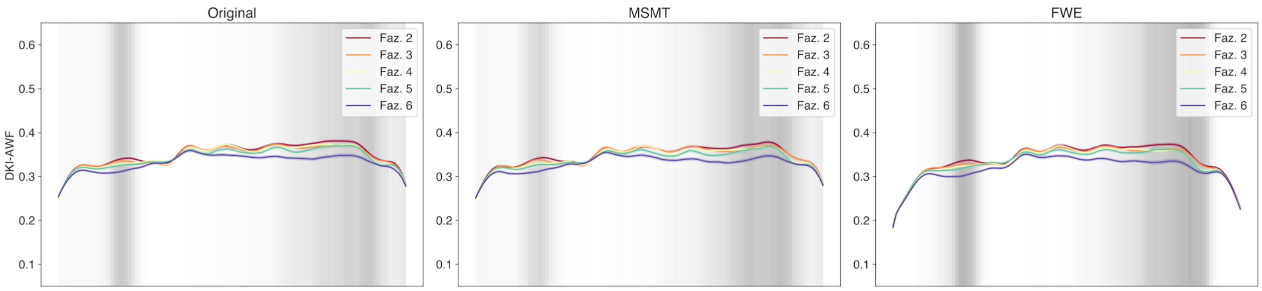

Supplemental Figure 30: Multi-shell Right Inferior Fronto-Occipital Fasiculus DKI-AWF profiles.

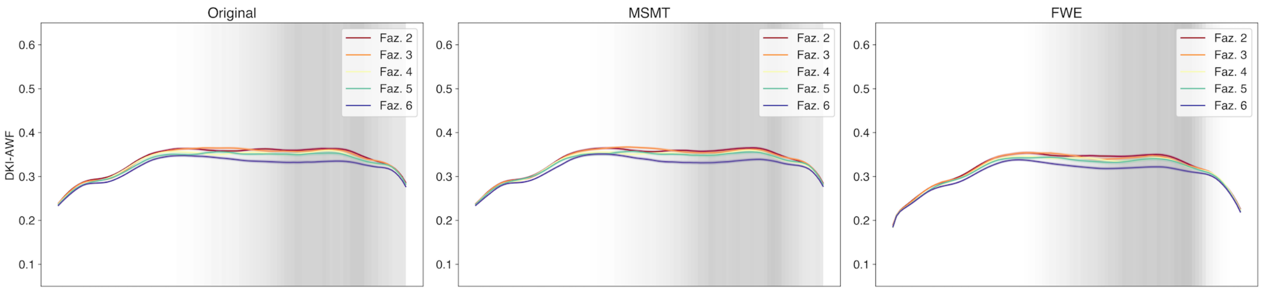

Supplemental Figure 31: Multi-shell Left Inferior Longitudinal Fasiculus DKI-AWF profiles.

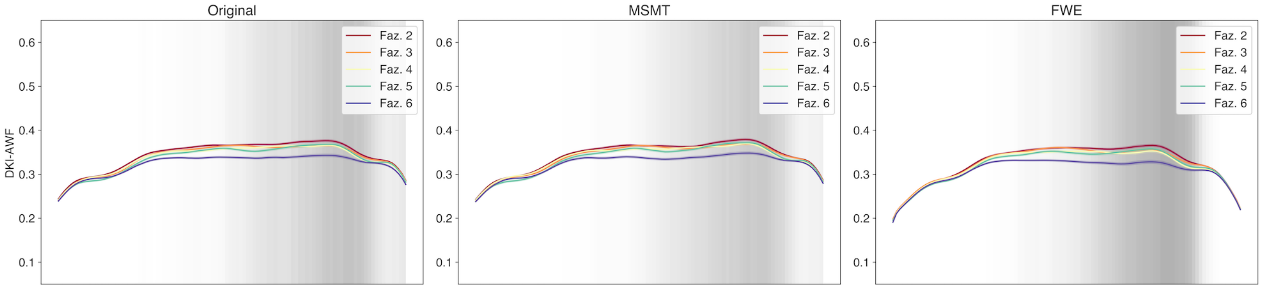

Supplemental Figure 32: Multi-shell Right Inferior Longitudinal Fasiculus DKI-AWF profiles.

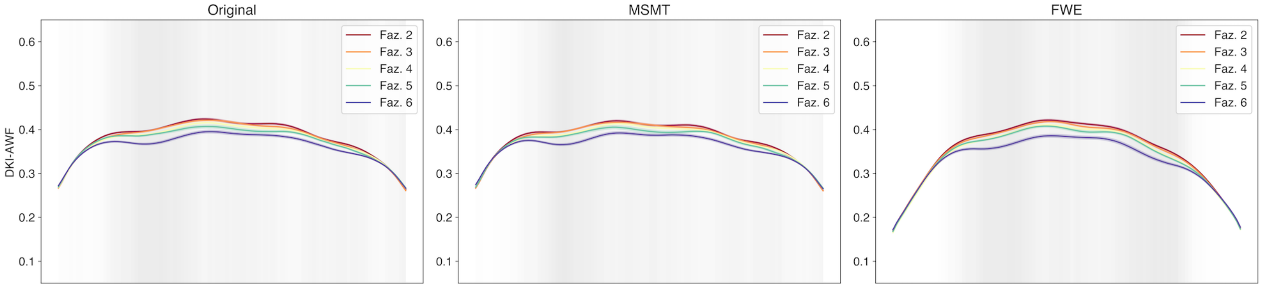

Supplemental Figure 33: Multi-shell Left Superior Longitudinal Fasiculus DKI-AWF profiles.

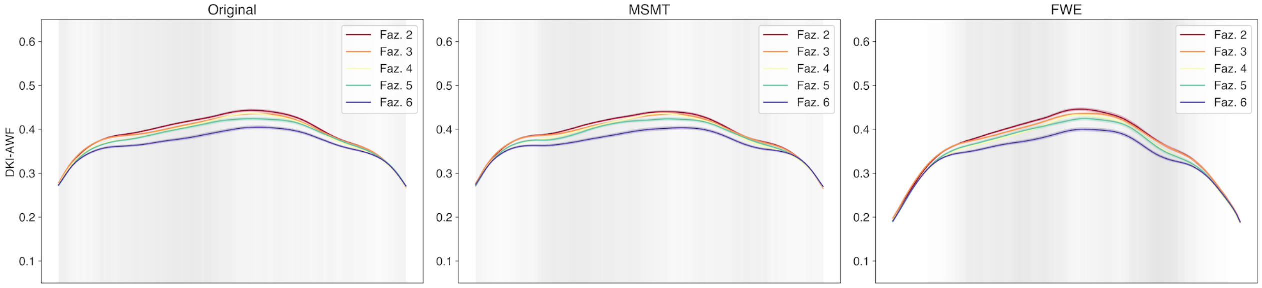

Supplemental Figure 34: Multi-shell Right Superior Longitudinal Fasiculus DKI-AWF profiles.

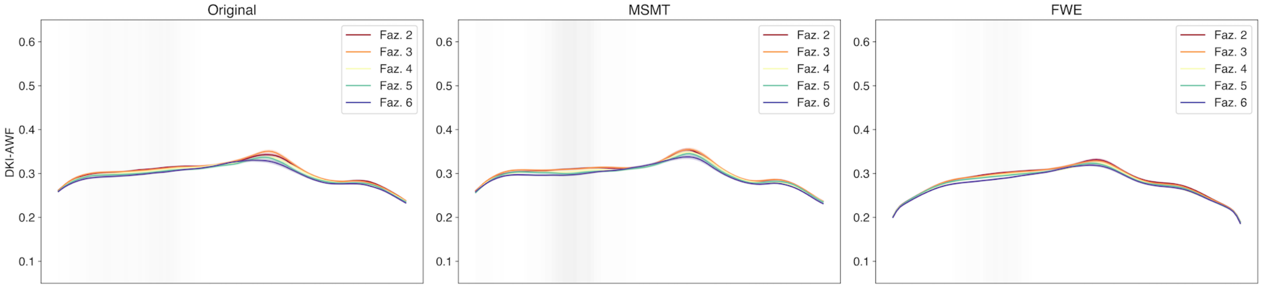

Supplemental Figure 35: Multi-shell Left Uncinate Fasiculus DKI-AWF profiles.

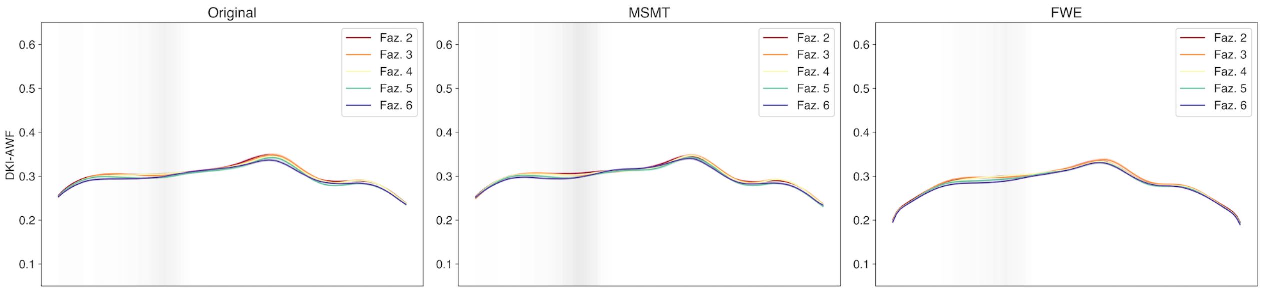

Supplemental Figure 36: Multi-shell Right Uncinate Fasiculus DKI-AWF profiles.

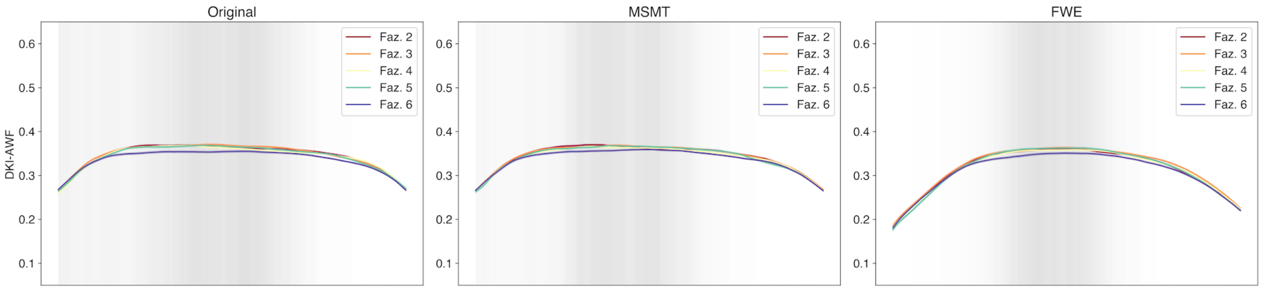

Supplemental Figure 37: Multi-shell Left Vertical Occipital Fasiculus DKI-AWF profiles.

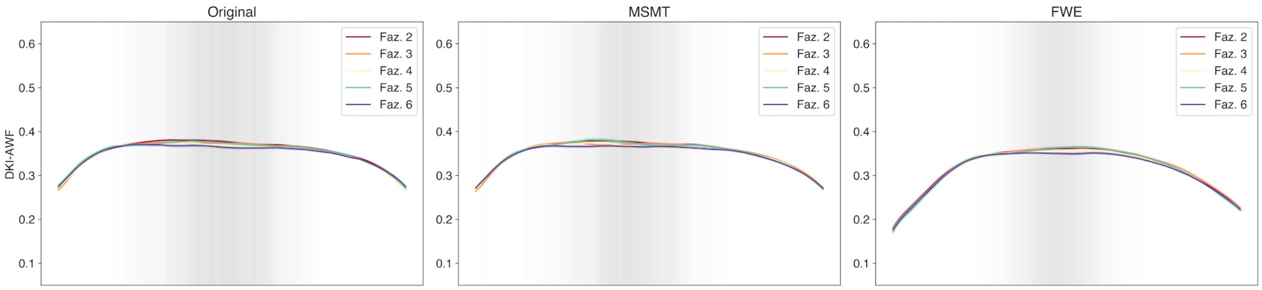

Supplemental Figure 38: Multi-shell Right Vertical Occipital Fasiculus DKI-AWF profiles.

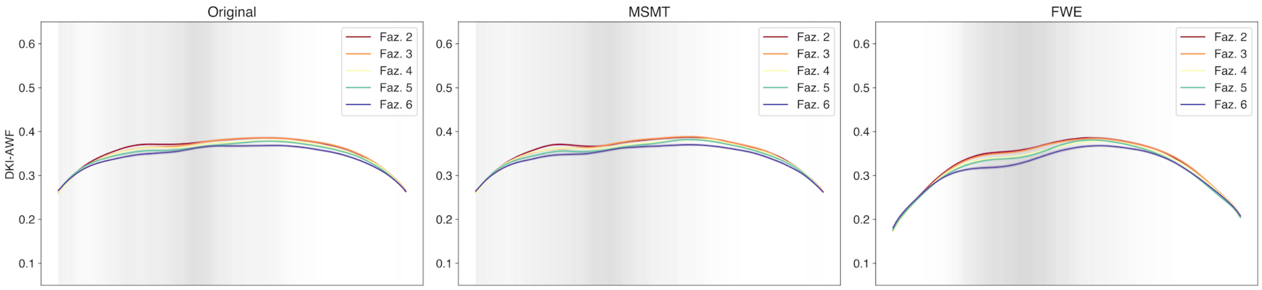

Supplemental Figure 39: Multi-shell Left Posterior Arcuate Fasiculus DKI-AWF profiles.

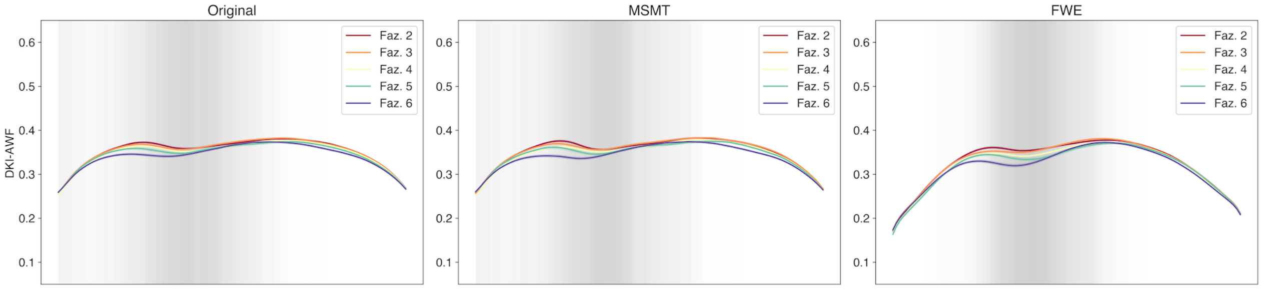

Supplemental Figure 40: Multi-shell Right Posterior Arcuate Fasiculus DKI-AWF profiles.

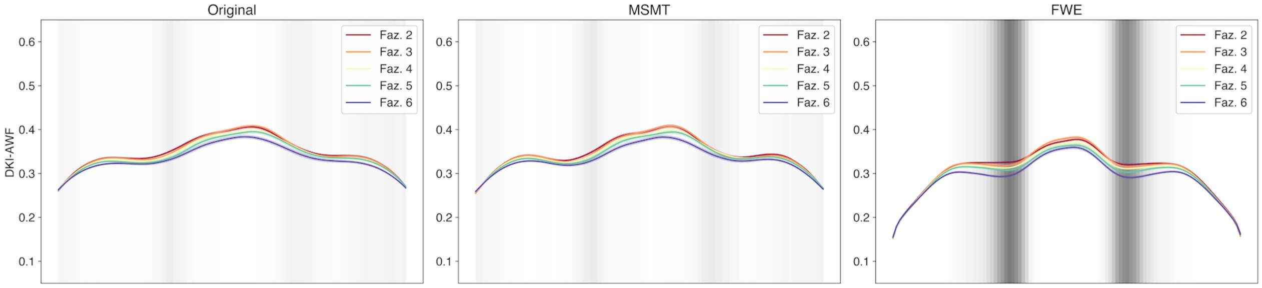

Supplemental Figure 41: Multi-shell Anterior Frontal Callosum DKI-AWF profiles.

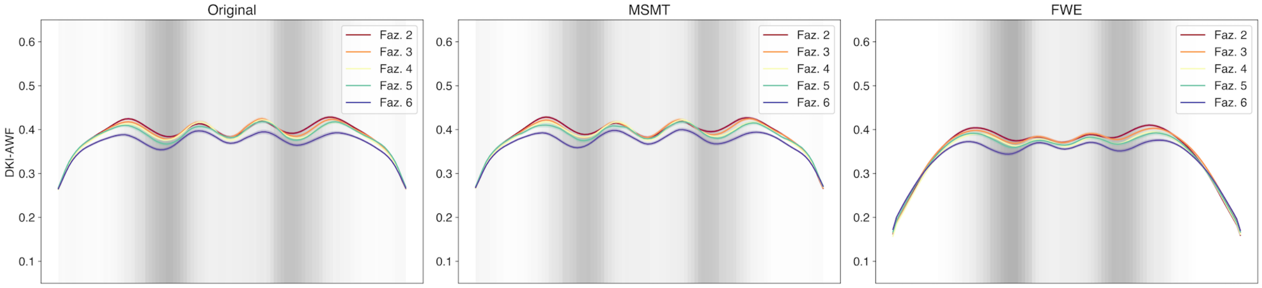

Supplemental Figure 42: Multi-shell Motor Corpus Callosum DKI-AWF profiles.

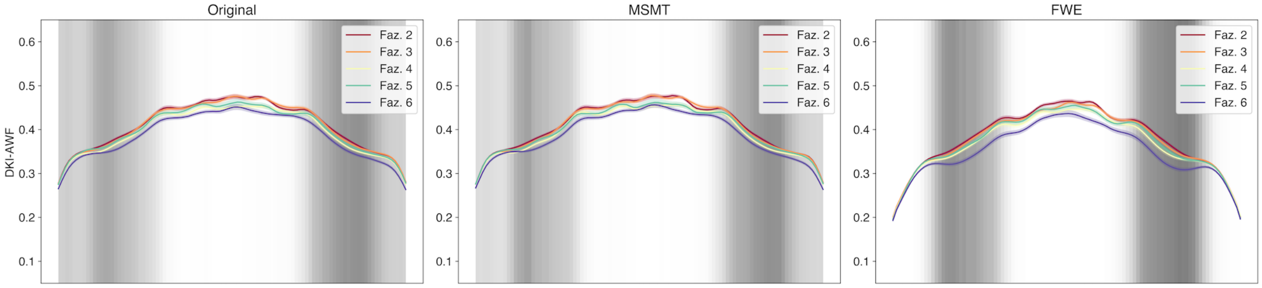

Supplemental Figure 43: Multi-shell Occipital Corpus Callosum DKI-AWF profiles.

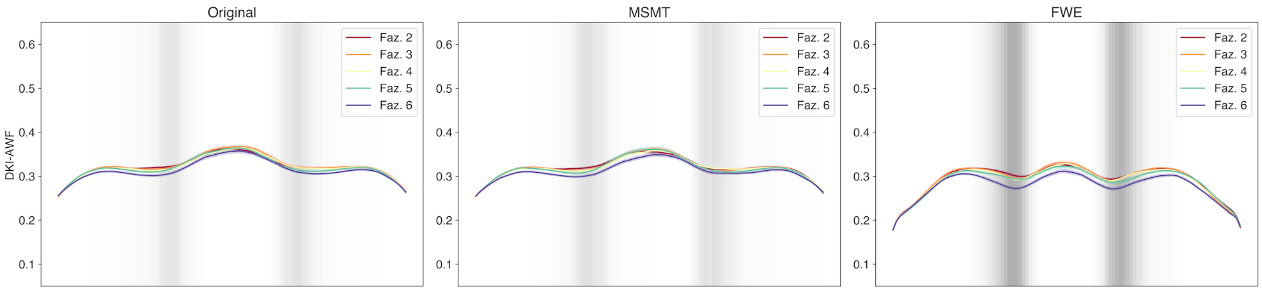

Supplemental Figure 44: Multi-shell Orbital Corpus Callosum DKI-AWF profiles.

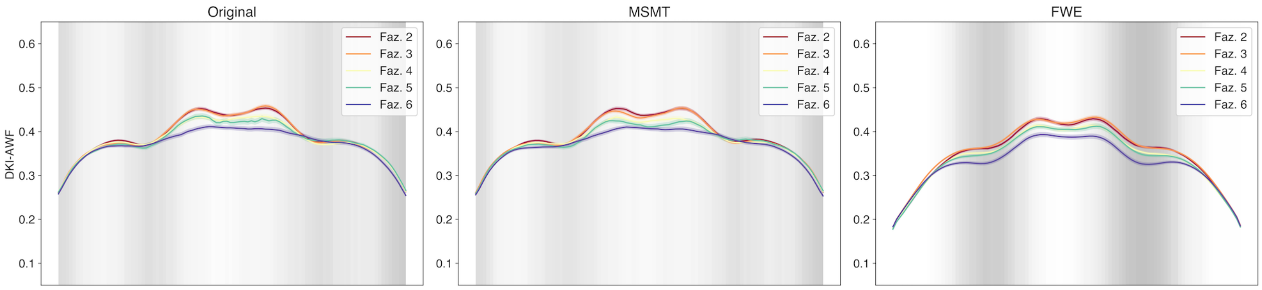

Supplemental Figure 45: Multi-shell Posterior Parietal Corpus Callosum DKI-AWF profiles.

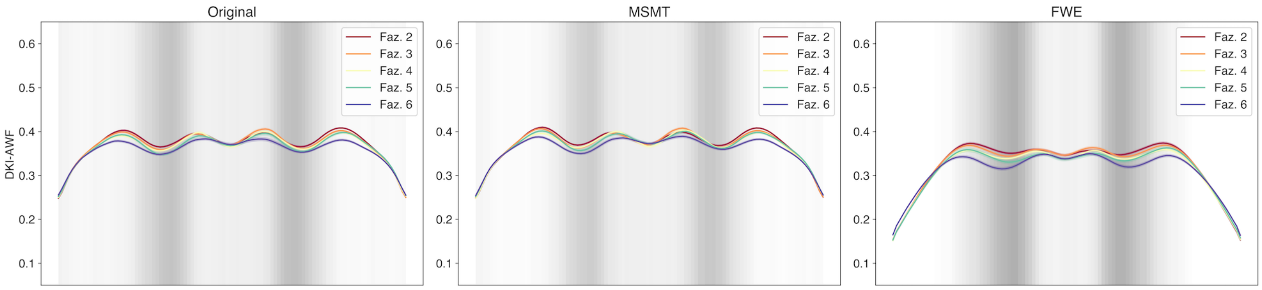

Supplemental Figure 46: Multi-shell Superior Frontal Callosum DKI-AWF profiles.

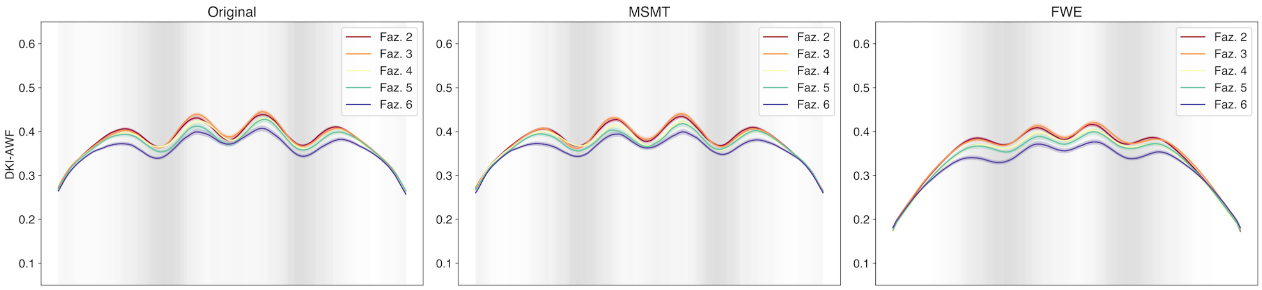

Supplemental Figure 47: Multi-shell Superior Parietal Corpus Callosum DKI-AWF profiles.

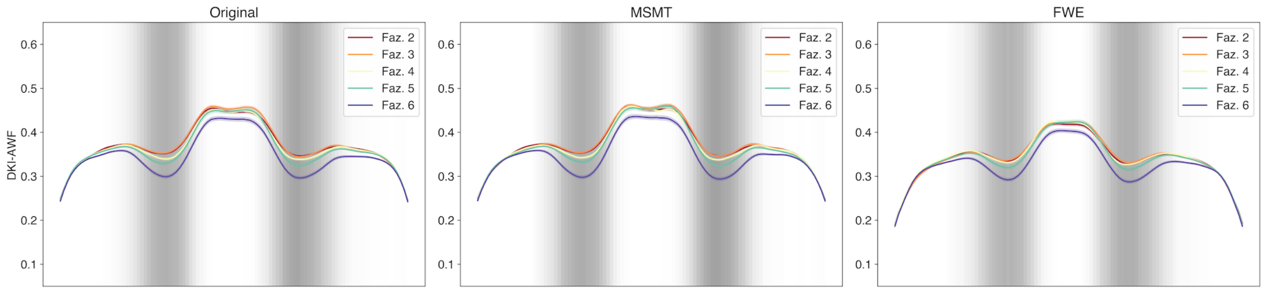

Supplemental Figure 48: Multi-shell Temporal Corpus Callosum DKI-AWF profiles.

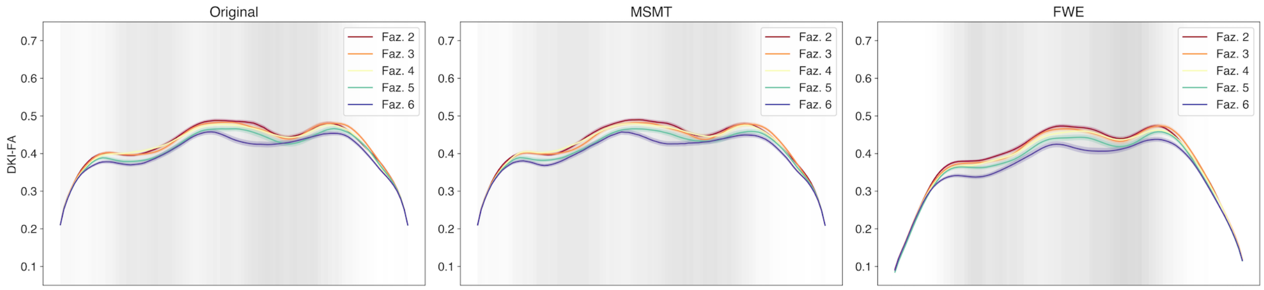

Supplemental Figure 49: Multi-shell Left Arcuate Fasiculus DKI-FA profiles.

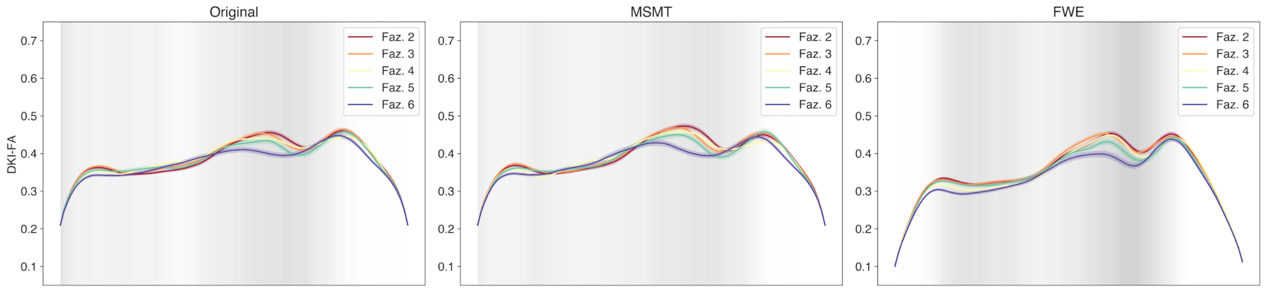

Supplemental Figure 50: Multi-shell Right Arcuate Fasiculus DKI-FA profiles.

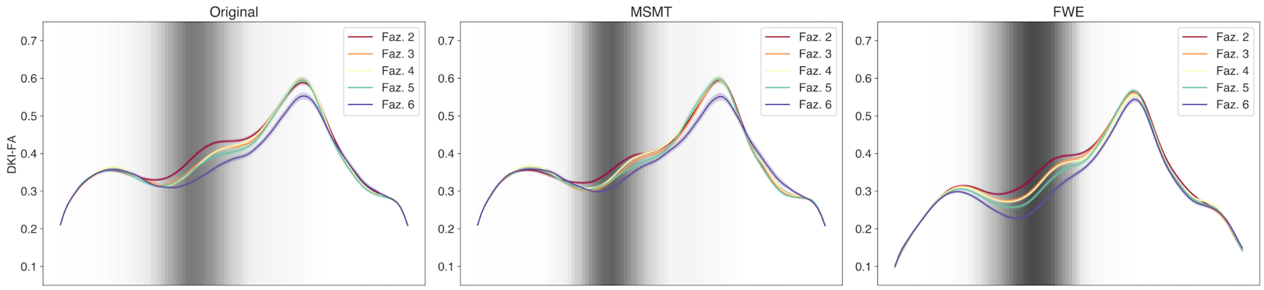

Supplemental Figure 51: Multi-shell Left Anterior Thalamic Radiation DKI-FA profiles.

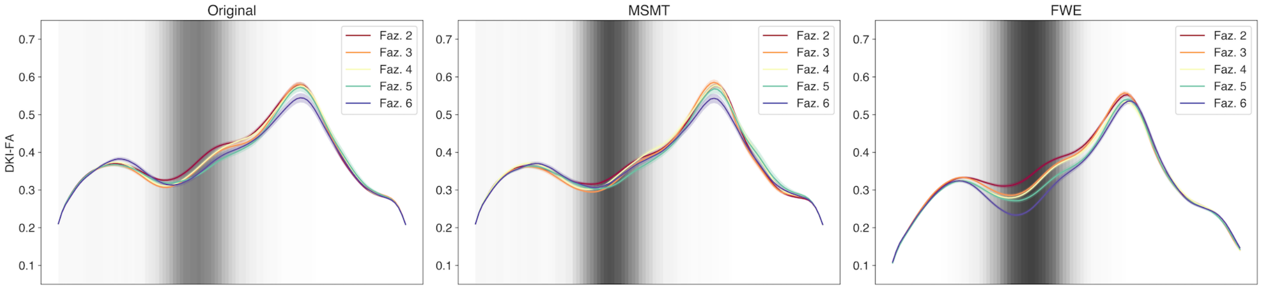

Supplemental Figure 52: Multi-shell Right Anterior Thalamic Radiation DKI-FA profiles.

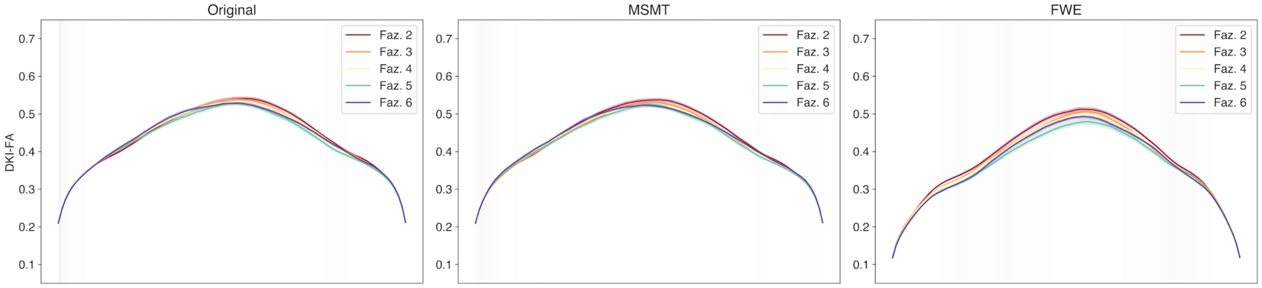

Supplemental Figure 53: Multi-shell Left Cingulum Cingulate DKI-FA profiles.

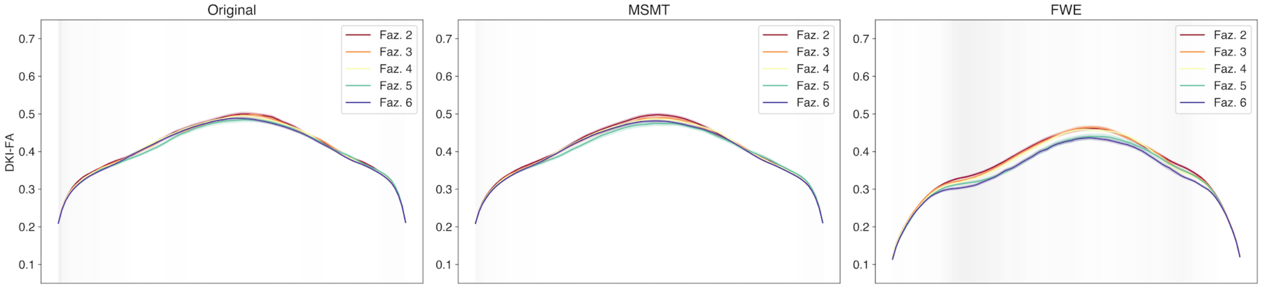

Supplemental Figure 54: Multi-shell Right Cingulum Cingulate DKI-FA profiles.

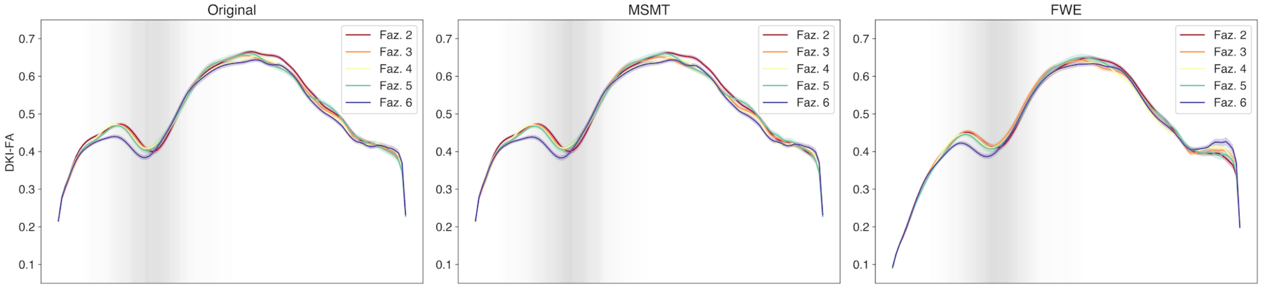

Supplemental Figure 55: Multi-shell Left Corticospinal Tract DKI-FA profiles.

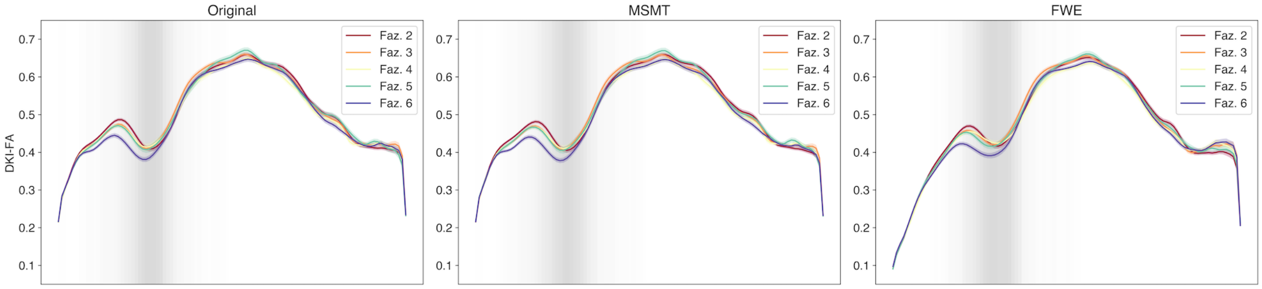

Supplemental Figure 56: Multi-shell Right Corticospinal Tract DKI-FA profiles.

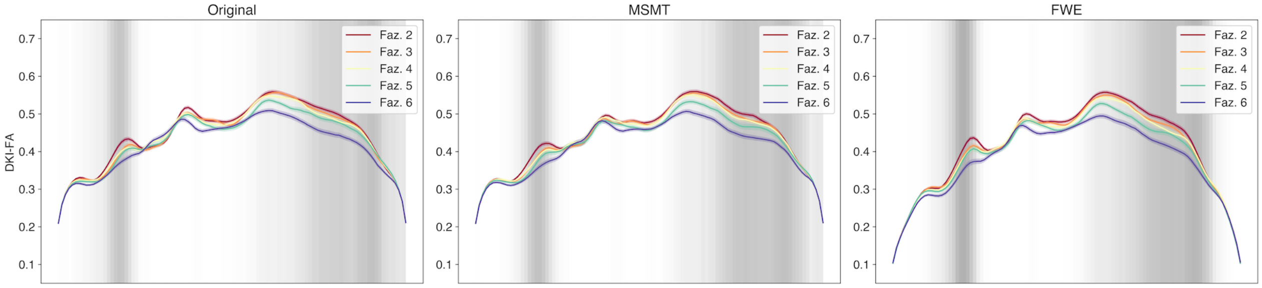

Supplemental Figure 57: Multi-shell Left Inferior Fronto-Occipital Fasiculus DKI-FA profiles.

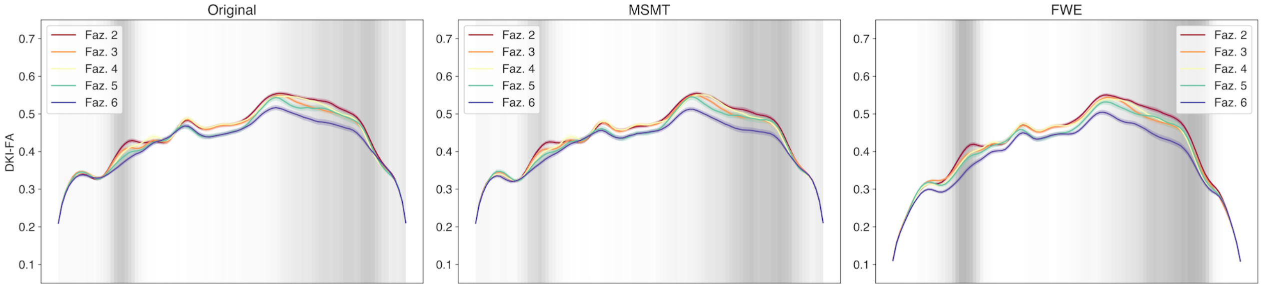

Supplemental Figure 58: Multi-shell Right Inferior Fronto-Occipital Fasiculus DKI-FA profiles.

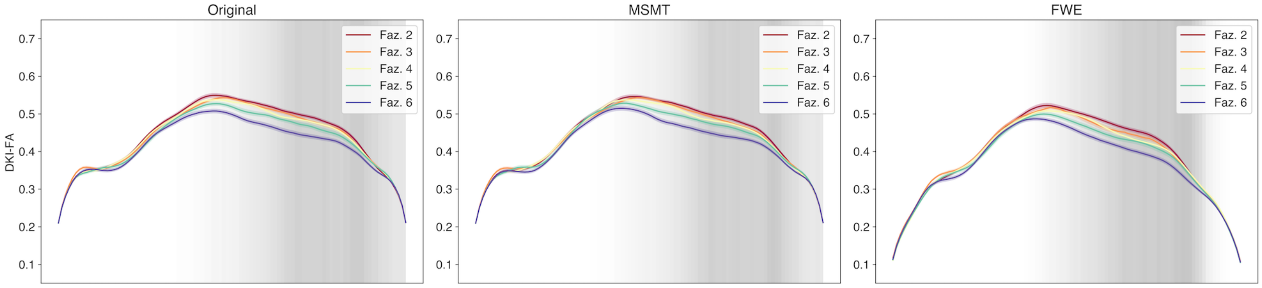

Supplemental Figure 59: Multi-shell Left Inferior Longitudinal Fasiculus DKI-FA profiles.

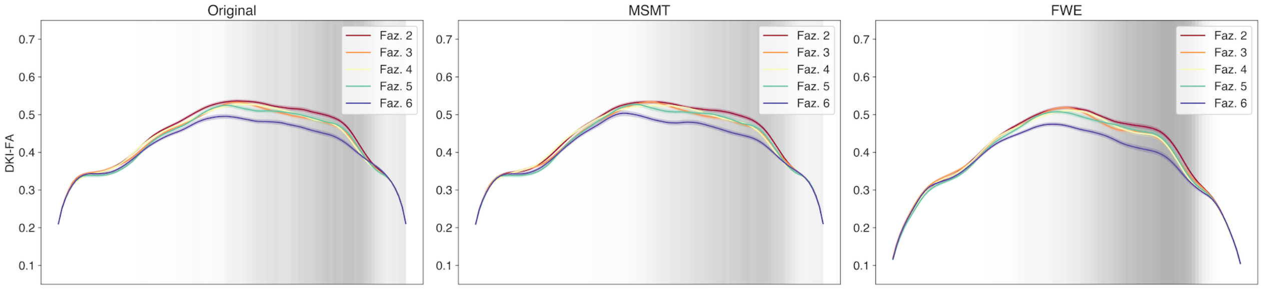

Supplemental Figure 60: Multi-shell Right Inferior Longitudinal Fasiculus DKI-FA profiles.

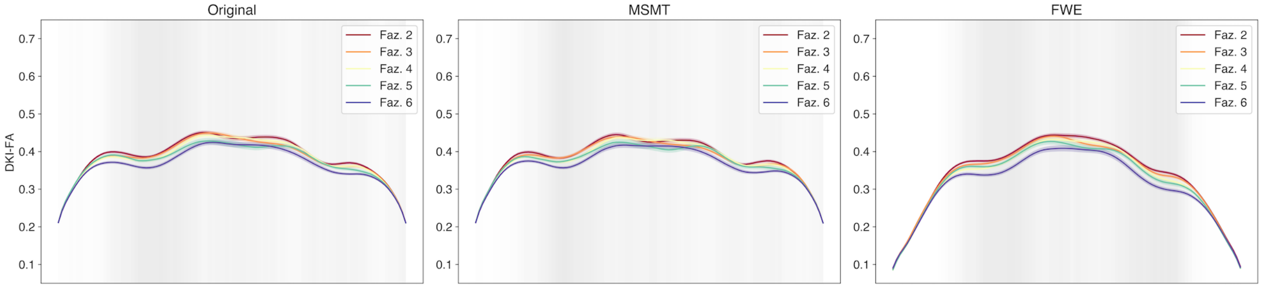

Supplemental Figure 61: Multi-shell Left Superior Longitudinal Fasiculus DKI-FA profiles.

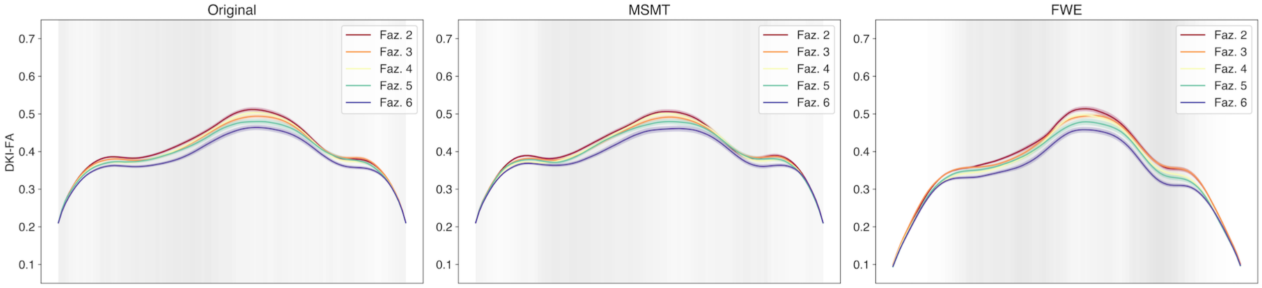

Supplemental Figure 62: Multi-shell Right Superior Longitudinal Fasiculus DKI-FA profiles.

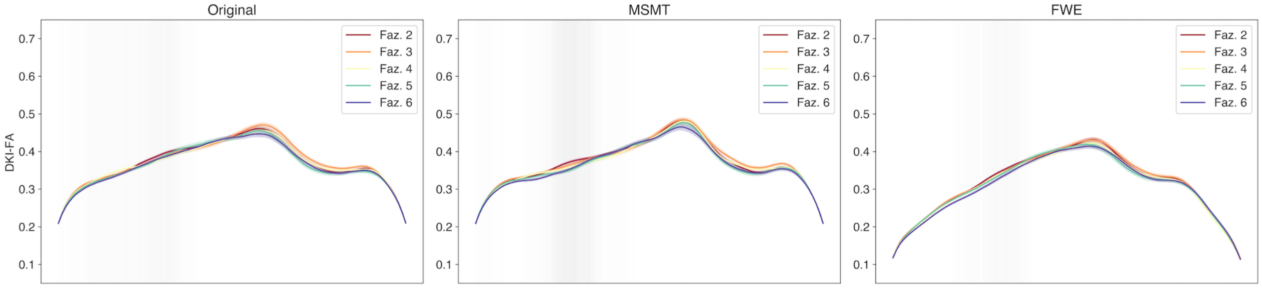

Supplemental Figure 63: Multi-shell Left Uncinate Fasiculus DKI-FA profiles.

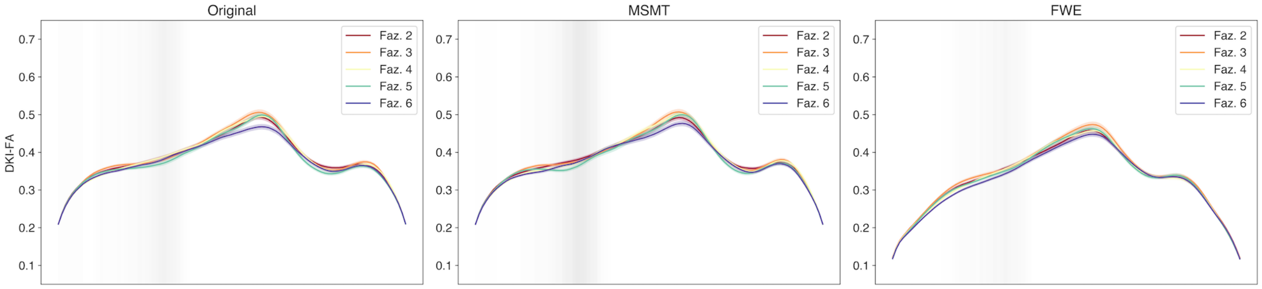

Supplemental Figure 64: Multi-shell Right Uncinate Fasiculus DKI-FA profiles.

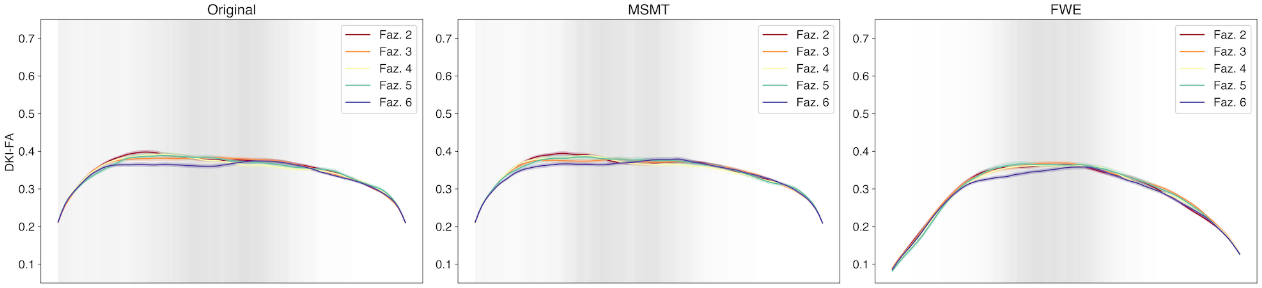

Supplemental Figure 65: Multi-shell Left Vertical Occipital Fasiculus DKI-FA profiles.

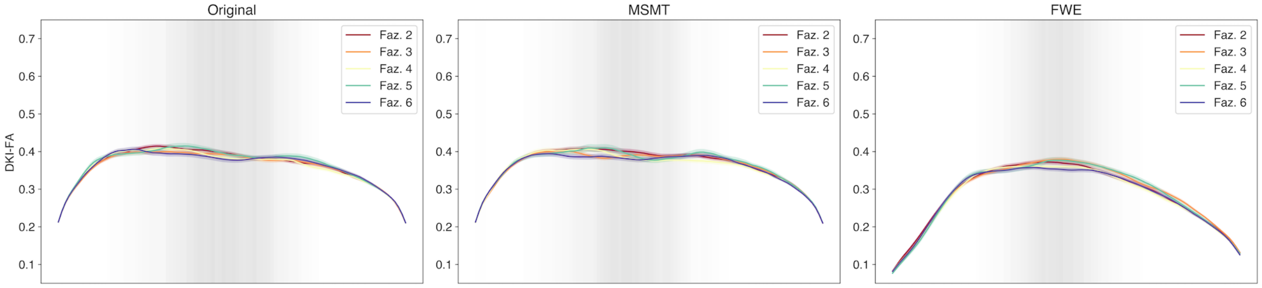

Supplemental Figure 66: Multi-shell Right Vertical Occipital Fasiculus DKI-FA profiles.

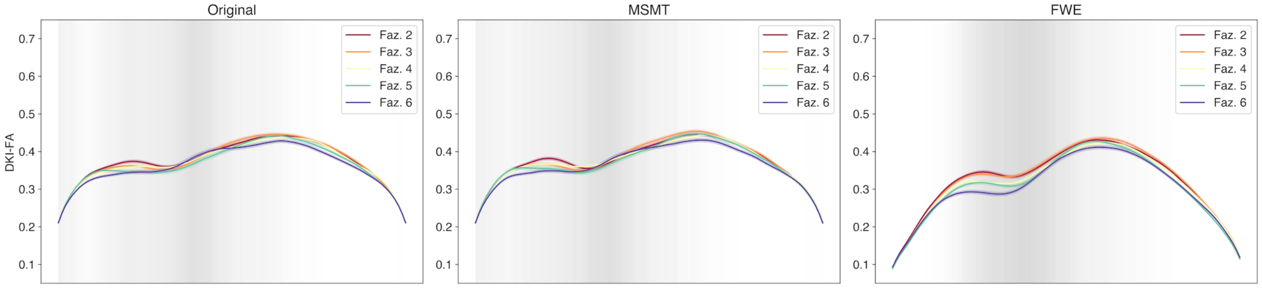

Supplemental Figure 67: Multi-shell Left Posterior Arcuate Fasiculus DKI-FA profiles.

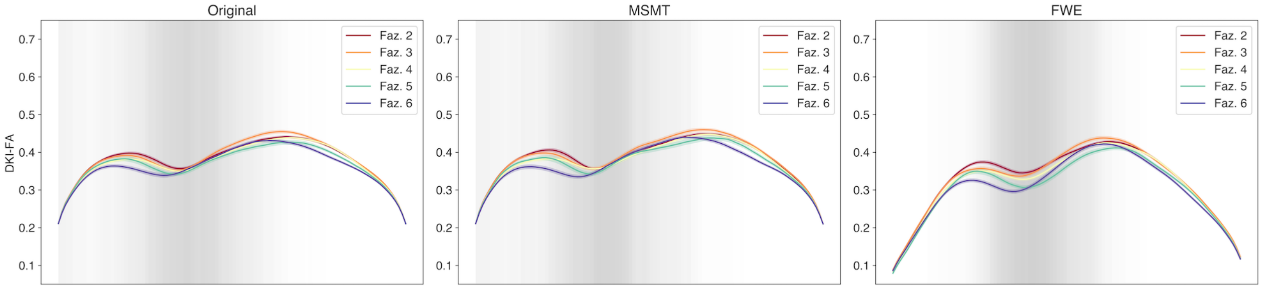

Supplemental Figure 68: Multi-shell Right Posterior Arcuate Fasiculus DKI-FA profiles.

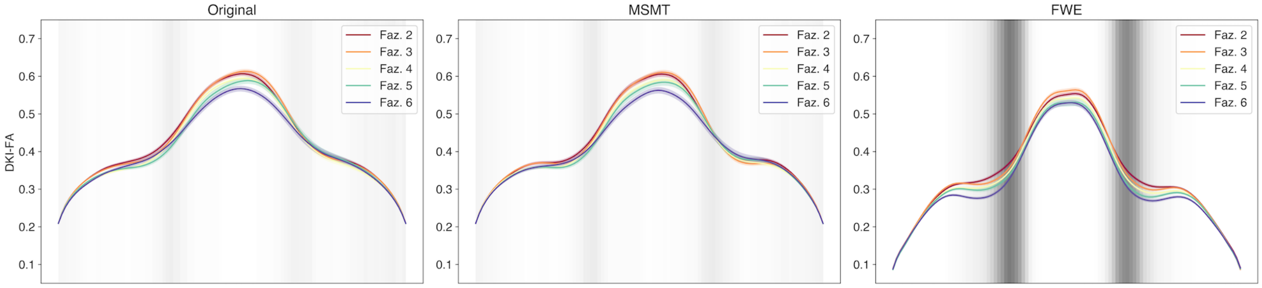

Supplemental Figure 69: Multi-shell Anterior Frontal Callosum DKI-FA profiles.

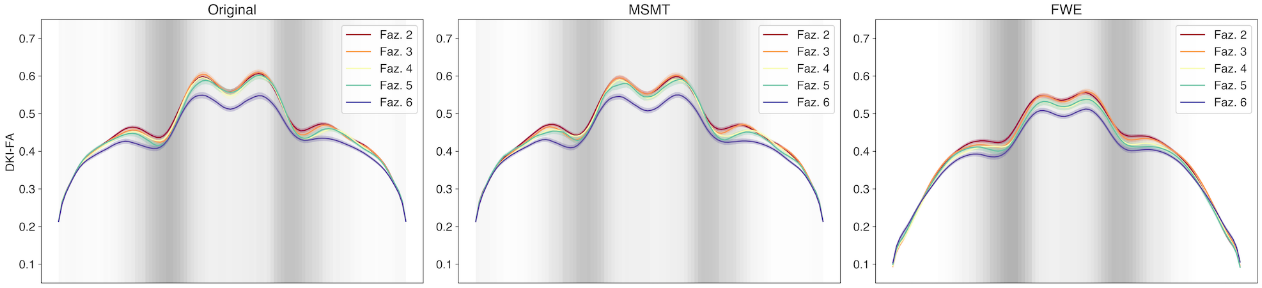

Supplemental Figure 70: Multi-shell Motor Corpus Callosum DKI-FA profiles.

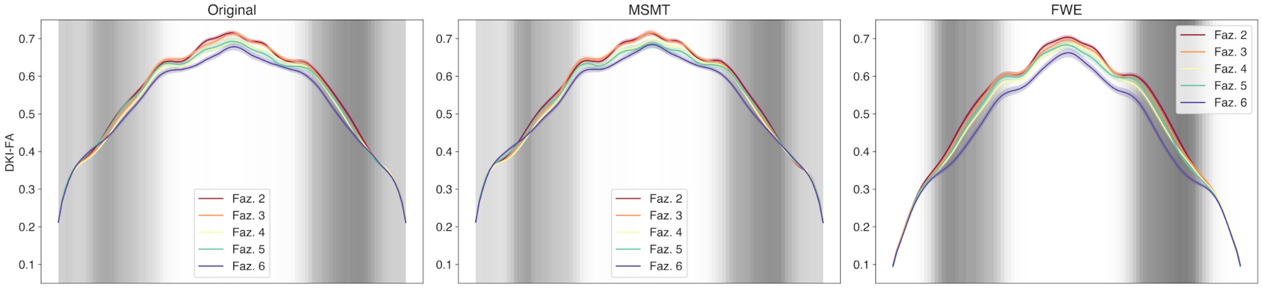

Supplemental Figure 71: Multi-shell Occipital Corpus Callosum DKI-FA profiles.

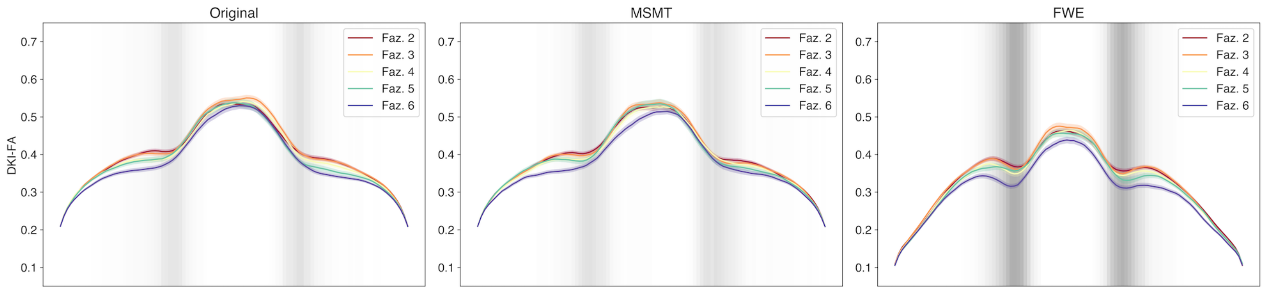

Supplemental Figure 72: Multi-shell Orbital Corpus Callosum DKI-FA profiles.

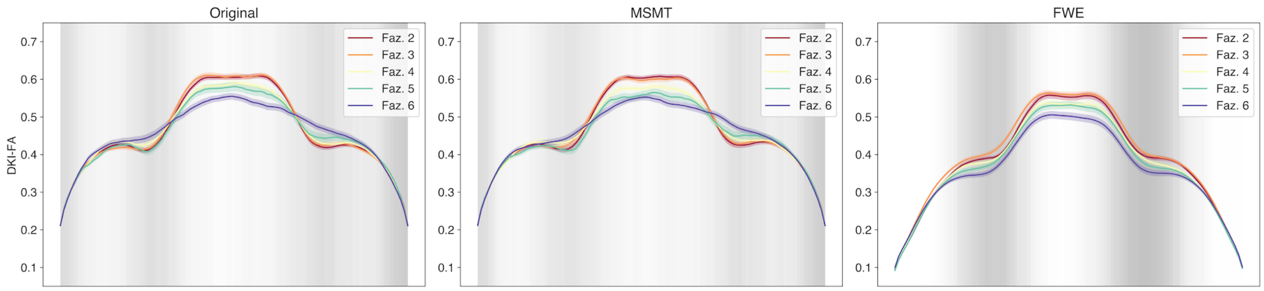

Supplemental Figure 73: Multi-shell Posterior Parietal Corpus Callosum DKI-FA profiles.

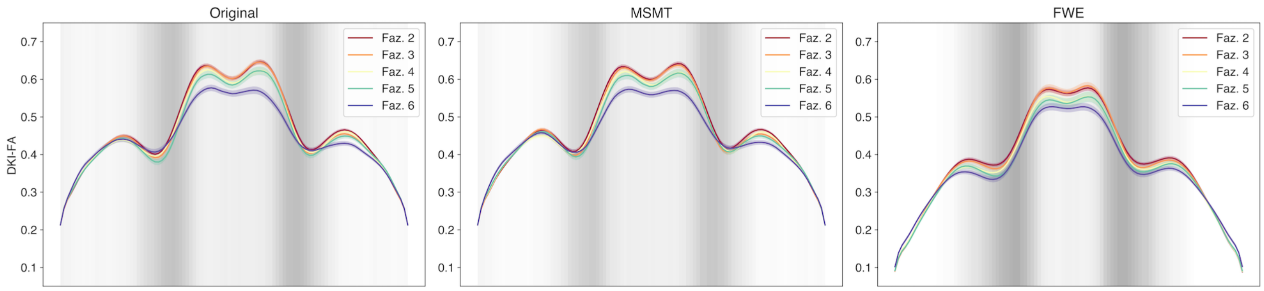

Supplemental Figure 74: Multi-shell Superior Frontal Callosum DKI-FA profiles.

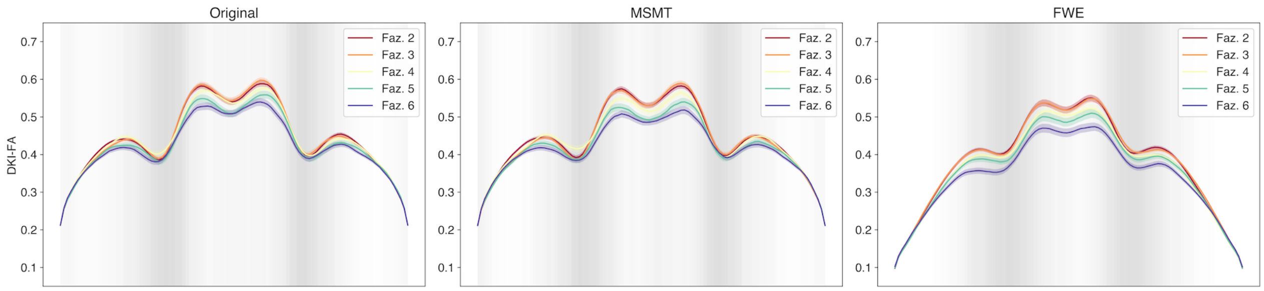

Supplemental Figure 75: Multi-shell Superior Parietal Corpus Callosum DKI-FA profiles.

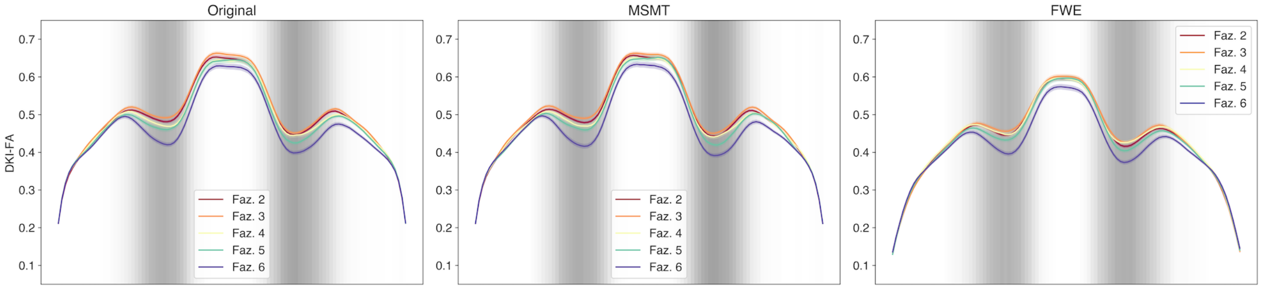

Supplemental Figure 76: Multi-shell Temporal Corpus Callosum DKI-FA profiles.

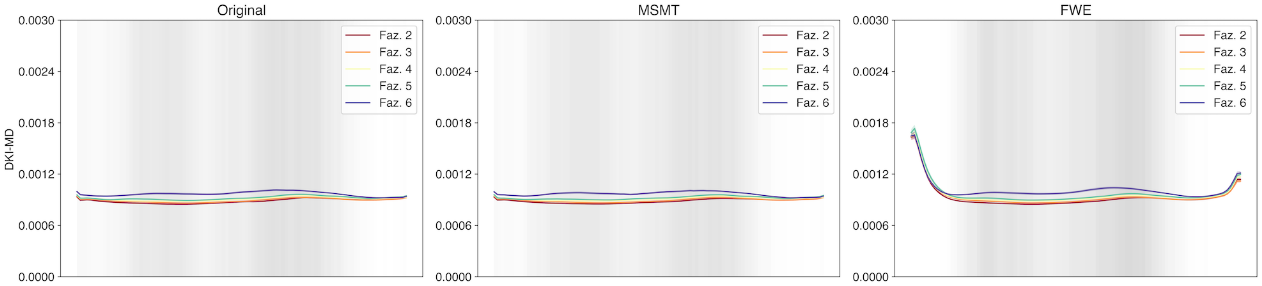

Supplemental Figure 77: Multi-shell Left Arcuate Fasiculus DKI-MD profiles.

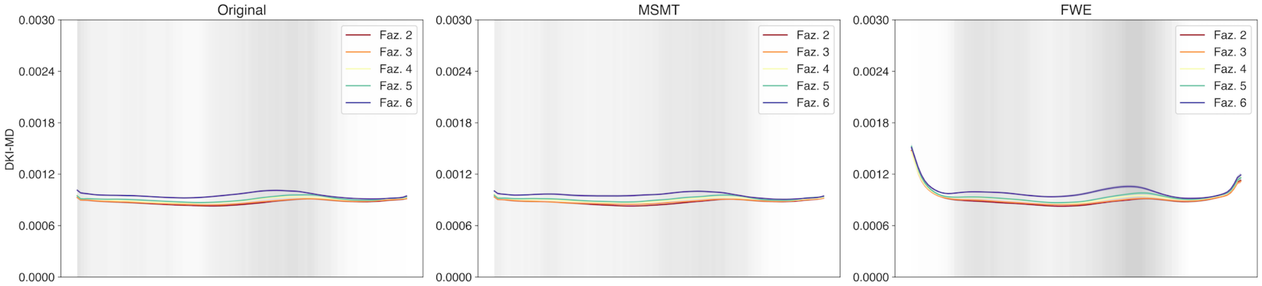

Supplemental Figure 78: Multi-shell Right Arcuate Fasiculus DKI-MD profiles.

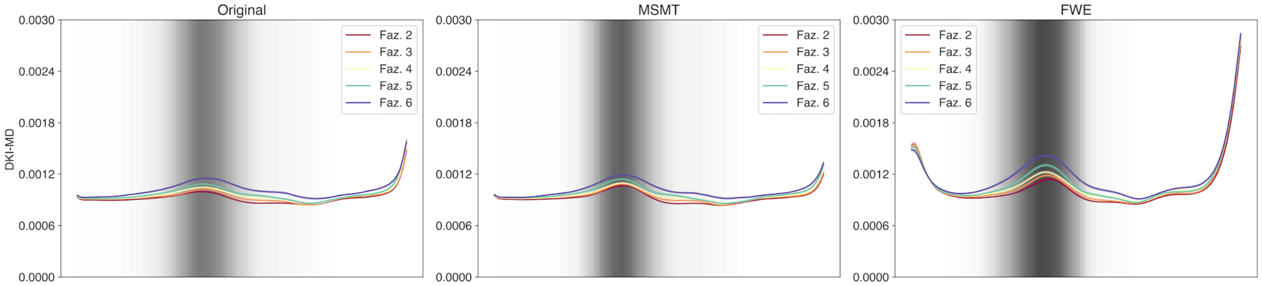

Supplemental Figure 79: Multi-shell Left Anterior Thalamic Radiation DKI-MD profiles.

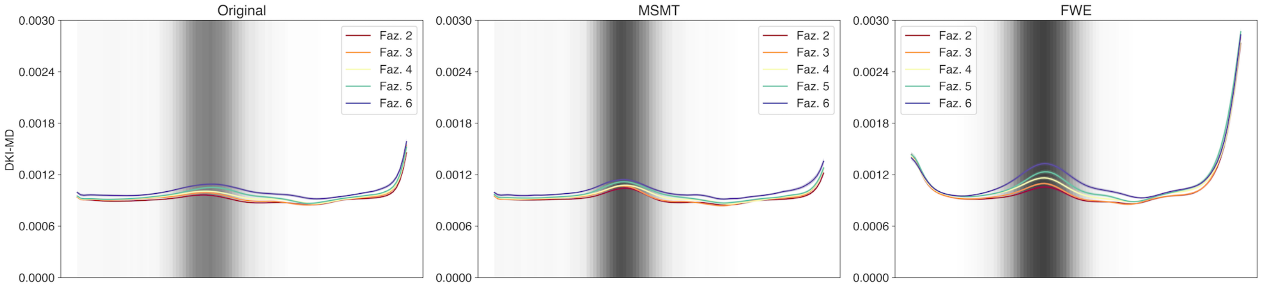

Supplemental Figure 80: Multi-shell Right Anterior Thalamic Radiation DKI-MD profiles.

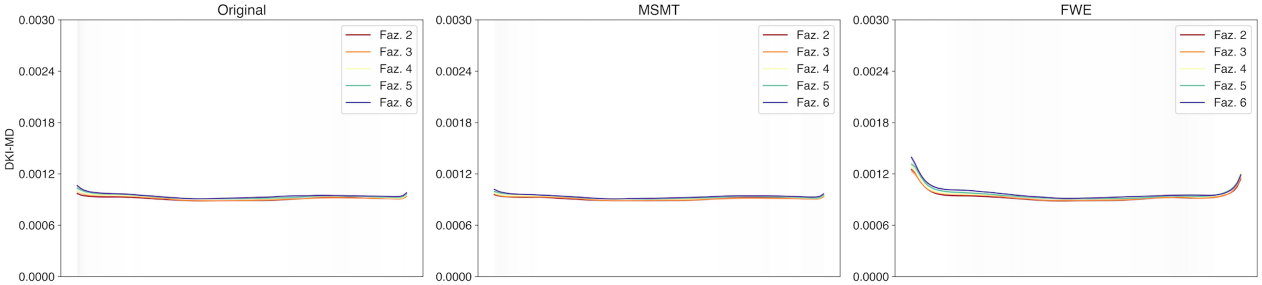

Supplemental Figure 81: Multi-shell Left Cingulum Cingulate DKI-MD profiles.

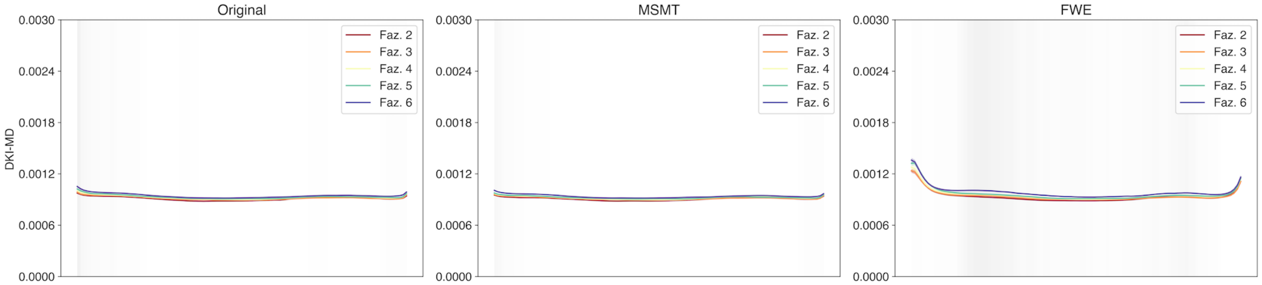

Supplemental Figure 82: Multi-shell Right Cingulum Cingulate DKI-MD profiles.

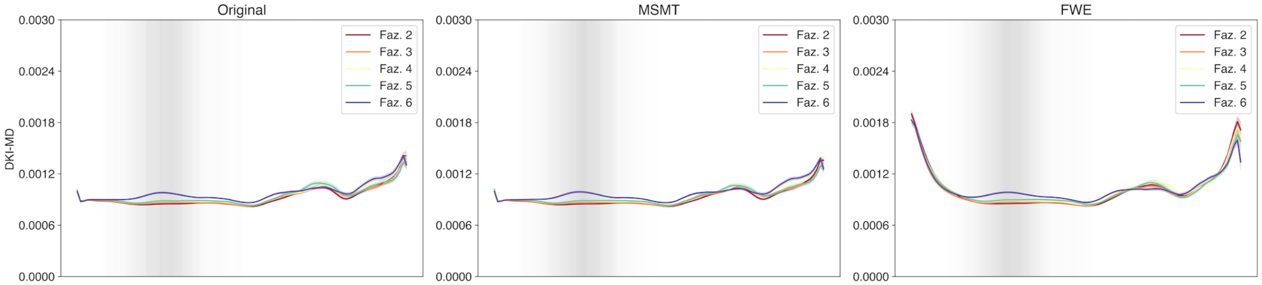

Supplemental Figure 83: Multi-shell Left Corticospinal Tract DKI-MD profiles.

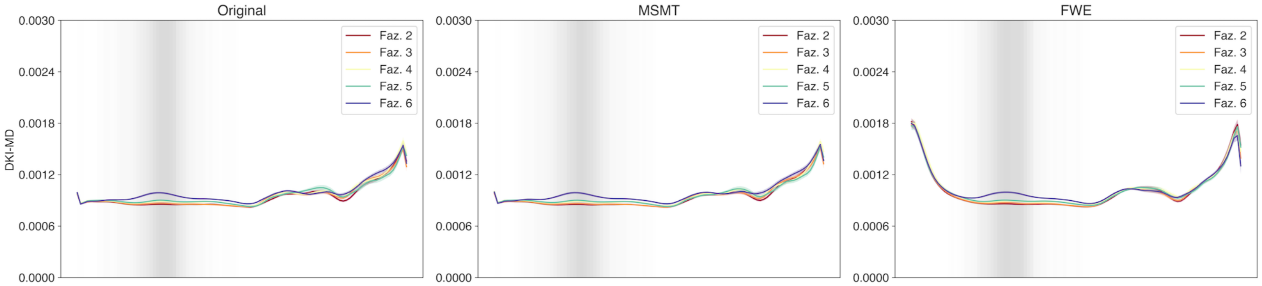

Supplemental Figure 84: Multi-shell Right Corticospinal Tract DKI-MD profiles.

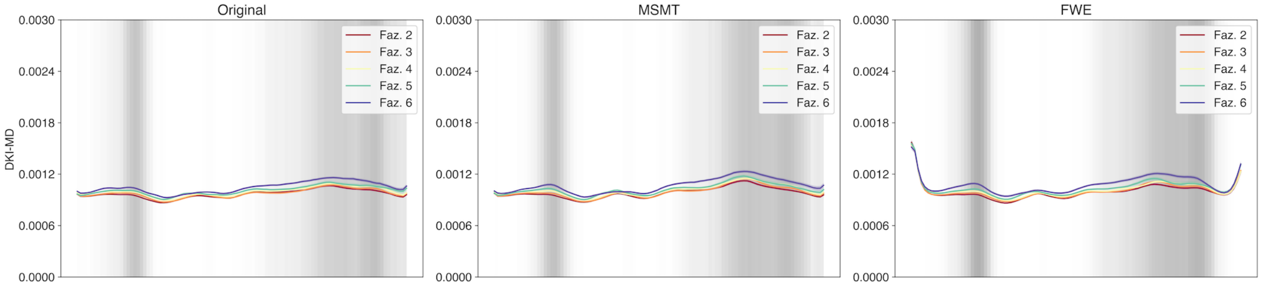

Supplemental Figure 85: Multi-shell Left Inferior Fronto-Occipital Fasiculus DKI-MD profiles.

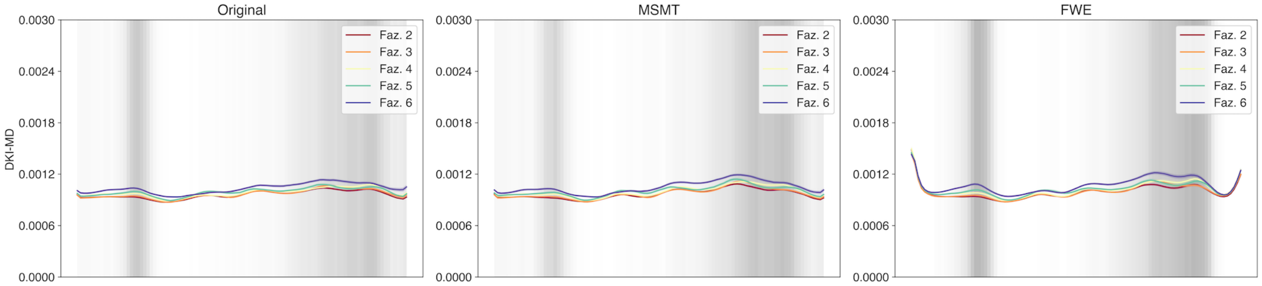

Supplemental Figure 86: Multi-shell Right Inferior Fronto-Occipital Fasiculus DKI-MD profiles.

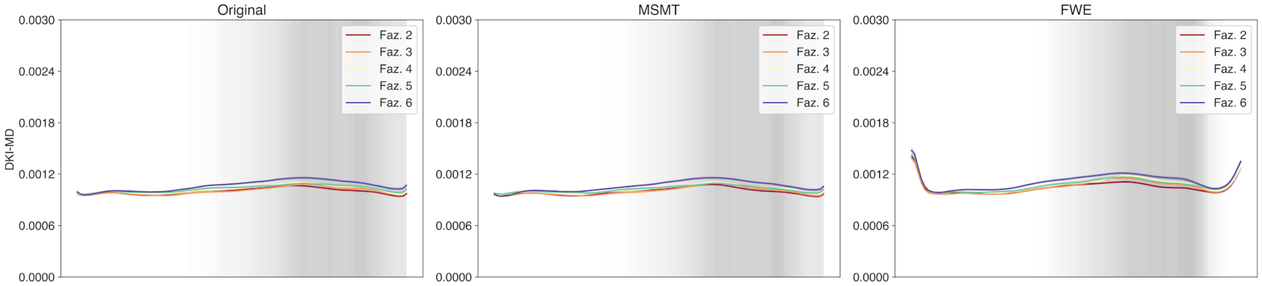

Supplemental Figure 87: Multi-shell Left Inferior Longitudinal Fasiculus DKI-MD profiles.

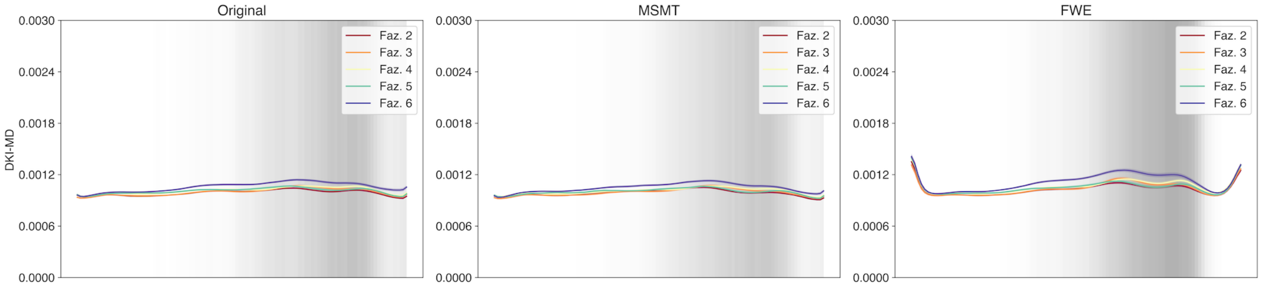

Supplemental Figure 88: Multi-shell Right Inferior Longitudinal Fasiculus DKI-MD profiles.

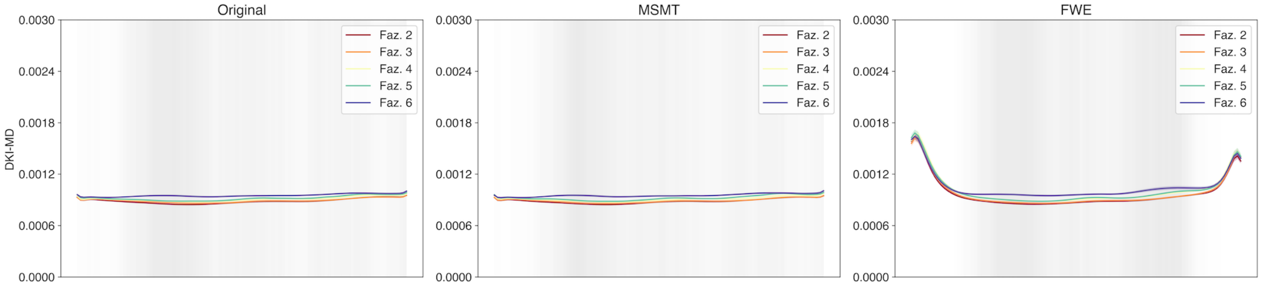

Supplemental Figure 89: Multi-shell Left Superior Longitudinal Fasiculus DKI-MD profiles.

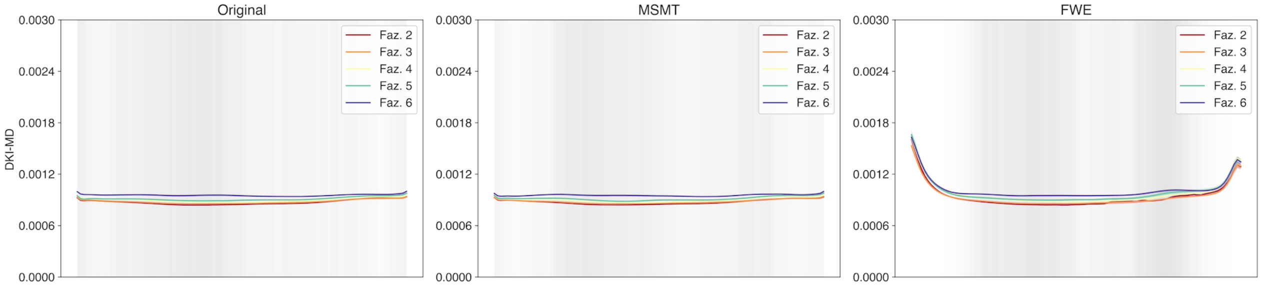

Supplemental Figure 90: Multi-shell Right Superior Longitudinal Fasiculus DKI-MD profiles.

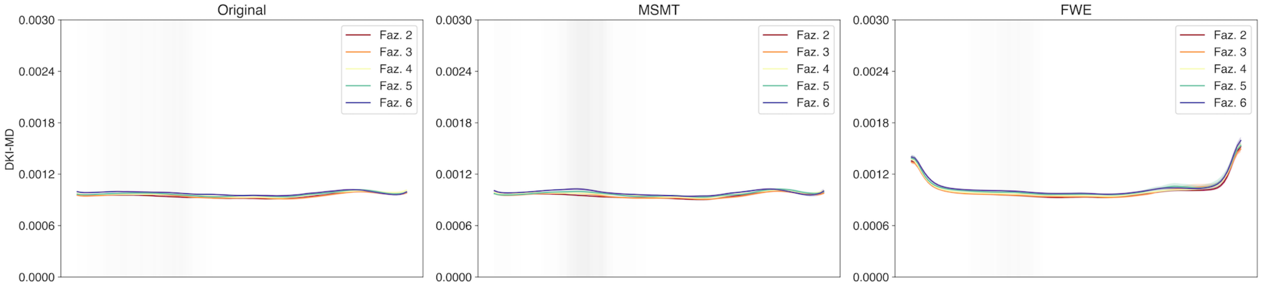

Supplemental Figure 91: Multi-shell Left Uncinate Fasiculus DKI-MD profiles.

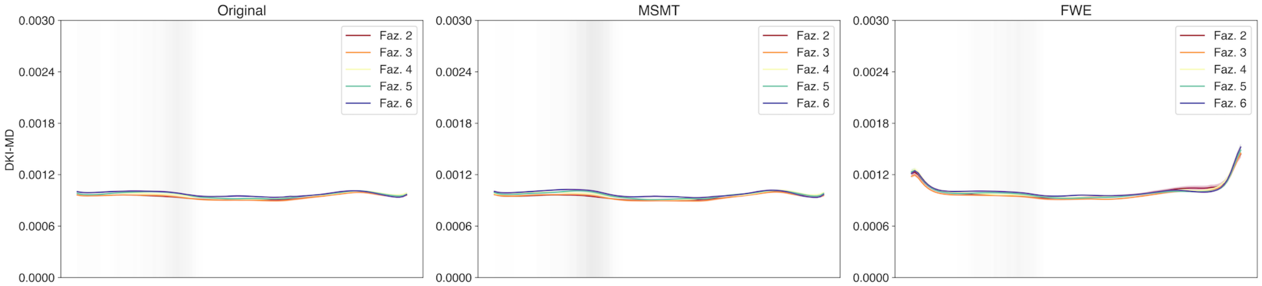

Supplemental Figure 92: Multi-shell Right Uncinate Fasiculus DKI-MD profiles.

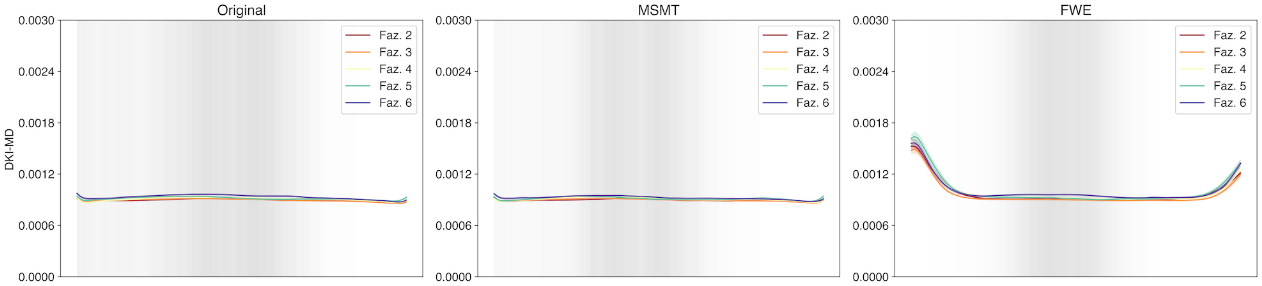

Supplemental Figure 93: Multi-shell Left Vertical Occipital Fasiculus DKI-MD profiles.

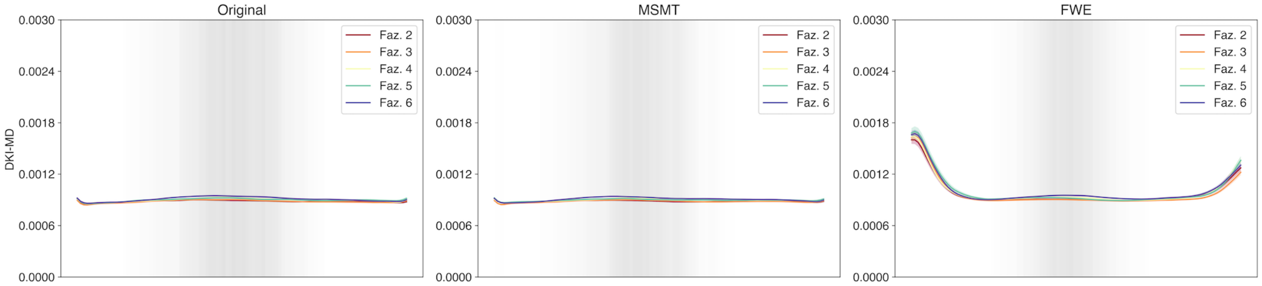

Supplemental Figure 94: Multi-shell Right Vertical Occipital Fasiculus DKI-MD profiles.

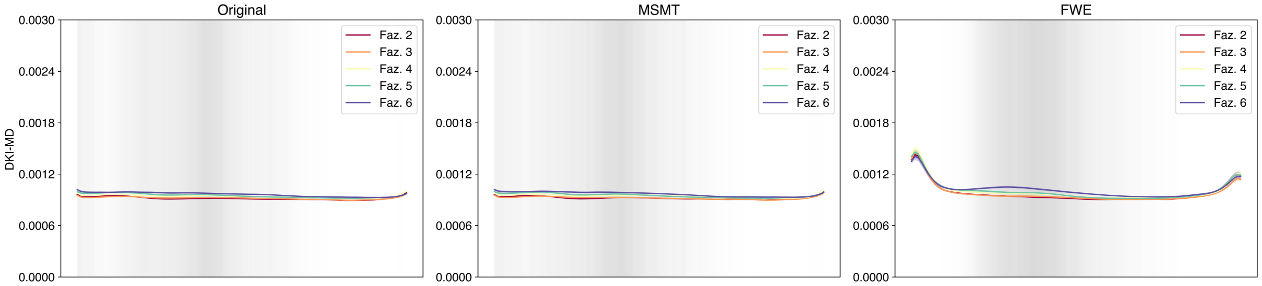

Supplemental Figure 95: Multi-shell Left Posterior Arcuate Fasiculus DKI-MD profiles.

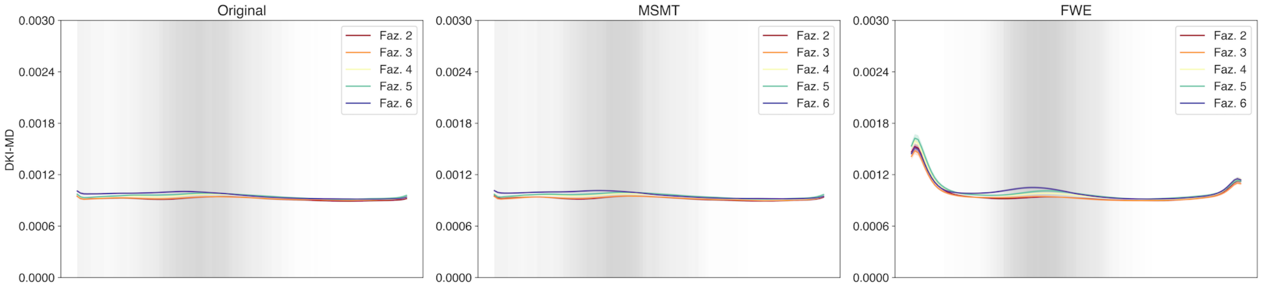

Supplemental Figure 96: Multi-shell Right Posterior Arcuate Fasiculus DKI-MD profiles.

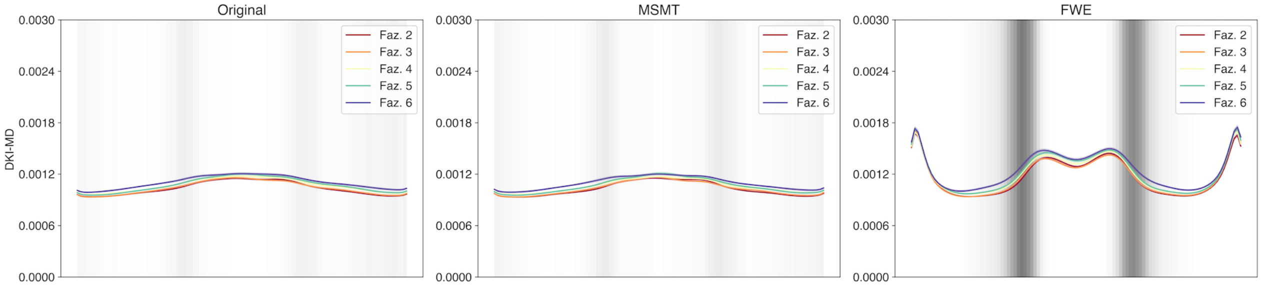

Supplemental Figure 97: Multi-shell Anterior Frontal Callosum DKI-MD profiles.

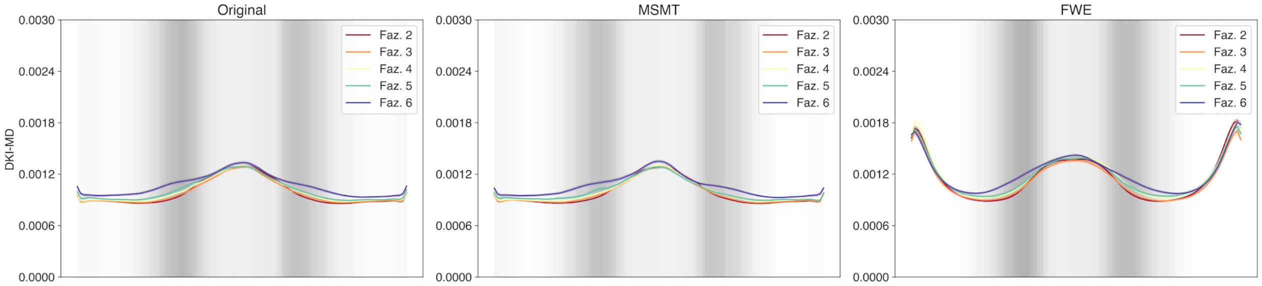

Supplemental Figure 98: Multi-shell Motor Corpus Callosum DKI-MD profiles.

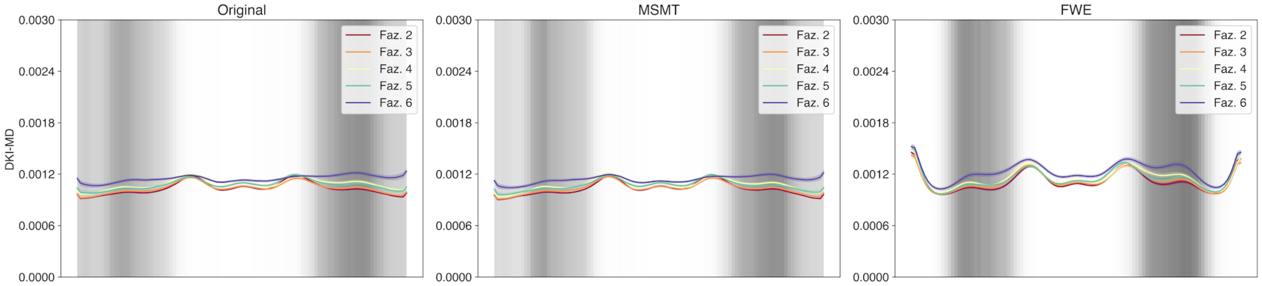

Supplemental Figure 99: Multi-shell Occipital Corpus Callosum DKI-MD profiles.

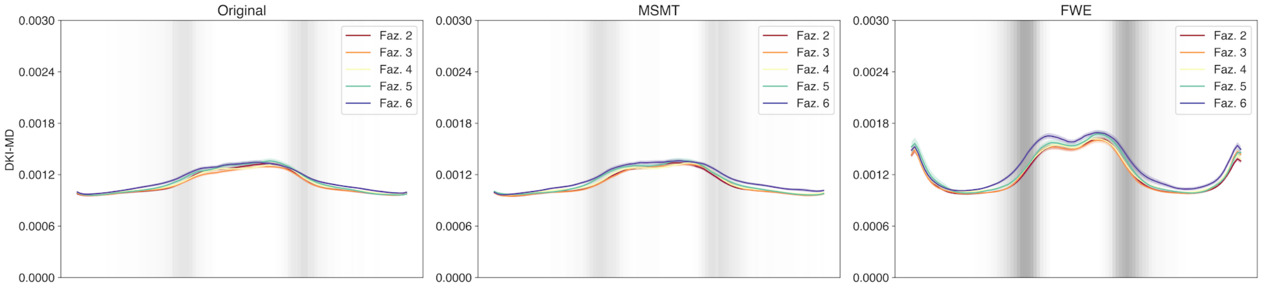

Supplemental Figure 100: Multi-shell Orbital Corpus Callosum DKI-MD profiles.

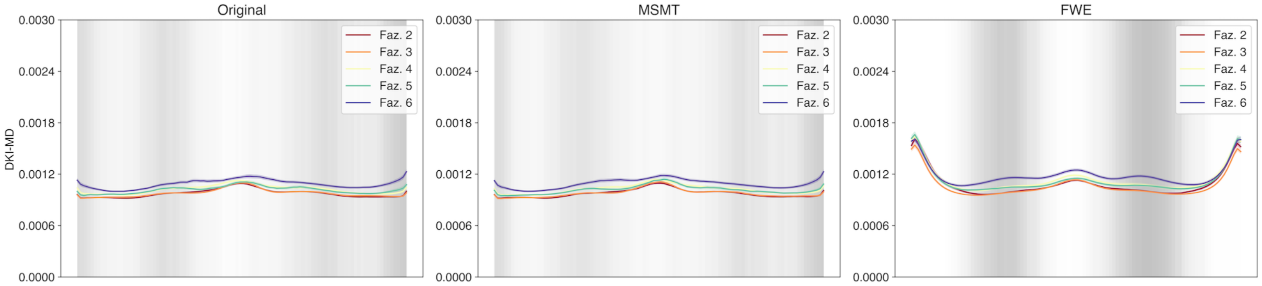

Supplemental Figure 101: Multi-shell Posterior Parietal Corpus Callosum DKI-MD profiles.

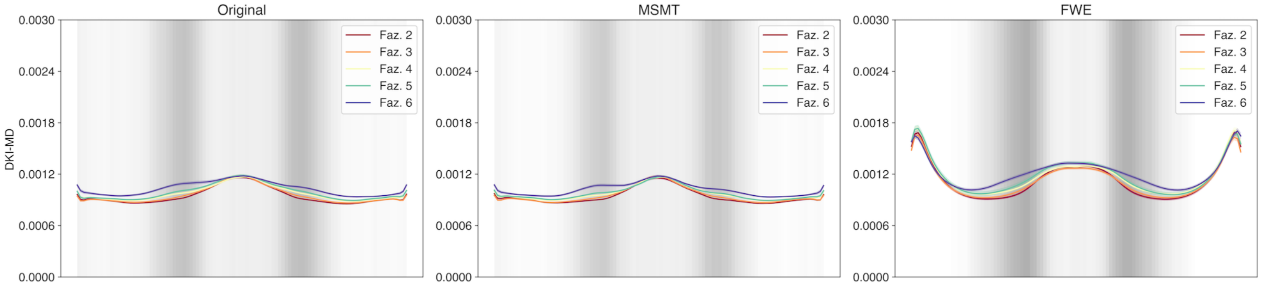

Supplemental Figure 102: Multi-shell Superior Frontal Callosum DKI-MD profiles.

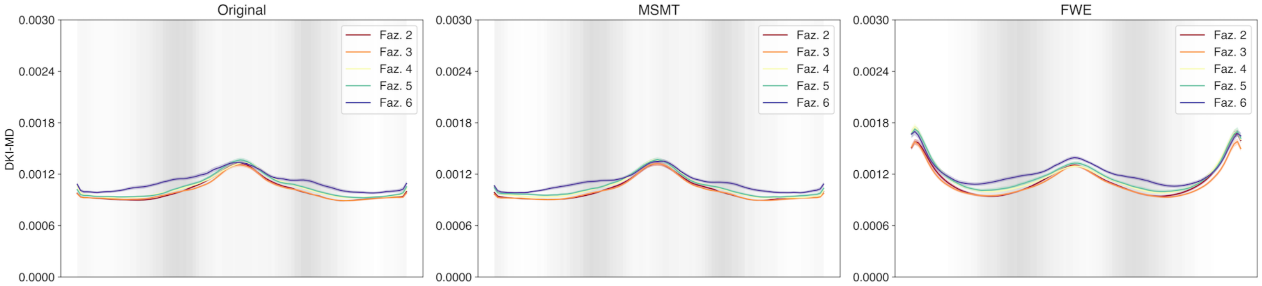

Supplemental Figure 103: Multi-shell Superior Parietal Corpus Callosum DKI-MD profiles.

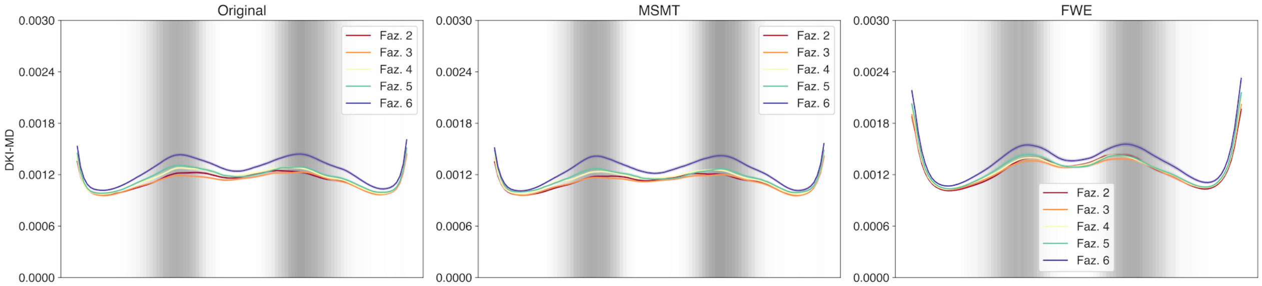

Supplemental Figure 104: Multi-shell Temporal Corpus Callosum DKI-MD profiles.

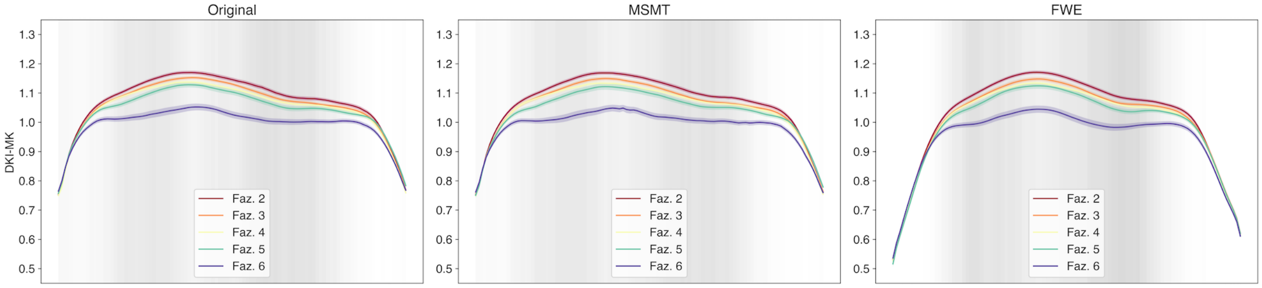

Supplemental Figure 105: Multi-shell Left Arcuate Fasiculus DKI-MK profiles.

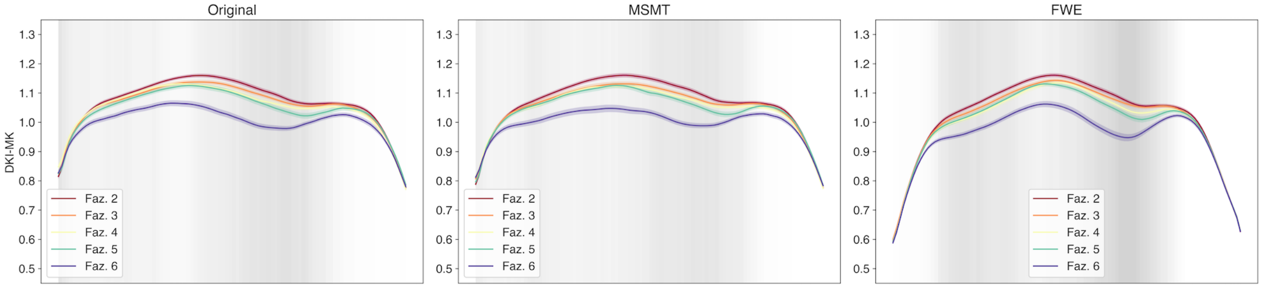

Supplemental Figure 106: Multi-shell Right Arcuate Fasiculus DKI-MK profiles.

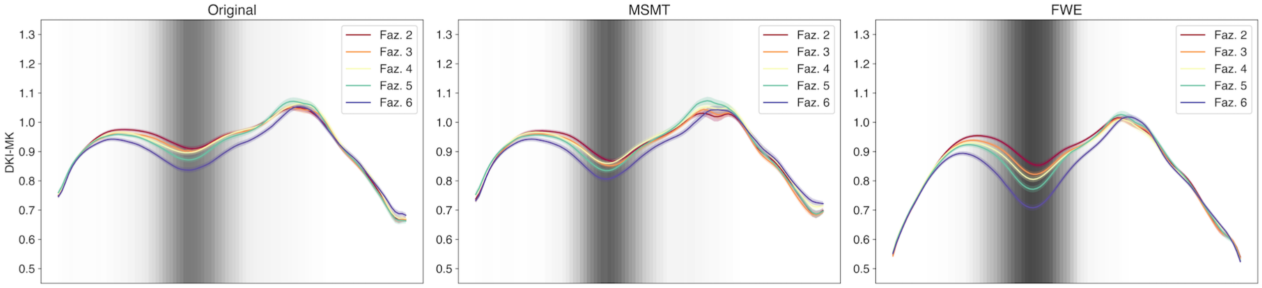

Supplemental Figure 107: Multi-shell Left Anterior Thalamic Radiation DKI-MK profiles.

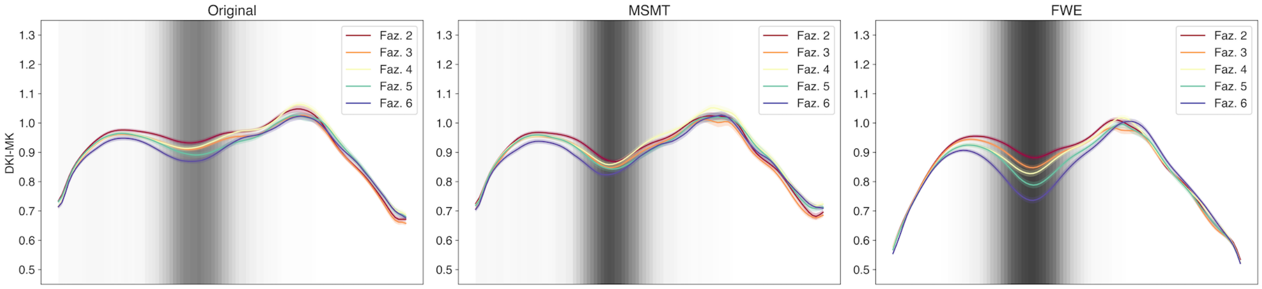

Supplemental Figure 108: Multi-shell Right Anterior Thalamic Radiation DKI-MK profiles.

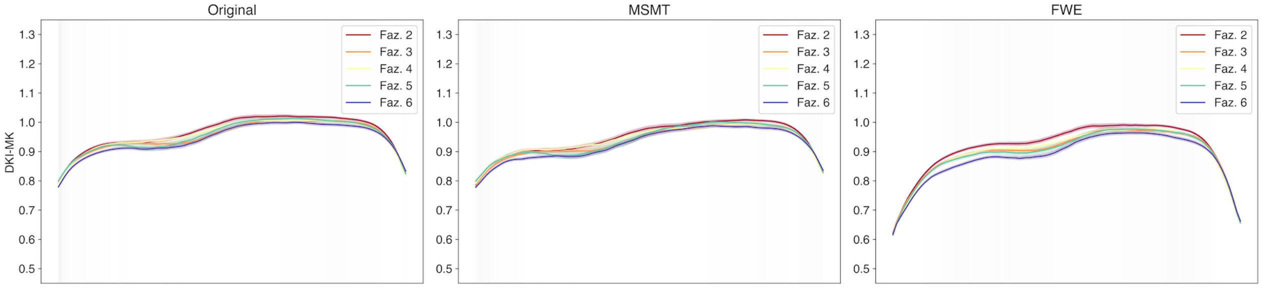

Supplemental Figure 109: Multi-shell Left Cingulum Cingulate DKI-MK profiles.

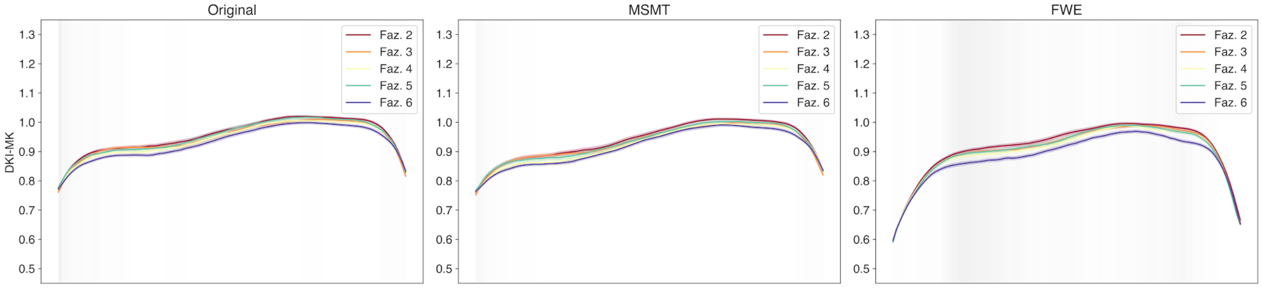

Supplemental Figure 110: Multi-shell Right Cingulum Cingulate DKI-MK profiles.

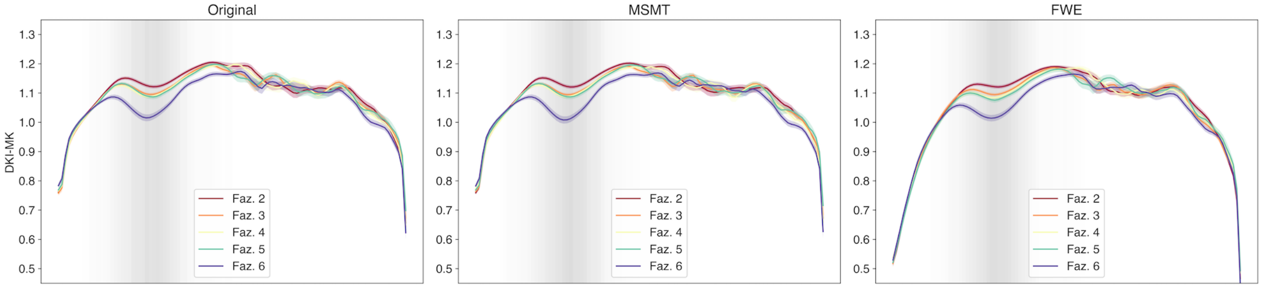

Supplemental Figure 111: Multi-shell Left Corticospinal Tract DKI-MK profiles.

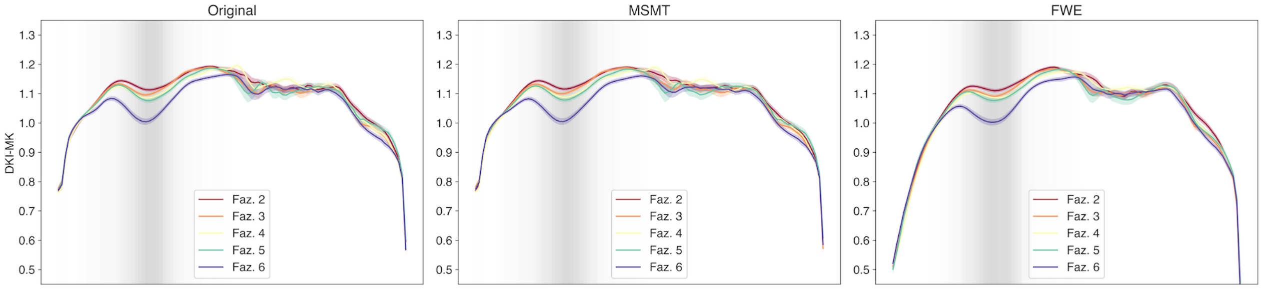

Supplemental Figure 112: Multi-shell Right Corticospinal Tract DKI-MK profiles.

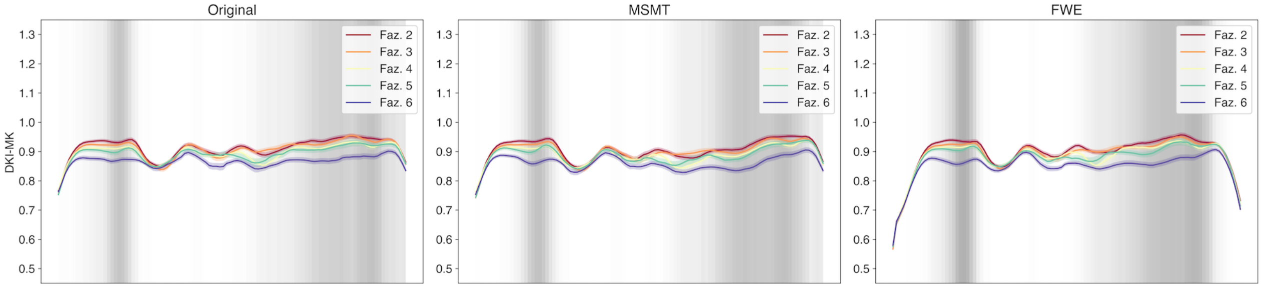

Supplemental Figure 113: Multi-shell Left Inferior Fronto-Occipital Fasiculus DKI-MK profiles.

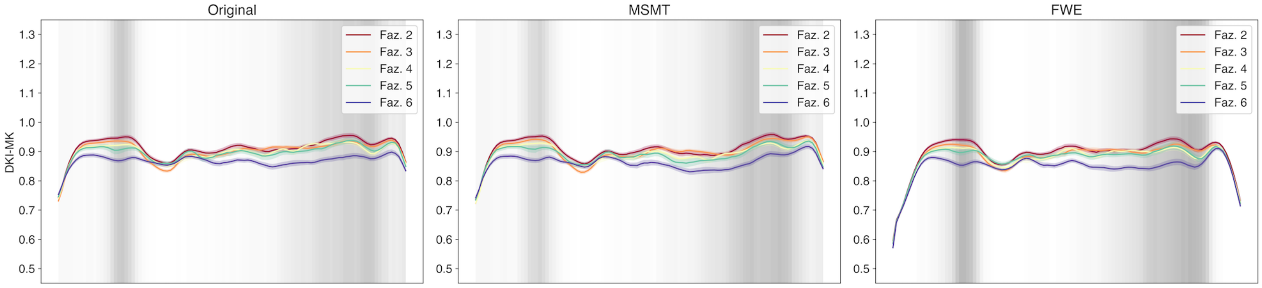

Supplemental Figure 114: Multi-shell Right Inferior Fronto-Occipital Fasiculus DKI-MK profiles.

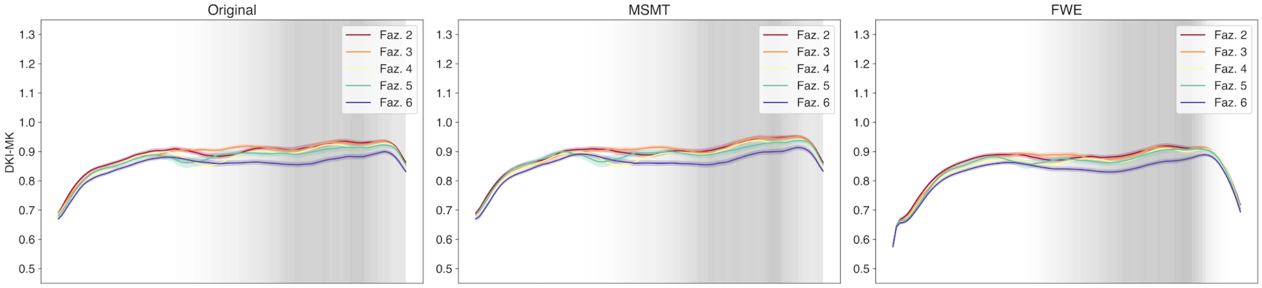

Supplemental Figure 115: Multi-shell Left Inferior Longitudinal Fasiculus DKI-MK profiles.

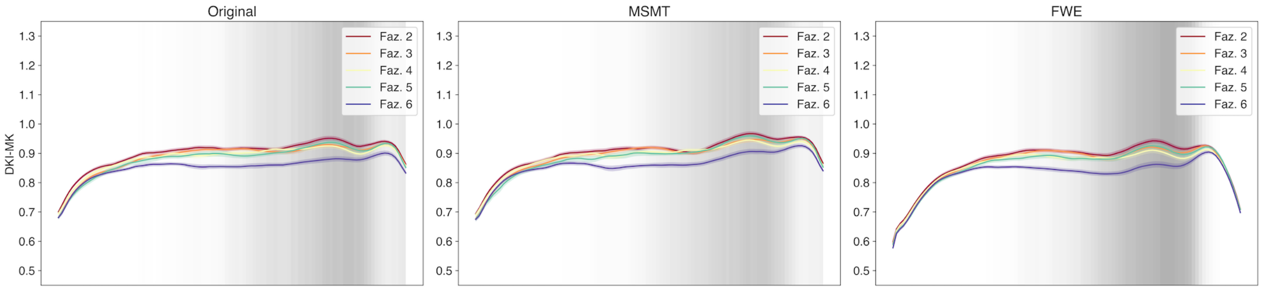

Supplemental Figure 116: Multi-shell Right Inferior Longitudinal Fasiculus DKI-MK profiles.

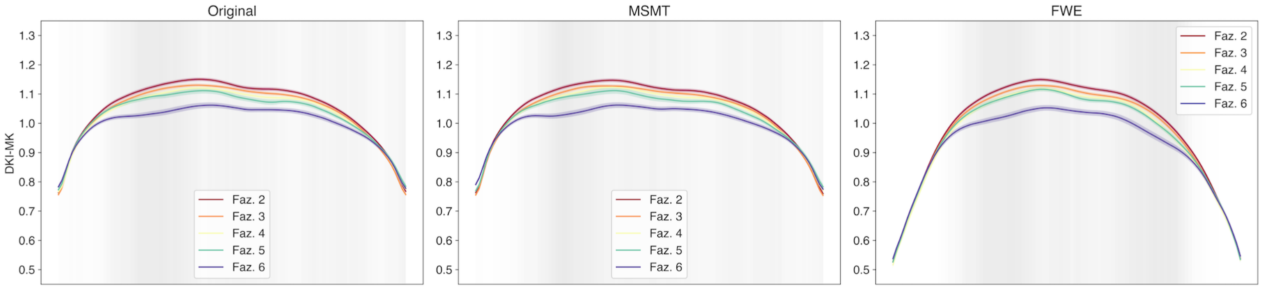

Supplemental Figure 117: Multi-shell Left Superior Longitudinal Fasiculus DKI-MK profiles.

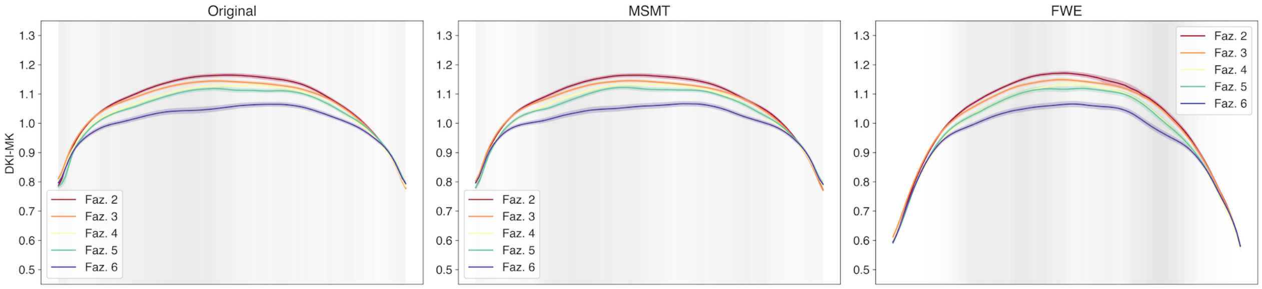

Supplemental Figure 118: Multi-shell Right Superior Longitudinal Fasiculus DKI-MK profiles.

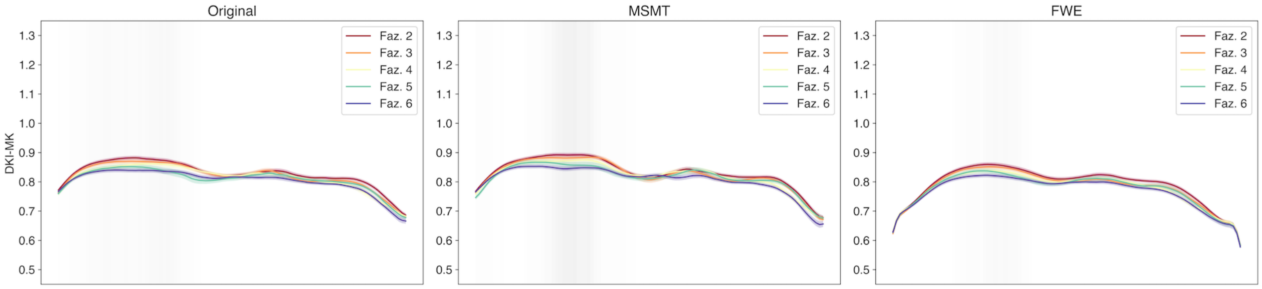

Supplemental Figure 119: Multi-shell Left Uncinate Fasiculus DKI-MK profiles.

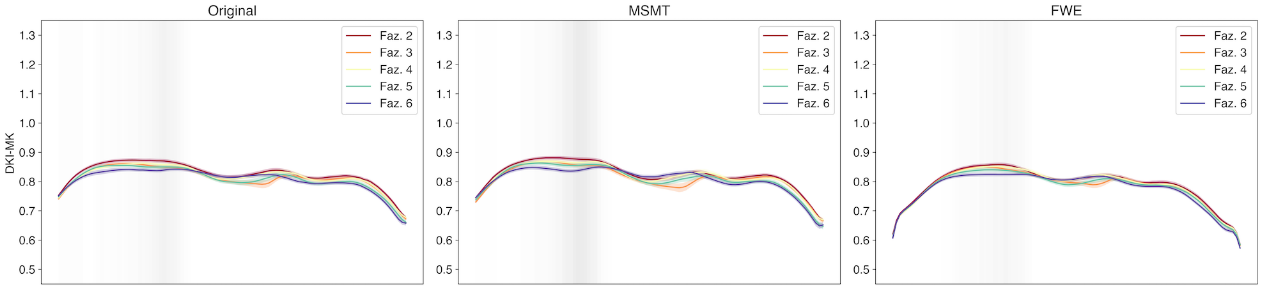

Supplemental Figure 120: Multi-shell Right Uncinate Fasiculus DKI-MK profiles.

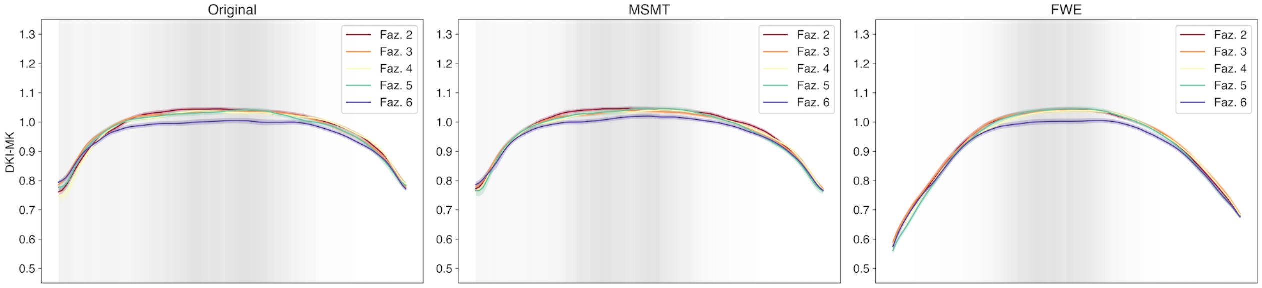

Supplemental Figure 121: Multi-shell Left Vertical Occipital Fasiculus DKI-MK profiles.

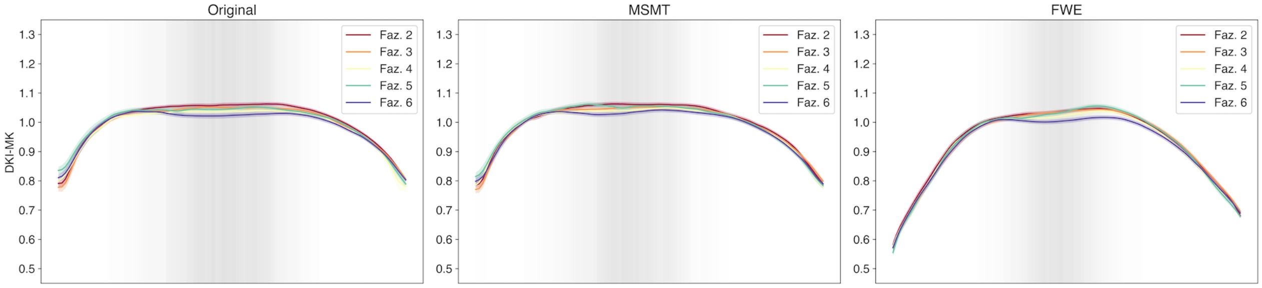

Supplemental Figure 122: Multi-shell Right Vertical Occipital Fasiculus DKI-MK profiles.

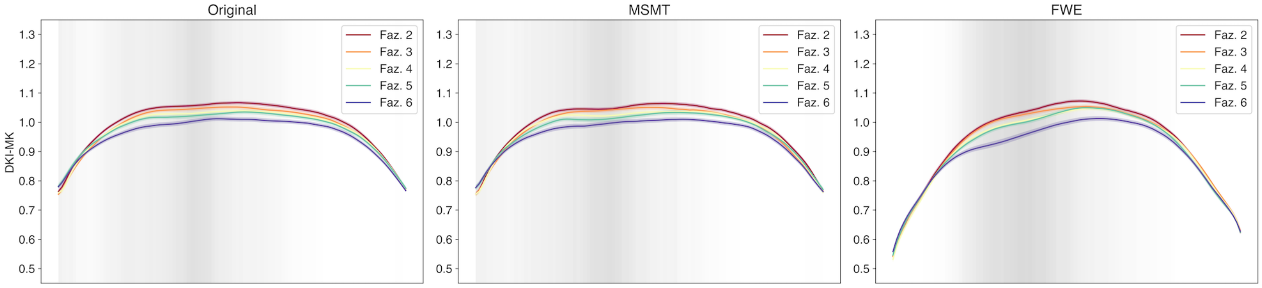

Supplemental Figure 123: Multi-shell Left Posterior Arcuate Fasiculus DKI-MK profiles.

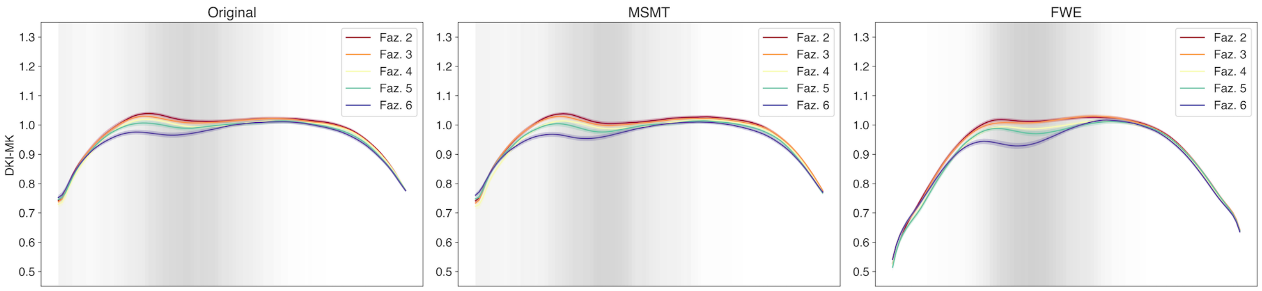

Supplemental Figure 124: Multi-shell Right Posterior Arcuate Fasiculus DKI-MK profiles.

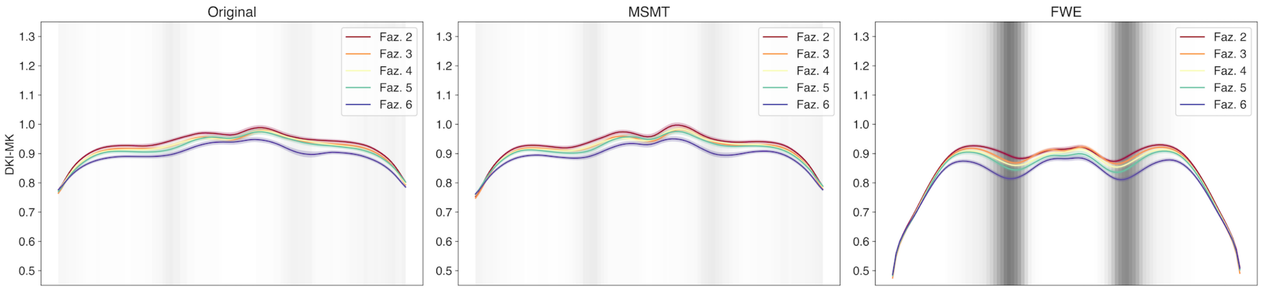

Supplemental Figure 125: Multi-shell Anterior Frontal Callosum DKI-MK profiles.

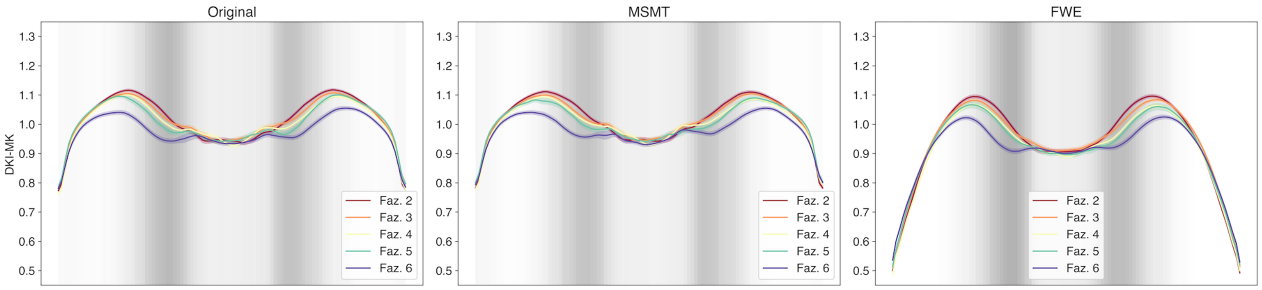

Supplemental Figure 126: Multi-shell Motor Corpus Callosum DKI-MK profiles.

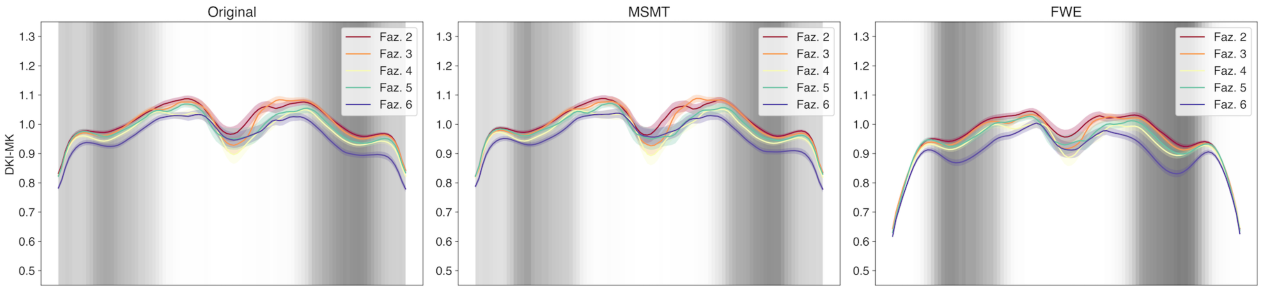

Supplemental Figure 127: Multi-shell Occipital Corpus Callosum DKI-MK profiles.

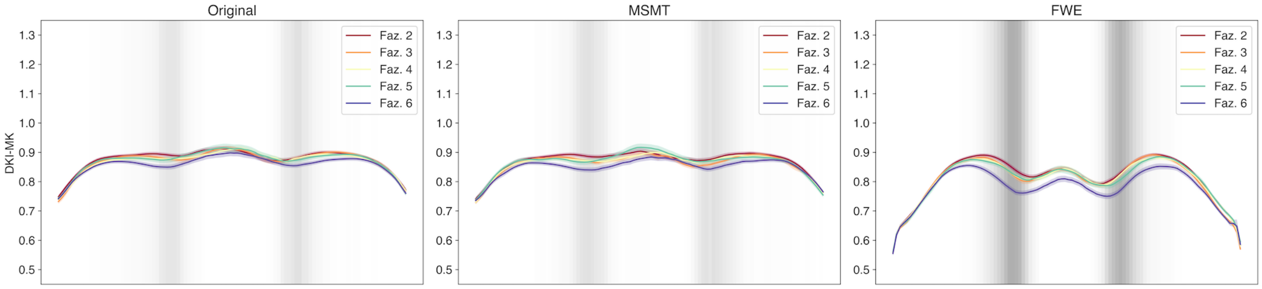

Supplemental Figure 128: Multi-shell Orbital Corpus Callosum DKI-MK profiles.

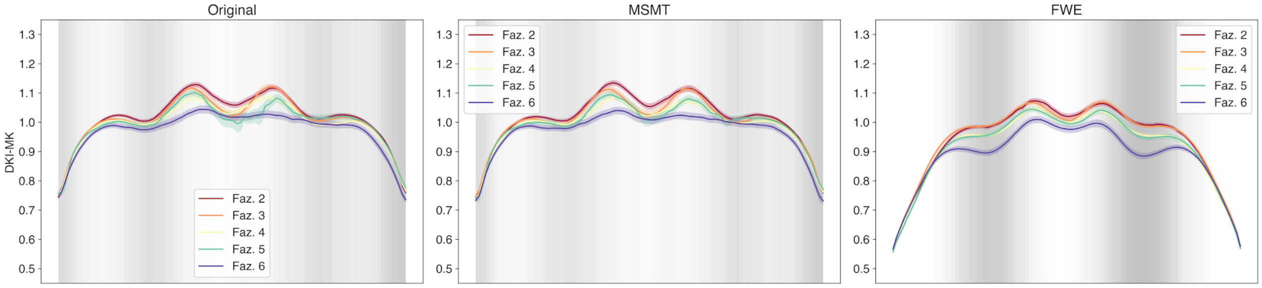

Supplemental Figure 129: Multi-shell Posterior Parietal Corpus Callosum DKI-MK profiles.

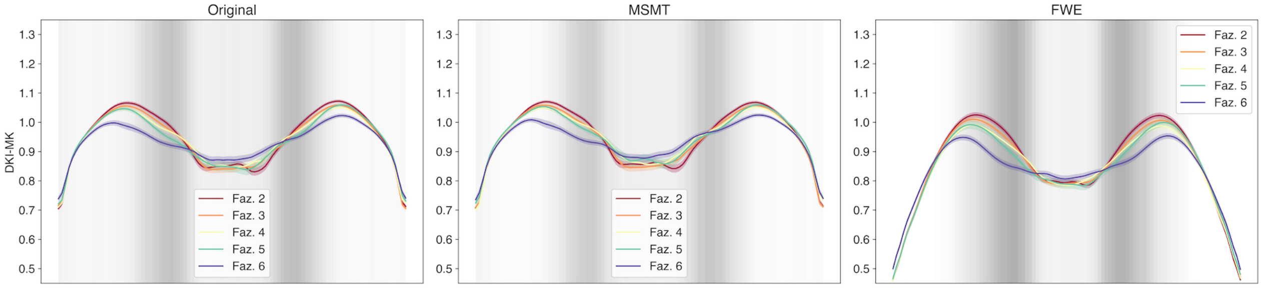

Supplemental Figure 130: Multi-shell Superior Frontal Callosum DKI-MK profiles.

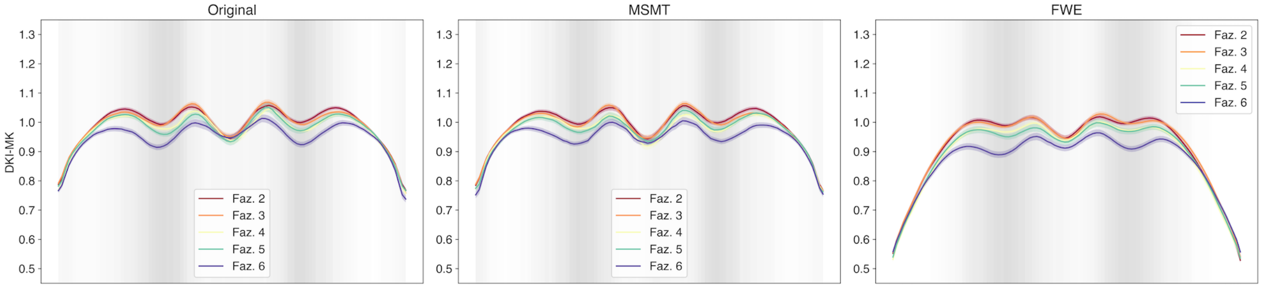

Supplemental Figure 131: Multi-shell Superior Parietal Corpus Callosum DKI-MK profiles.

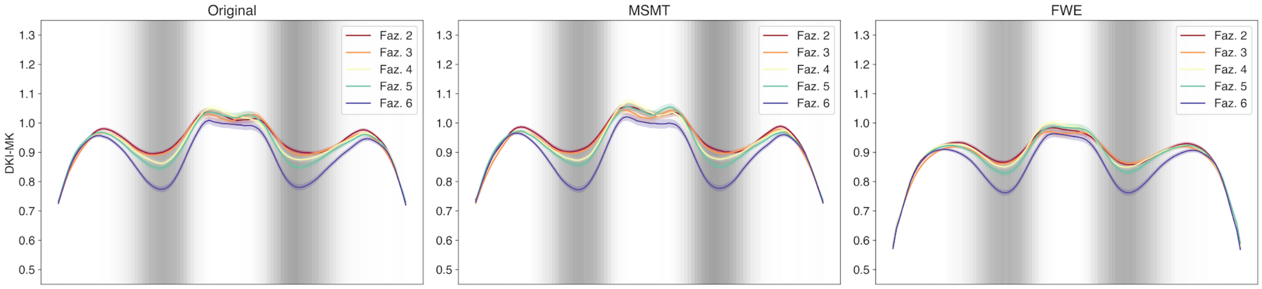

Supplemental Figure 132: Multi-shell Temporal Corpus Callosum DKI-MK profiles.

1.6.2 Single-shell tract profiles

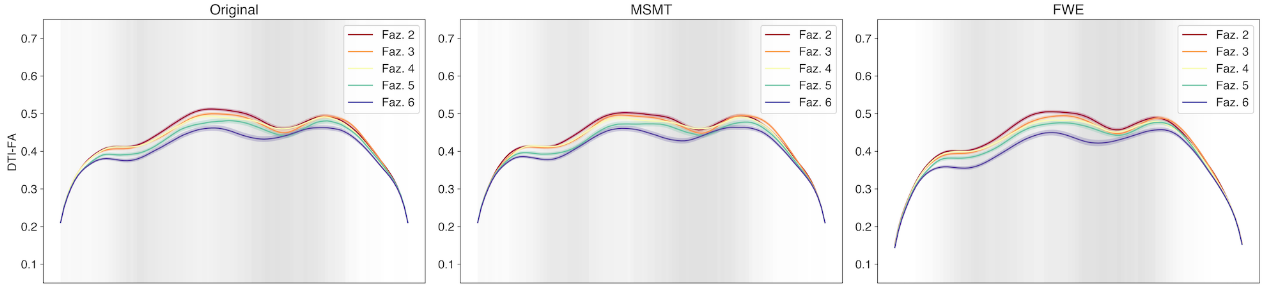

Supplemental Figure 133: Single-shell Left Arcuate Fasiculus DTI-FA profiles.

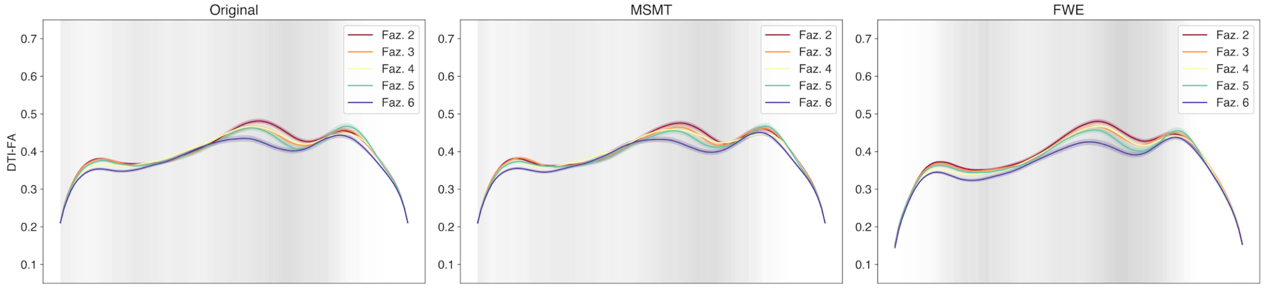

Supplemental Figure 134: Single-shell Right Arcuate Fasiculus DTI-FA profiles.

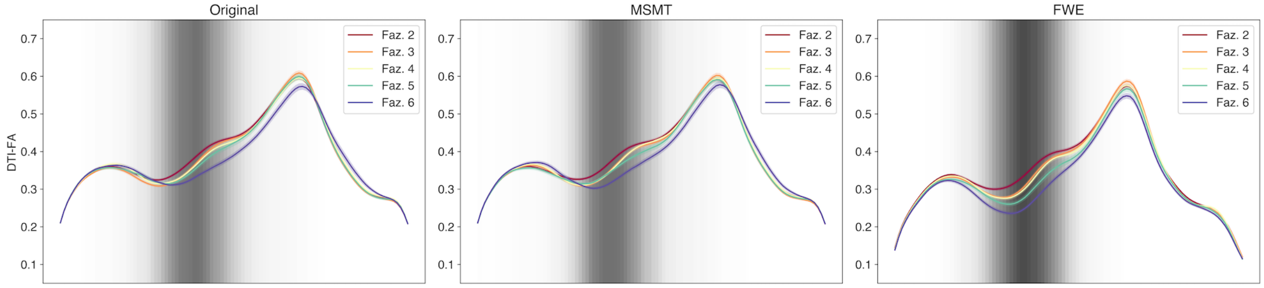

Supplemental Figure 135: Single-shell Left Anterior Thalamic Radiation DTI-FA profiles.

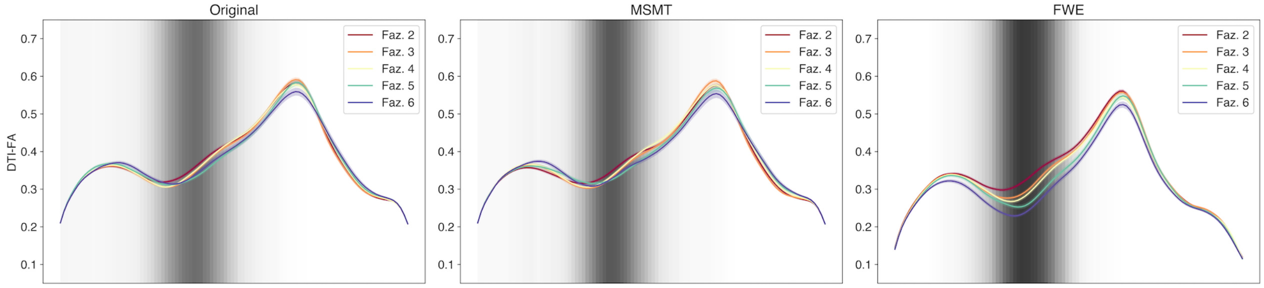

Supplemental Figure 136: Single-shell Right Anterior Thalamic Radiation DTI-FA profiles.

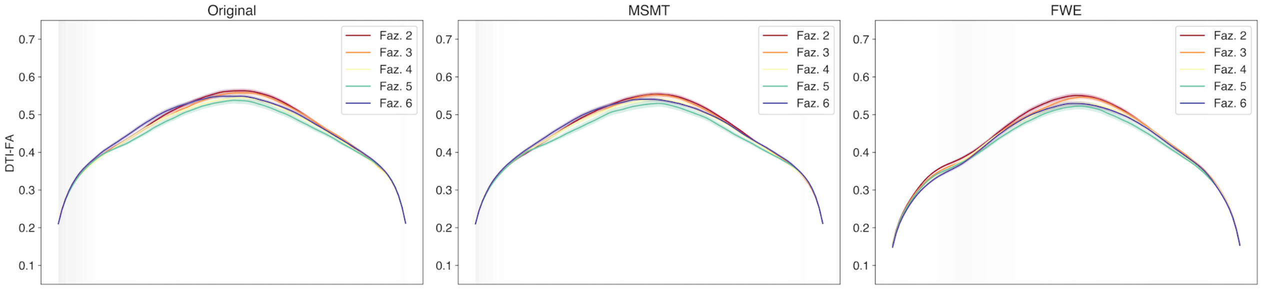

Supplemental Figure 137: Single-shell Left Cingulum Cingulate DTI-FA profiles.

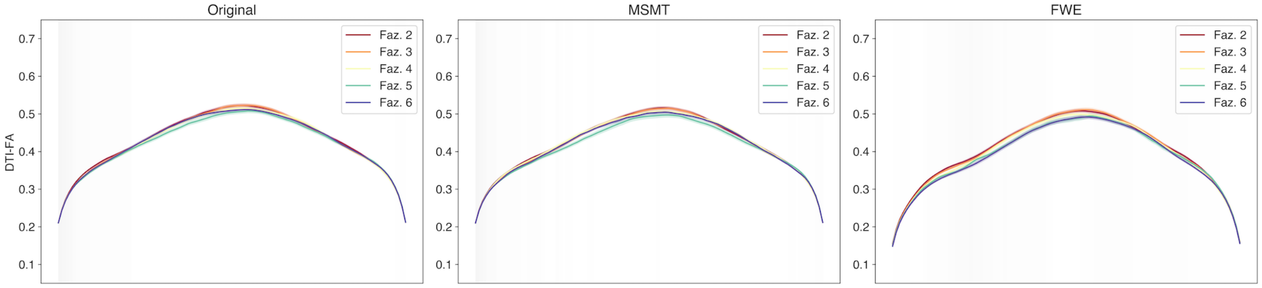

Supplemental Figure 138: Single-shell Right Cingulum Cingulate DTI-FA profiles.

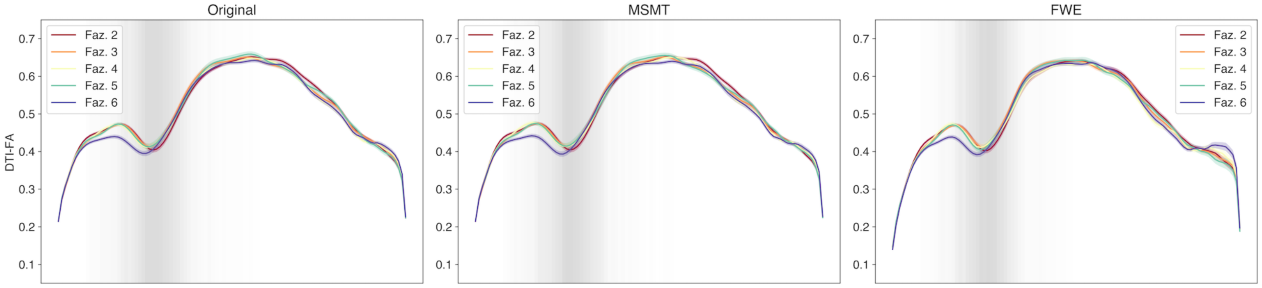

Supplemental Figure 139: Single-shell Left Corticospinal Tract DTI-FA profiles.

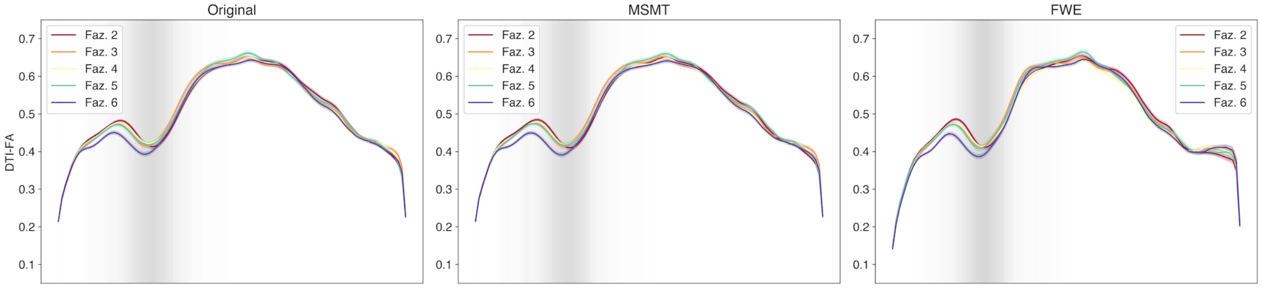

Supplemental Figure 140: Single-shell Right Corticospinal Tract DTI-FA profiles.

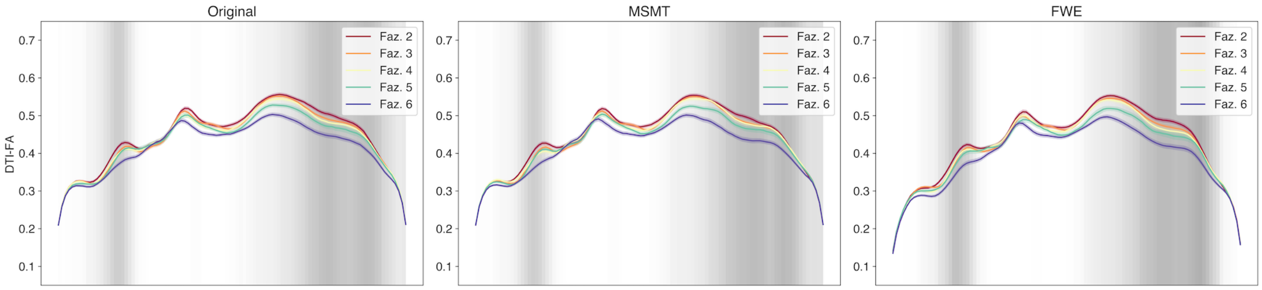

Supplemental Figure 141: Single-shell Left Inferior Fronto-Occipital Fasiculus DTI-FA profiles.

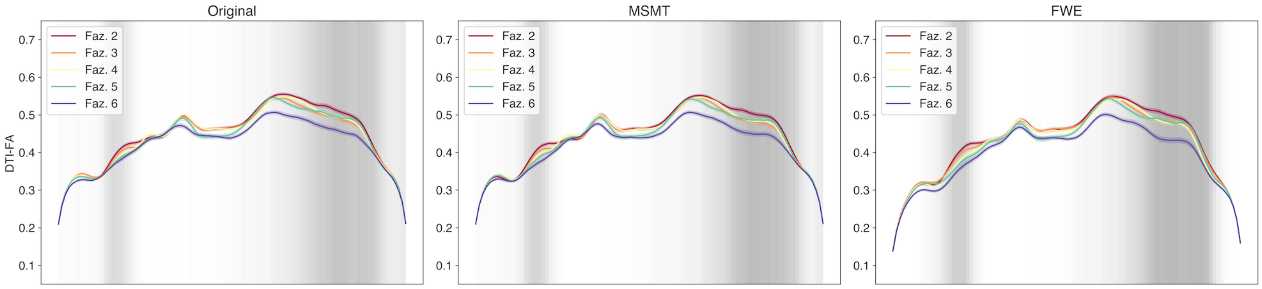

Supplemental Figure 142: Single-shell Right Inferior Fronto-Occipital Fasiculus DTI-FA profiles.

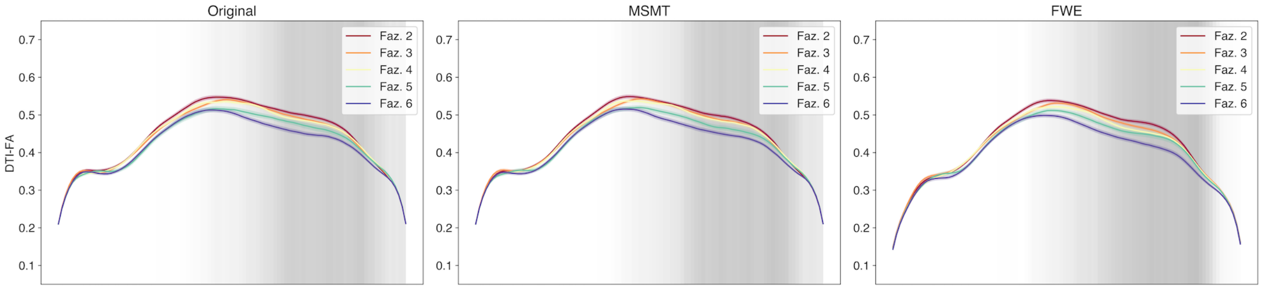

Supplemental Figure 143: Single-shell Left Inferior Longitudinal Fasiculus DTI-FA profiles.

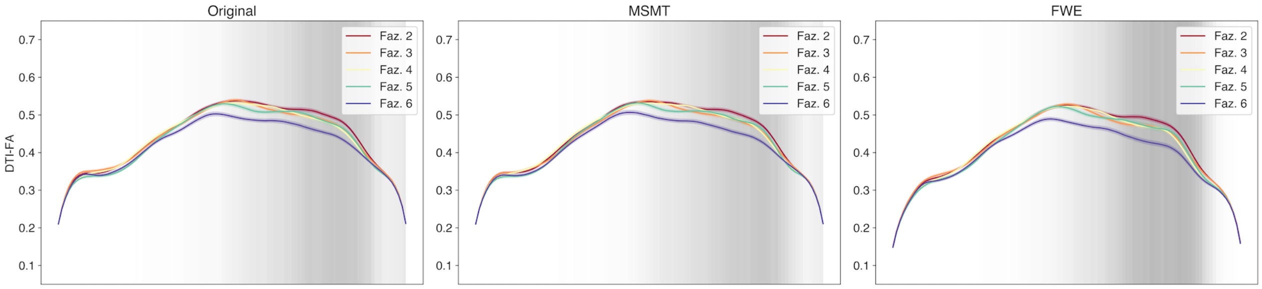

Supplemental Figure 144: Single-shell Right Inferior Longitudinal Fasiculus DTI-FA profiles.

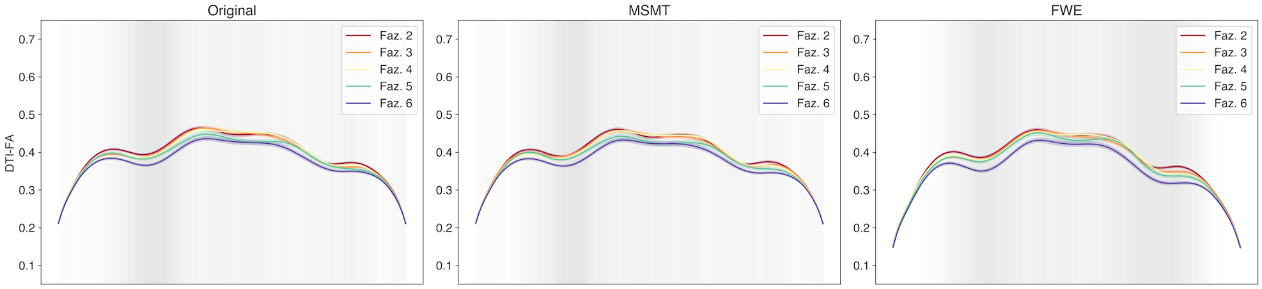

Supplemental Figure 145: Single-shell Left Superior Longitudinal Fasiculus DTI-FA profiles.

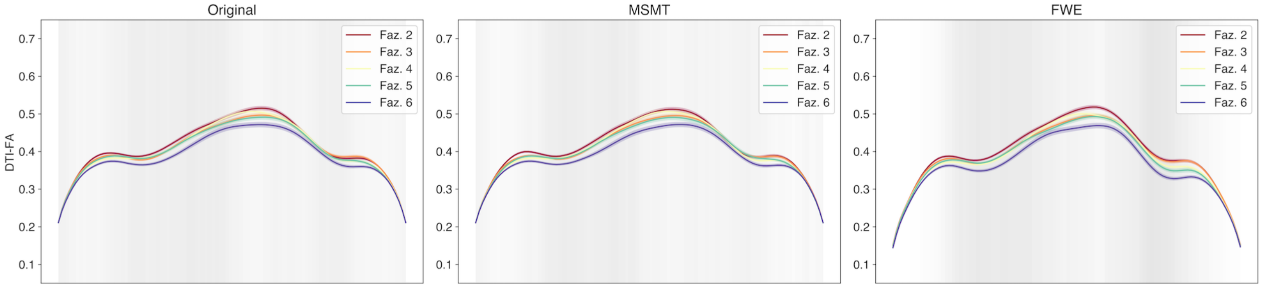

Supplemental Figure 146: Single-shell Right Superior Longitudinal Fasiculus DTI-FA profiles.

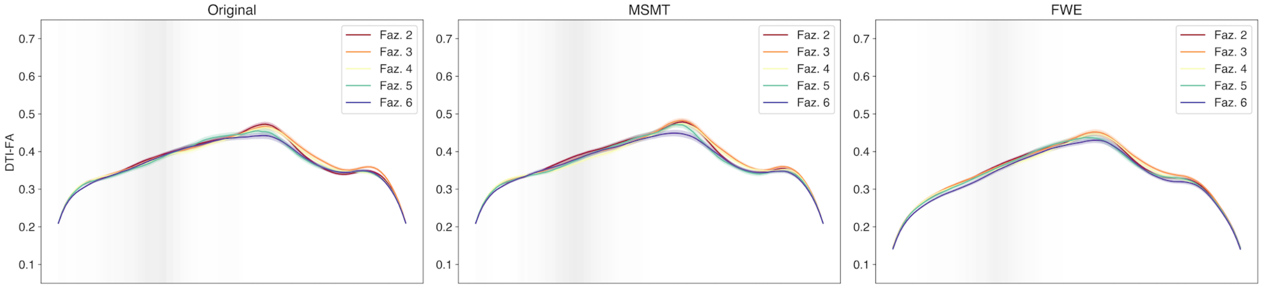

Supplemental Figure 147: Single-shell Left Uncinate Fasiculus DTI-FA profiles.

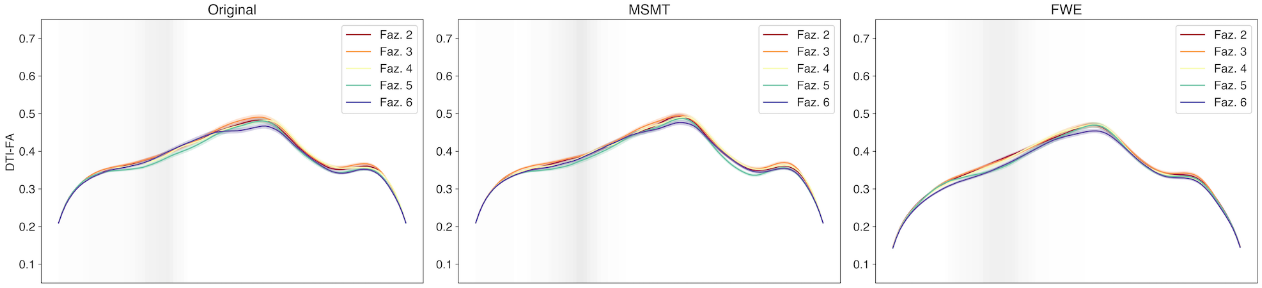

Supplemental Figure 148: Single-shell Right Uncinate Fasiculus DTI-FA profiles.

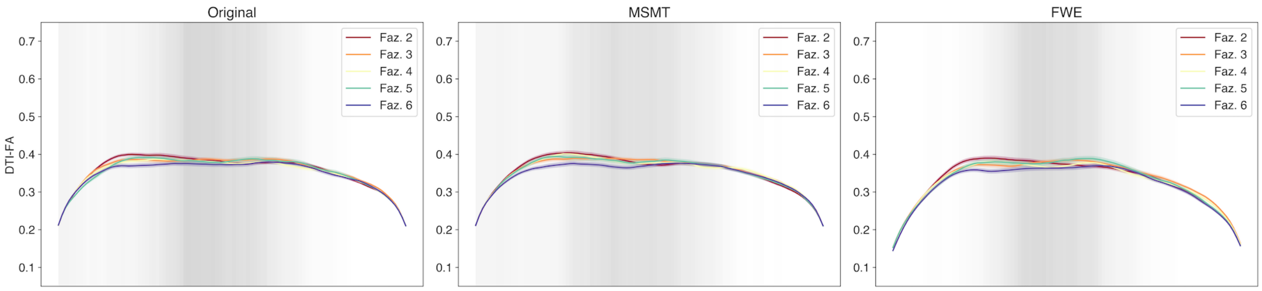

Supplemental Figure 149: Single-shell Left Vertical Occipital Fasiculus DTI-FA profiles.

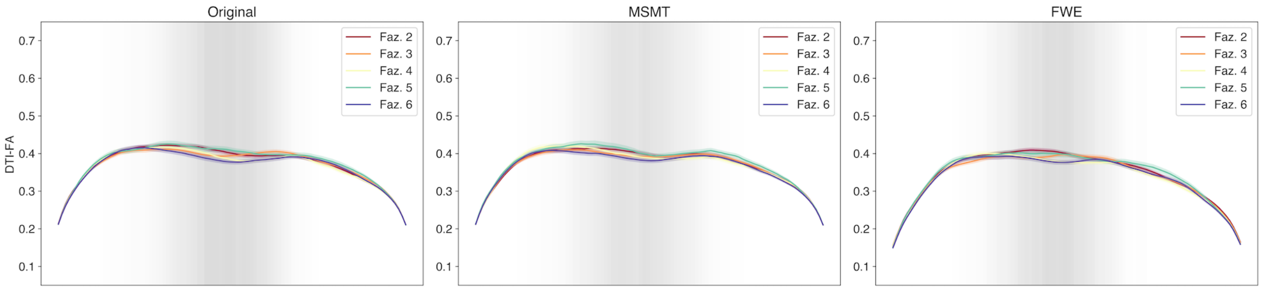

Supplemental Figure 150: Single-shell Right Vertical Occipital Fasiculus DTI-FA profiles.

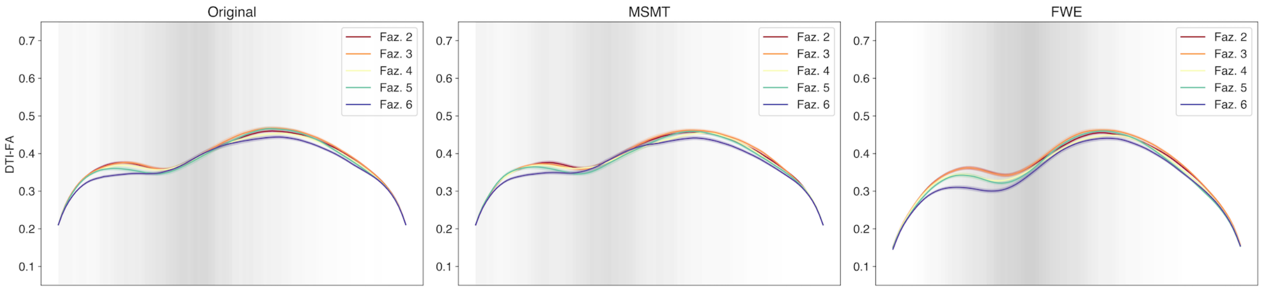

Supplemental Figure 151: Single-shell Left Posterior Arcuate Fasiculus DTI-FA profiles.

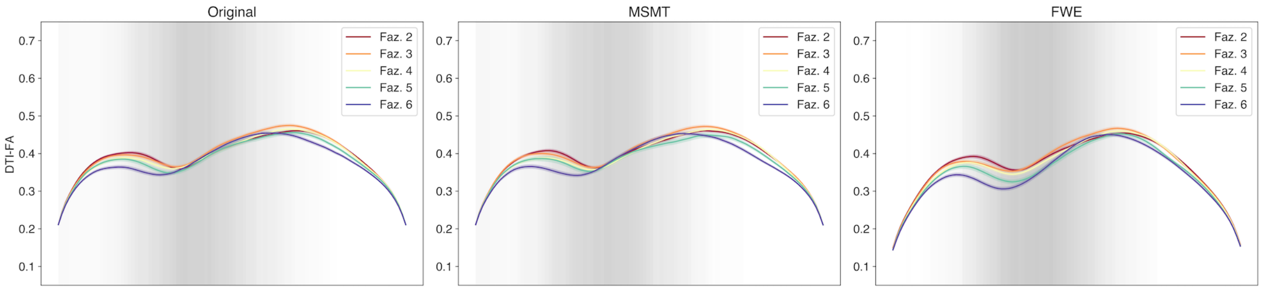

Supplemental Figure 152: Single-shell Right Posterior Arcuate Fasiculus DTI-FA profiles.

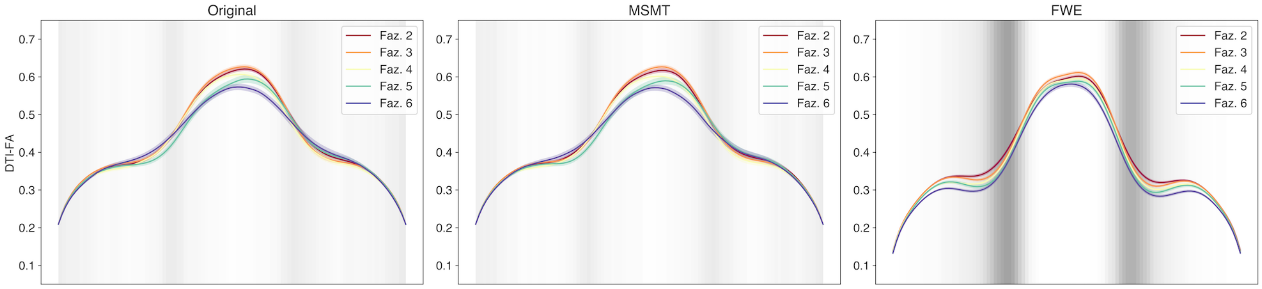

Supplemental Figure 153: Single-shell Anterior Frontal Callosum DTI-FA profiles.

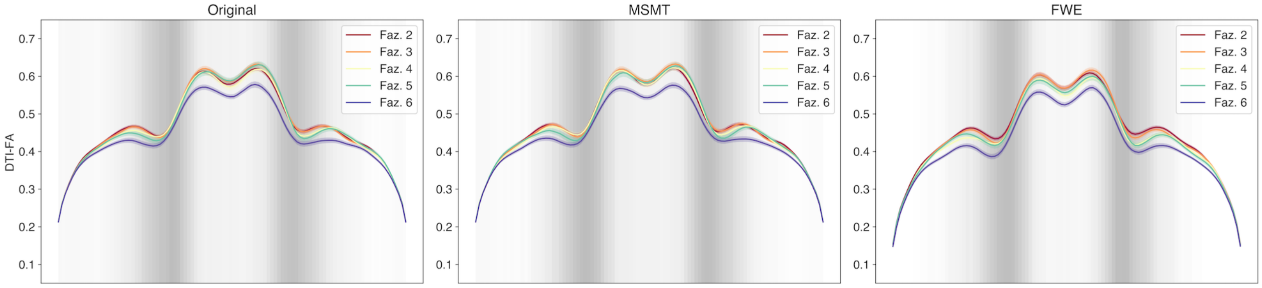

Supplemental Figure 154: Single-shell Motor Corpus Callosum DTI-FA profiles.

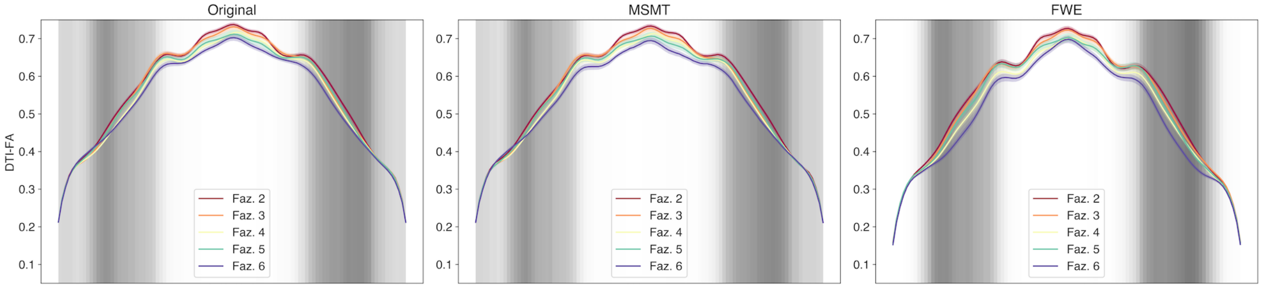

Supplemental Figure 155: Single-shell Occipital Corpus Callosum DTI-FA profiles.

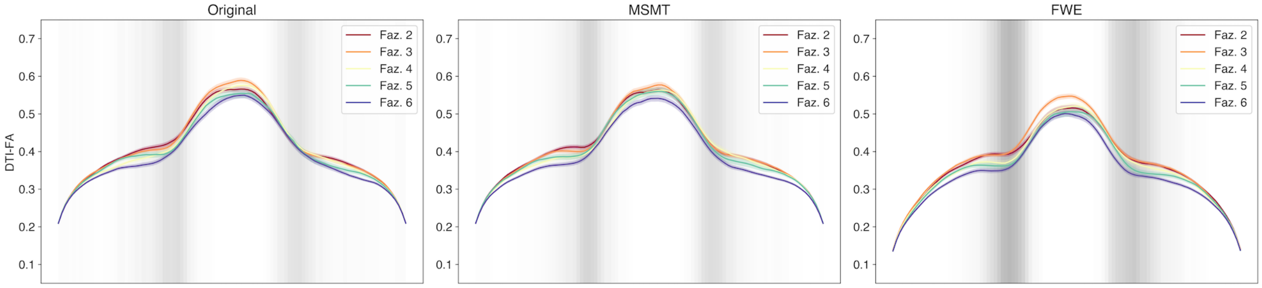

Supplemental Figure 156: Single-shell Orbital Corpus Callosum DTI-FA profiles.

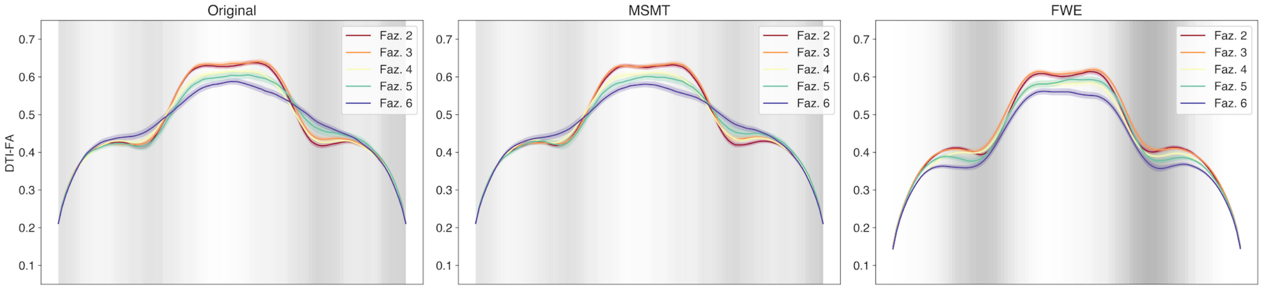

Supplemental Figure 157: Single-shell Posterior Parietal Corpus Callosum DTI-FA profiles.

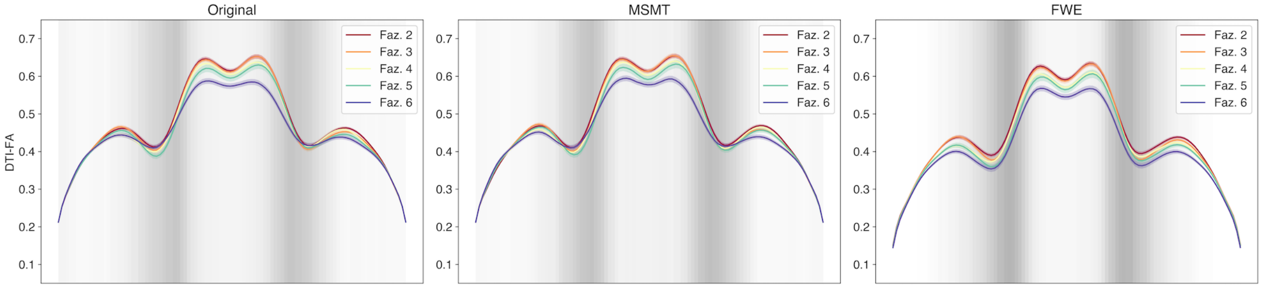

Supplemental Figure 158: Single-shell Superior Frontal Callosum DTI-FA profiles.

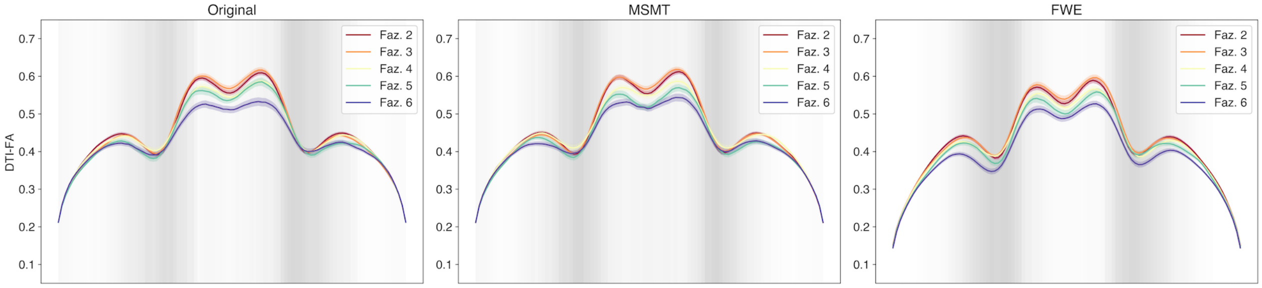

Supplemental Figure 159: Single-shell Superior Parietal Corpus Callosum DTI-FA profiles.

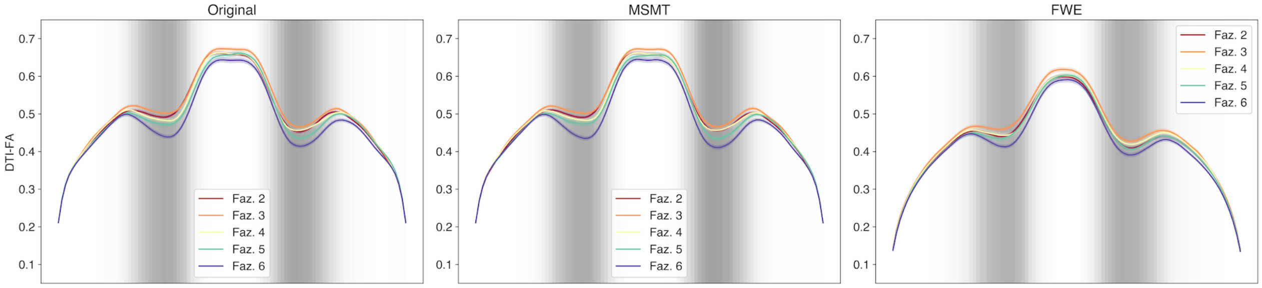

Supplemental Figure 160: Single-shell Temporal Corpus Callosum DTI-FA profiles.

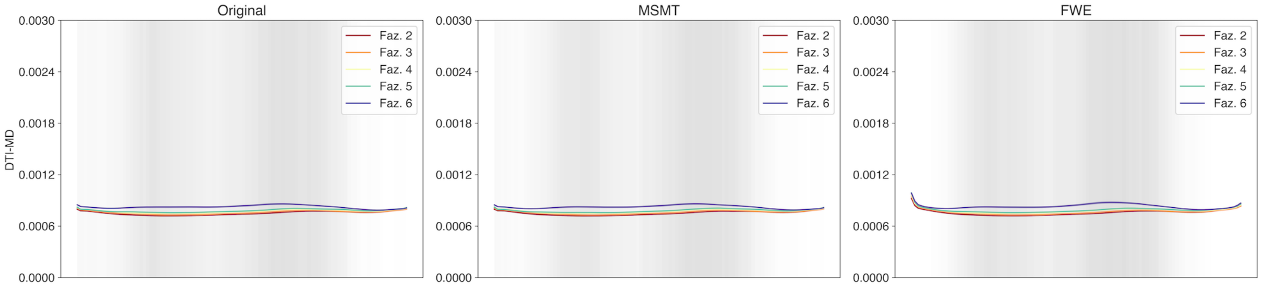

Supplemental Figure 161: Single-shell Left Arcuate Fasiculus DTI-MD profiles.

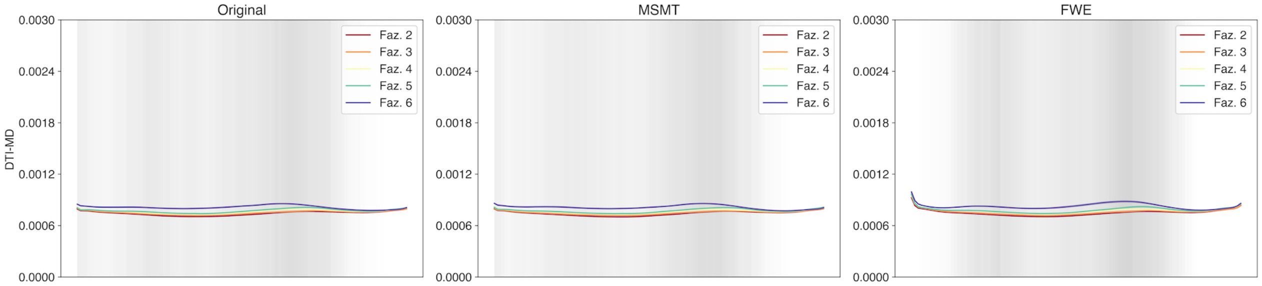

Supplemental Figure 162: Single-shell Right Arcuate Fasiculus DTI-MD profiles.

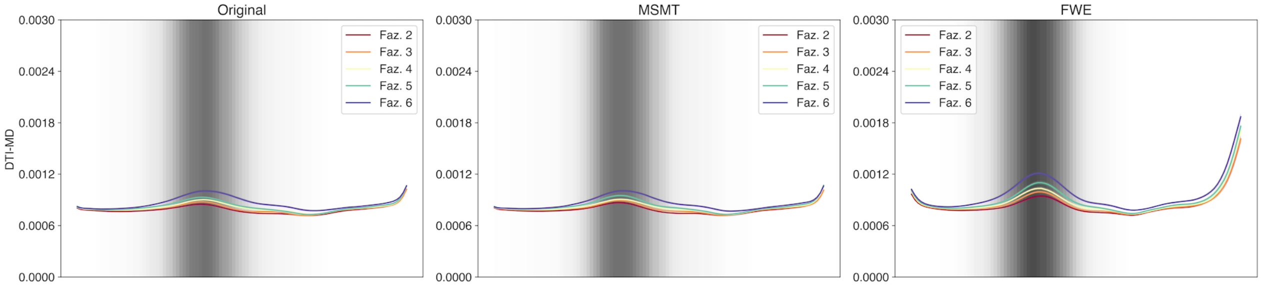

Supplemental Figure 163: Single-shell Left Anterior Thalamic Radiation DTI-MD profiles.

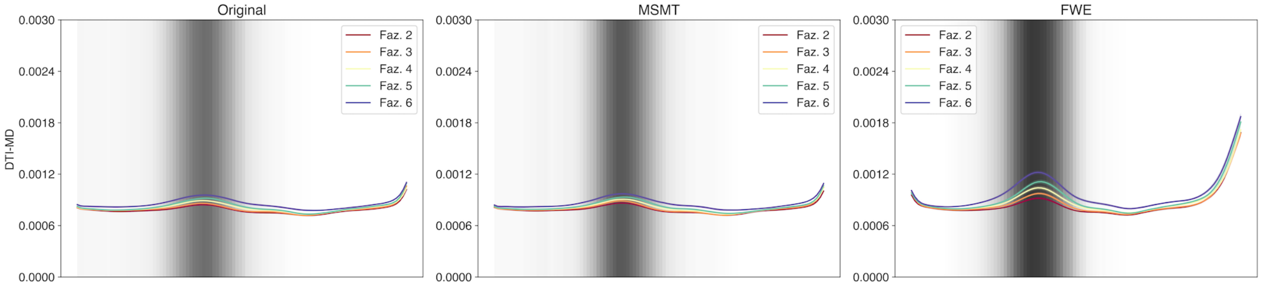

Supplemental Figure 164: Single-shell Right Anterior Thalamic Radiation DTI-MD profiles.

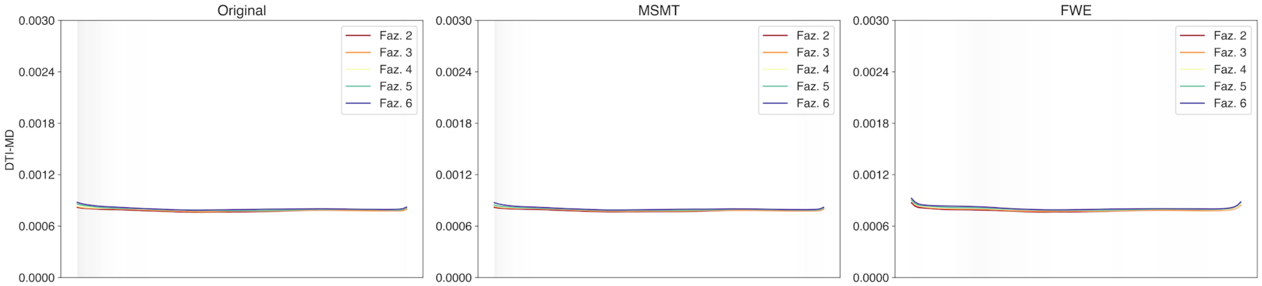

Supplemental Figure 165: Single-shell Left Cingulum Cingulate DTI-MD profiles.

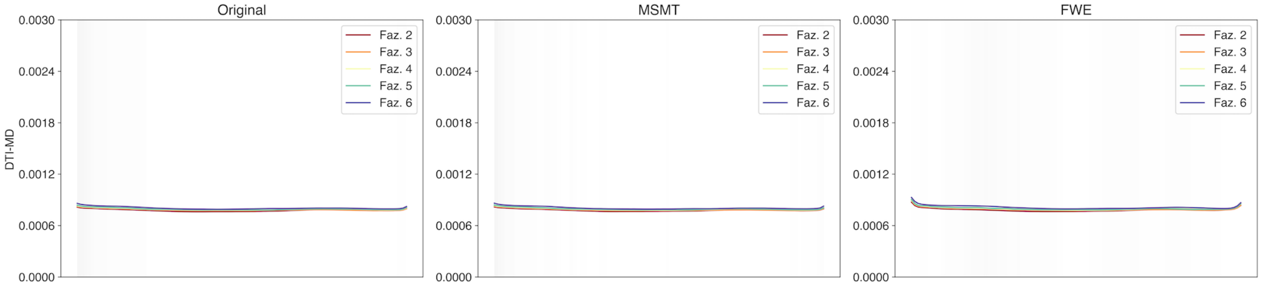

Supplemental Figure 166: Single-shell Right Cingulum Cingulate DTI-MD profiles.

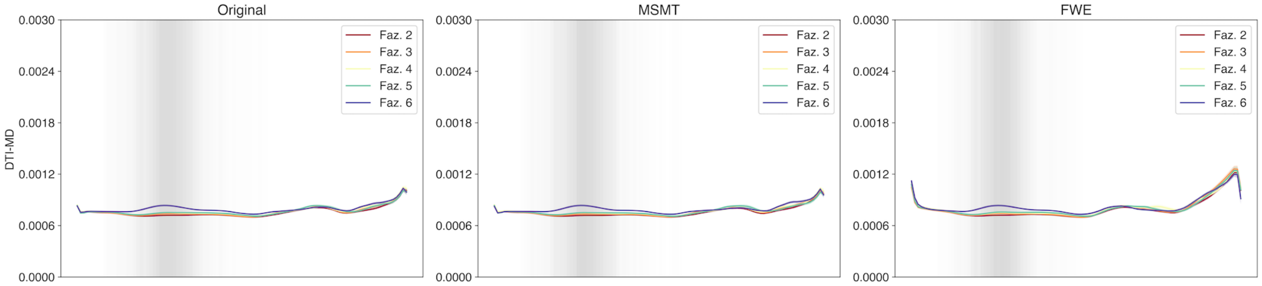

Supplemental Figure 167: Single-shell Left Corticospinal Tract DTI-MD profiles.

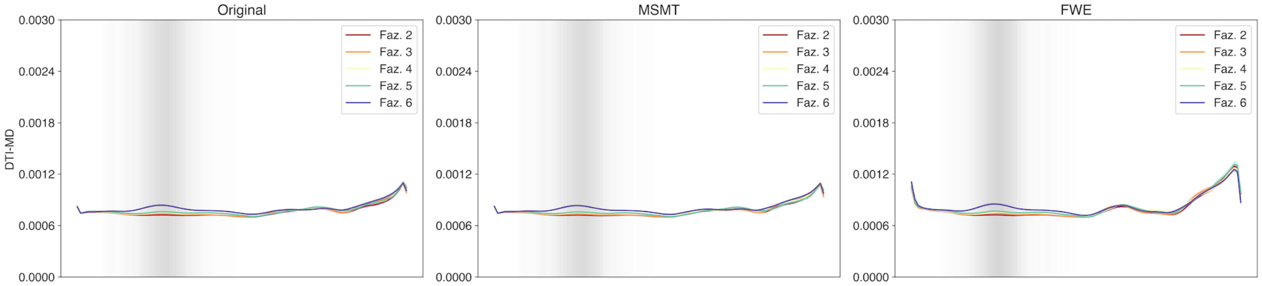

Supplemental Figure 168: Single-shell Right Corticospinal Tract DTI-MD profiles.

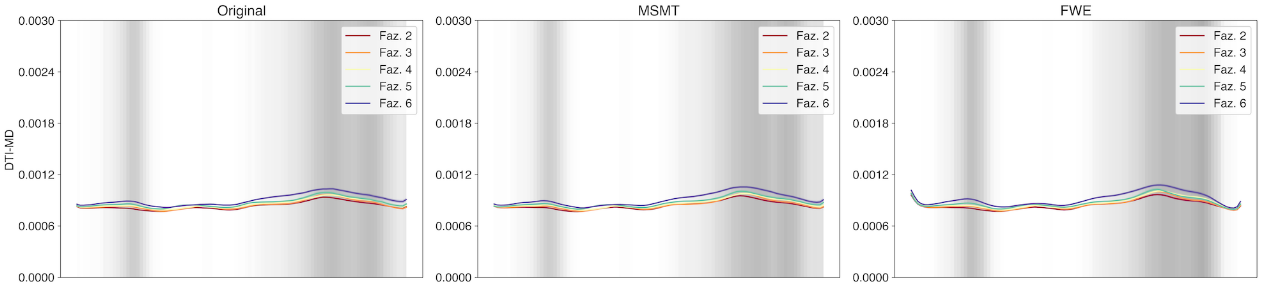

Supplemental Figure 169: Single-shell Left Inferior Fronto-Occipital Fasiculus DTI-MD profiles.

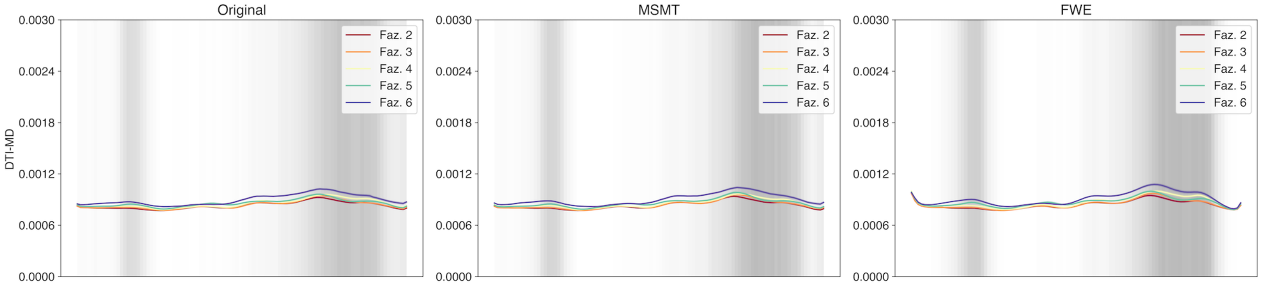

Supplemental Figure 170: Single-shell Right Inferior Fronto-Occipital Fasiculus DTI-MD profiles.

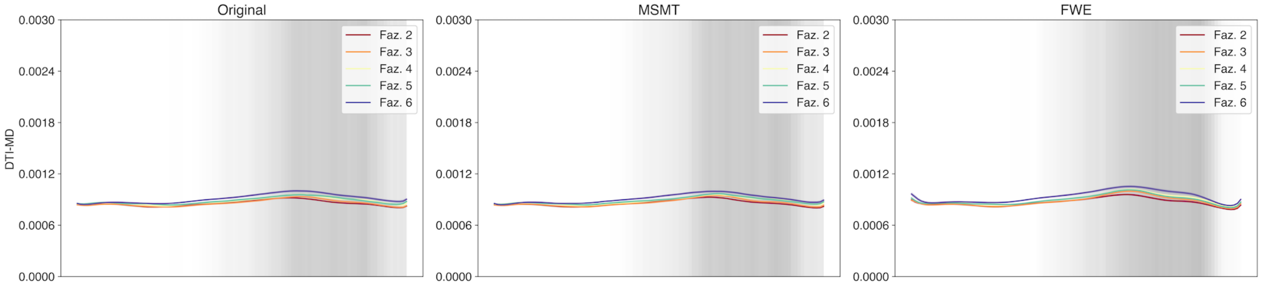

Supplemental Figure 171: Single-shell Left Inferior Longitudinal Fasiculus DTI-MD profiles.

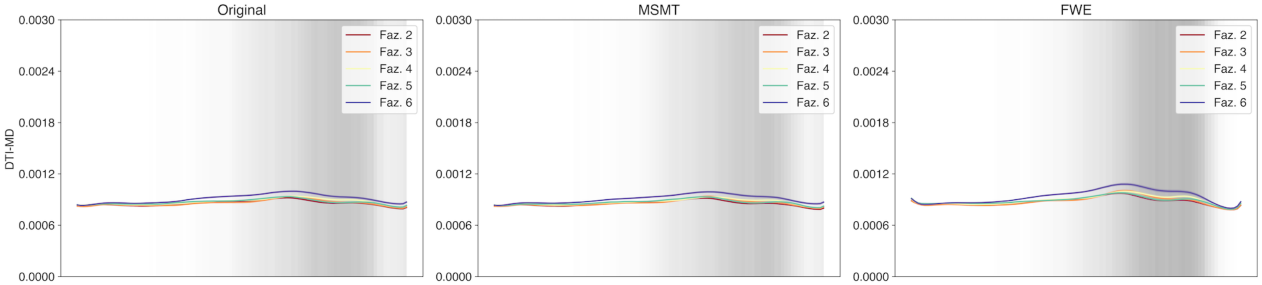

Supplemental Figure 172: Single-shell Right Inferior Longitudinal Fasiculus DTI-MD profiles.

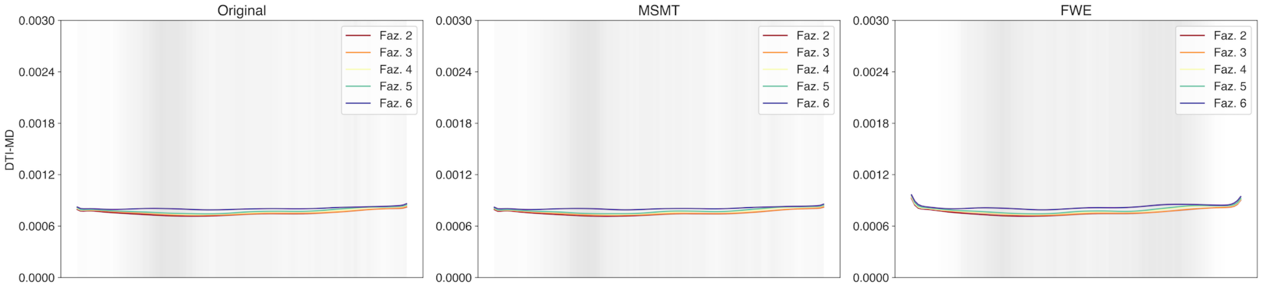

Supplemental Figure 173: Single-shell Left Superior Longitudinal Fasiculus DTI-MD profiles.

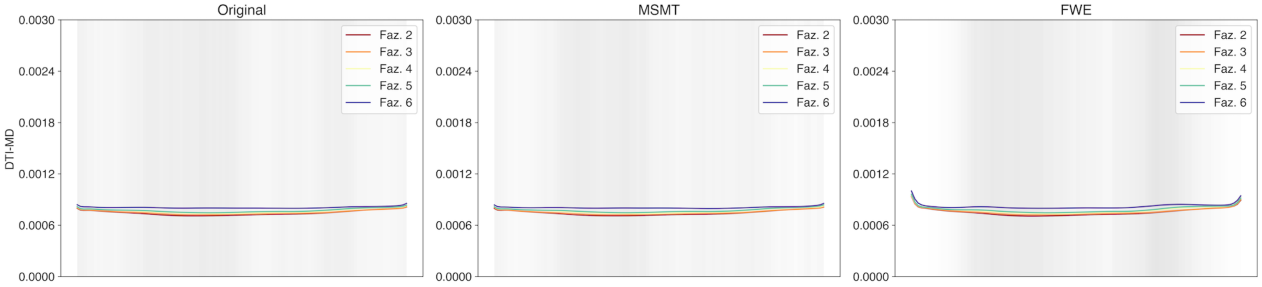

Supplemental Figure 174: Single-shell Right Superior Longitudinal Fasiculus DTI-MD profiles.

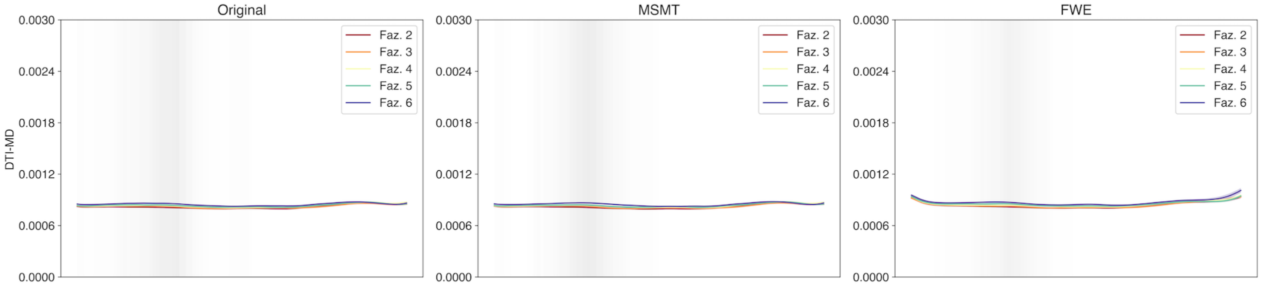

Supplemental Figure 175: Single-shell Left Uncinate Fasiculus DTI-MD profiles.

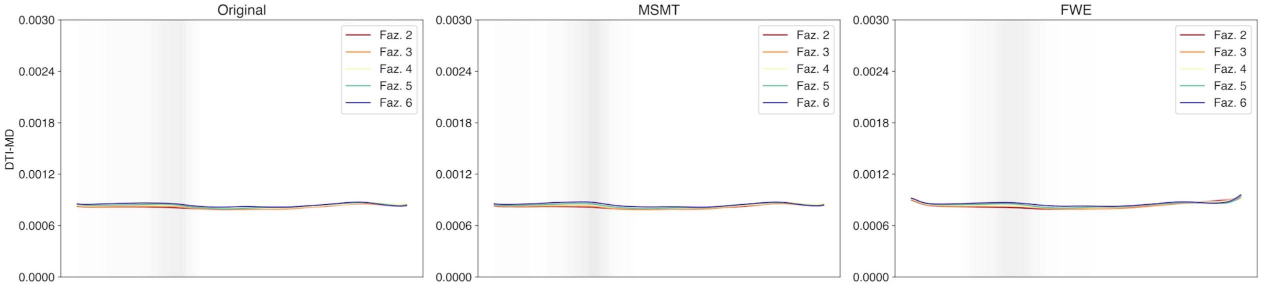

Supplemental Figure 176: Single-shell Right Uncinate Fasiculus DTI-MD profiles.

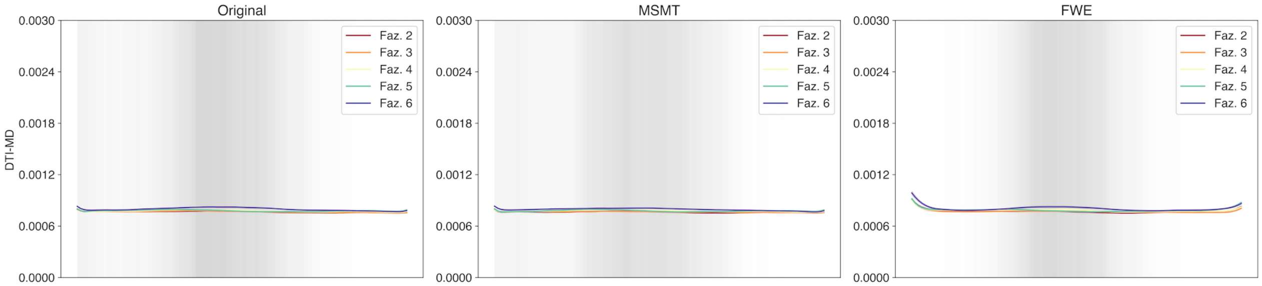

Supplemental Figure 177: Single-shell Left Vertical Occipital Fasiculus DTI-MD profiles.

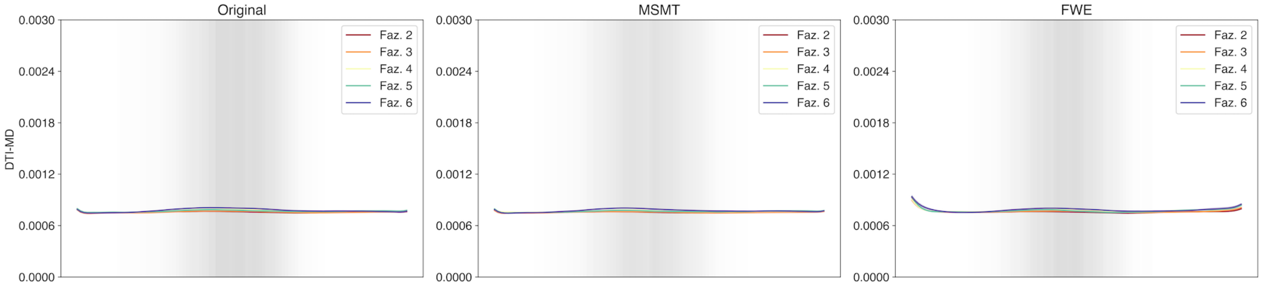

Supplemental Figure 178: Single-shell Right Vertical Occipital Fasiculus DTI-MD profiles.

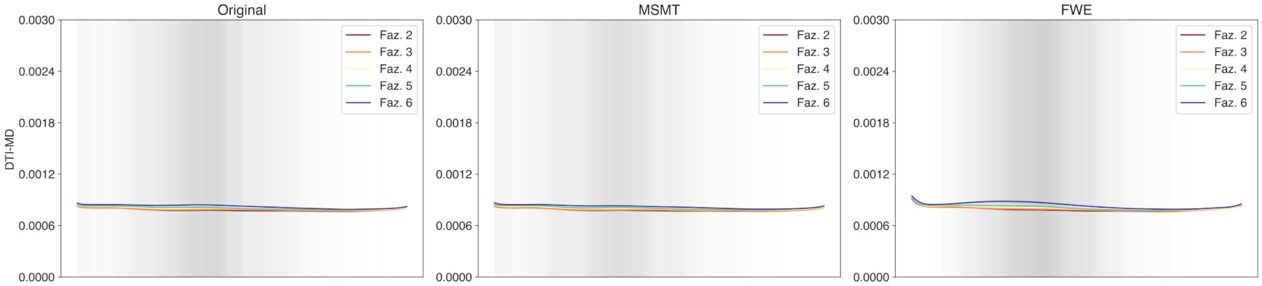

Supplemental Figure 179: Single-shell Left Posterior Arcuate Fasiculus DTI-MD profiles.

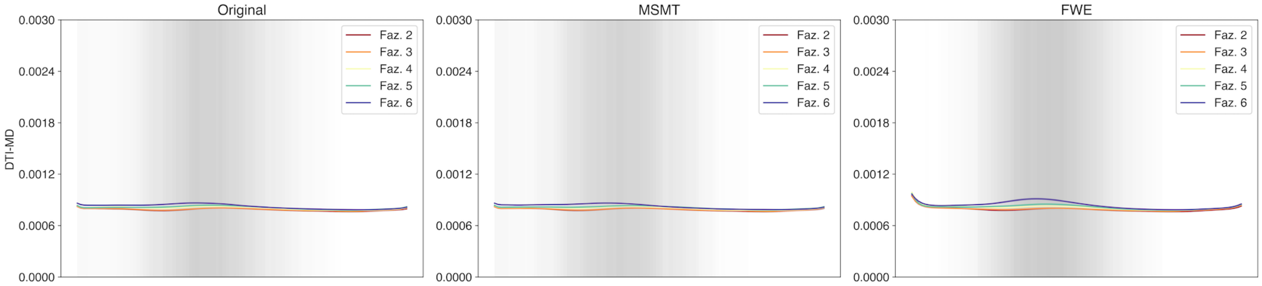

Supplemental Figure 180: Single-shell Right Posterior Arcuate Fasiculus DTI-MD profiles.

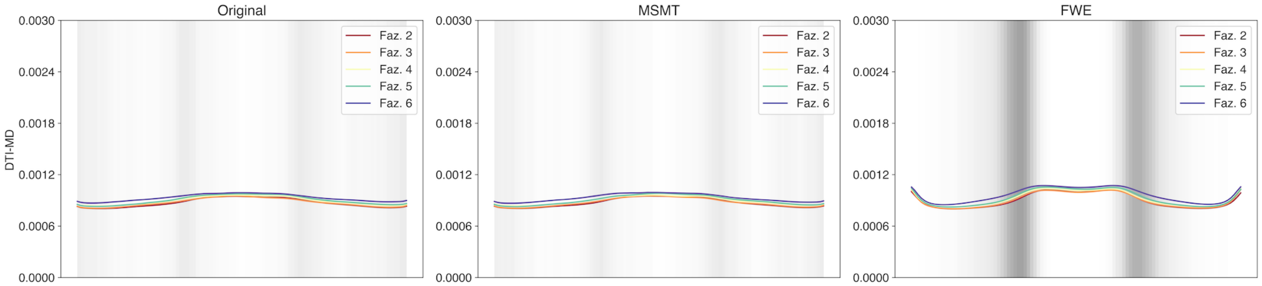

Supplemental Figure 181: Single-shell Anterior Frontal Callosum DTI-MD profiles.

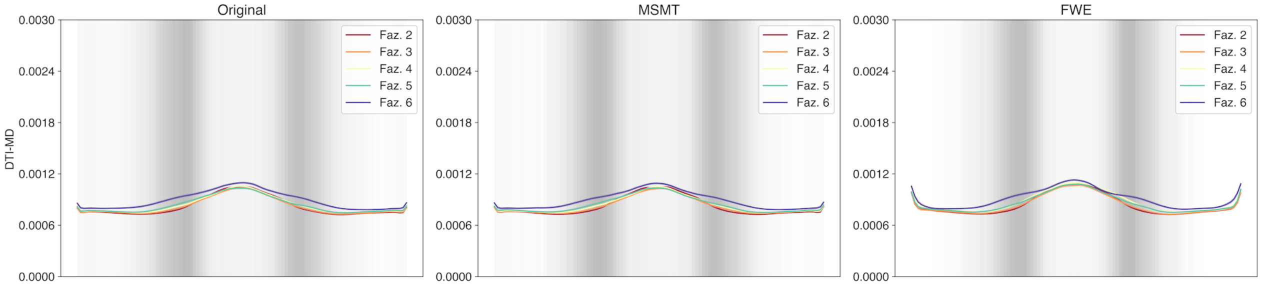

Supplemental Figure 182: Single-shell Motor Corpus Callosum DTI-MD profiles.

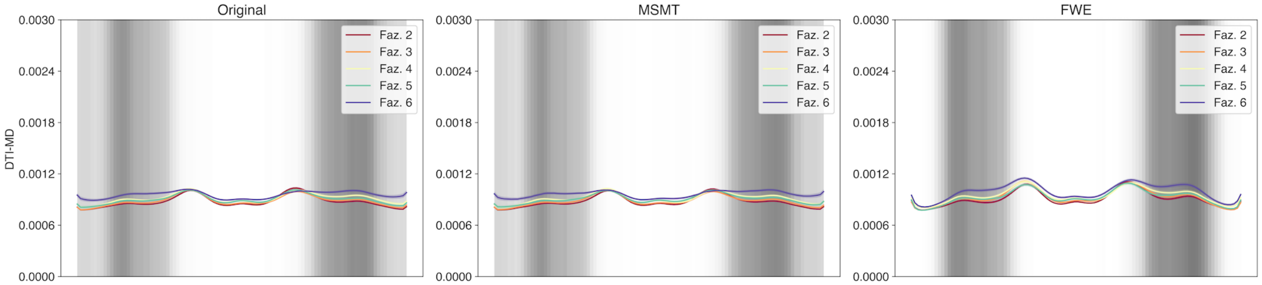

Supplemental Figure 183: Single-shell Occipital Corpus Callosum DTI-MD profiles.

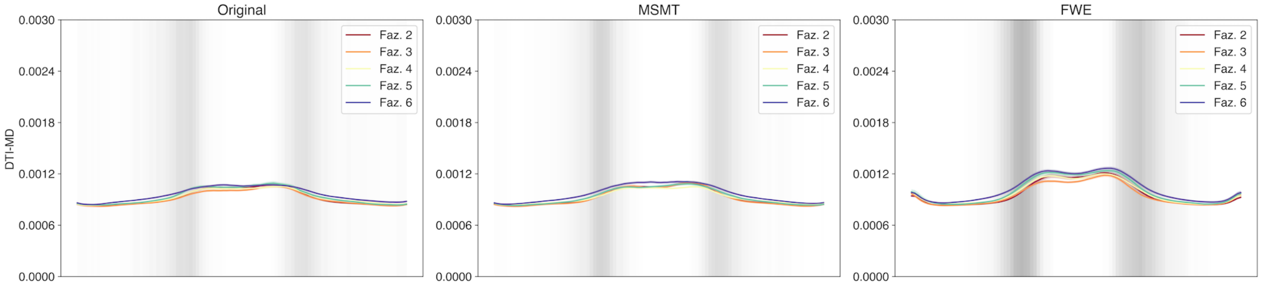

Supplemental Figure 184: Single-shell Orbital Corpus Callosum DTI-MD profiles.

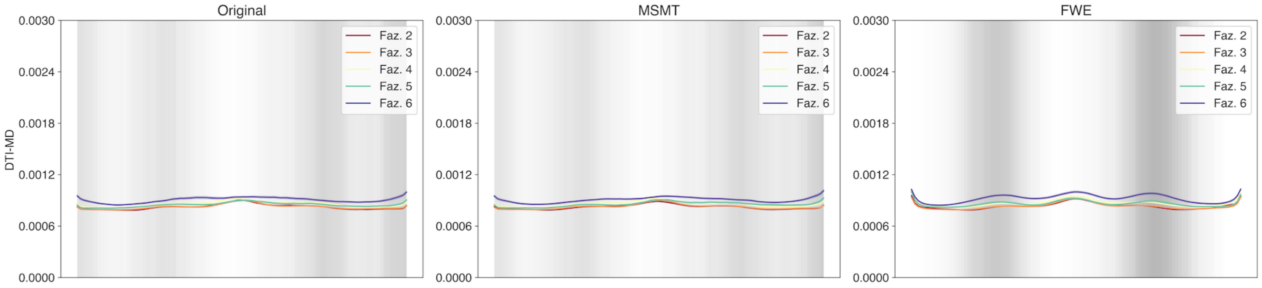

Supplemental Figure 185: Single-shell Posterior Parietal Corpus Callosum DTI-MD profiles.

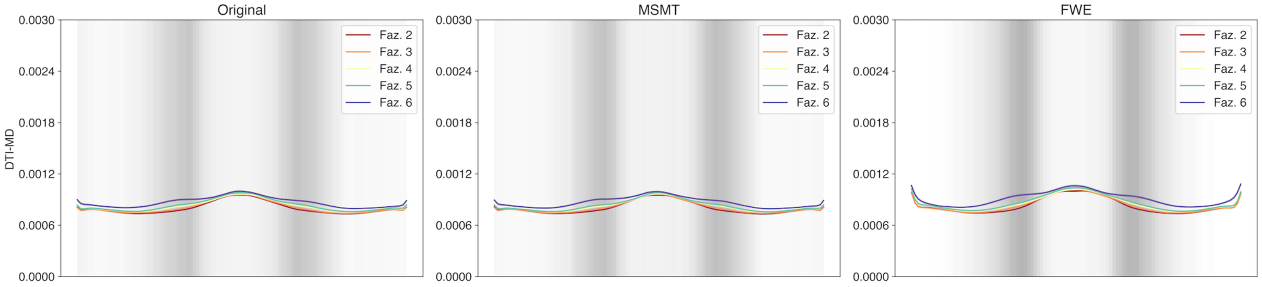

Supplemental Figure 186: Single-shell Superior Frontal Callosum DTI-MD profiles.

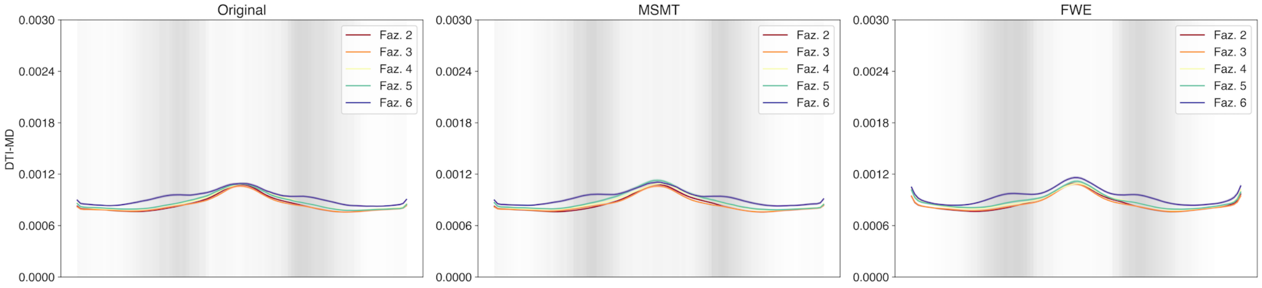

Supplemental Figure 187: Single-shell Superior Parietal Corpus Callosum DTI-MD profiles.

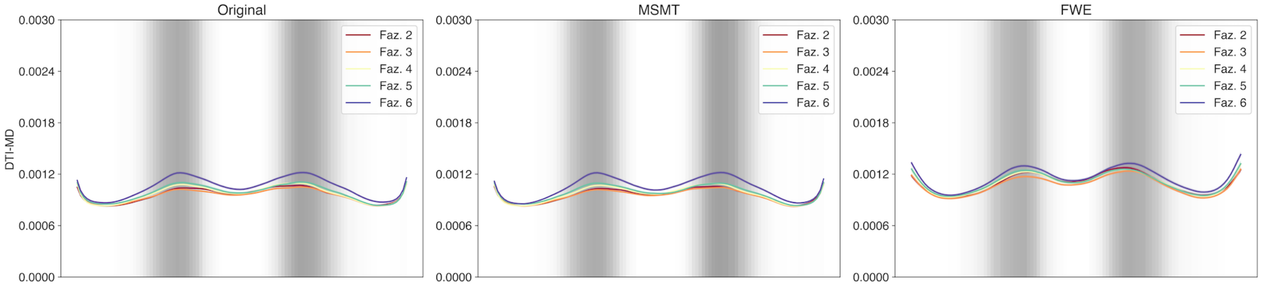

Supplemental Figure 188: Single-shell Temporal Corpus Callosum DTI-MD profiles.
